# Prognostic Factors for Mortality in Patients with Pyogenic Liver Abscess: A Systematic Review and Meta-Analysis

**DOI:** 10.7150/ijms.130156

**Published:** 2026-03-25

**Authors:** Jin-Wei Lin, Tai-Hung Ho, Min-Chi Shiang, Hao-Min Cheng, Yi-Tzu Lee, Chorng-Kuang How, Teh-Fu Hsu

**Affiliations:** 1 Department of Emergency Medicine, Taipei Veterans General Hospital, Taipei, 112201, Taiwan.; 2 Institute of Emergency and Critical Care Medicine, National Yang Ming Chiao Tung University, Taipei, 112304, Taiwan.; 3 School of Medicine, National Yang Ming Chiao Tung University, Taipei, 112304, Taiwan.; 4 Department of Emergency Medicine, China Medical University Hsinchu Hospital, Hsinchu, 302, Taiwan.; 5 Department of Nursing, Taipei Veterans General Hospital, Taipei, 112201, Taiwan.; 6 Division of Faculty Development, Department of Medical Education, Taipei Veterans General Hospital, Taipei, 112201, Taiwan.; 7 Ph.D. Program of Interdisciplinary Medicine, National Yang Ming Chiao Tung University, Taipei, 112304, Taiwan.; 8 Institute of Public Health, National Yang Ming Chiao Tung University, Taipei, 112304, Taiwan.; 9 Institute of Health and Welfare Policy, National Yang Ming Chiao Tung University, Taipei, 112304, Taiwan.; 10 Kinmen Hospital, Ministry of Health and Welfare, Kinmen, 891, Taiwan.; 11 Institute of Clinical Nursing, National Yang Ming Chiao Tung University, Taipei, 112304, Taiwan.

**Keywords:** pyogenic liver abscess, mortality, prognostic factors, drainage, sepsis

## Abstract

**Background:**

Pyogenic liver abscess (PLA) is an infectious and potentially fatal disease, yet no systematic reviews have comprehensively analyzed the prognostic factors associated with mortality. This study aims to identify prognostic factors associated with mortality in PLA.

**Methods:**

Databases including PubMed, Cochrane Library, Web of Science, Europe PMC, EMBASE, Airiti Library, LILACS, Google Scholar, ClinicalTrials.gov, and ICTRP were searched from inception to July 31, 2023. Reference lists, relevant reviews, and conference abstracts were also screened. Studies investigating predictors of mortality in PLA were included, with short-term mortality as the primary outcome. Pooled estimates were calculated using a random-effects model. Subgroup, meta-regression, and sensitivity analyses were performed.

**Results:**

Seventy-one observational studies were included in this systematic review, and 57 studies involving 126,056 patients contributed to the meta-analyses. Pooled adjusted estimates identified significant mortality predictors: older age, female sex, malignancy, chronic kidney disease, septic shock, higher APACHE II score, gas-formation, metastatic infection, anemia, thrombocytopenia, hypoalbuminemia, hyperbilirubinemia, elevated ALT, impaired renal function, bacteremia, *Escherichia coli* infection, anaerobic infection, multidrug-resistant organism infection, pneumonia, and ICU admission. Fever, *Klebsiella pneumoniae* infection, and percutaneous drainage were associated with lower short-term mortality.

**Conclusion:**

In PLA, significant mortality predictors included demographic, comorbidity, clinical, laboratory, radiographic, microbiological, and complication-related factors. Percutaneous drainage was associated with lower short-term mortality in selected patients, although this association should not be interpreted as causal, and treatment decisions should be individualized according to the underlying etiology and clinical context. Future high-quality prospective studies to identify etiology-specific prognostic factors are warranted.

## Introduction

Although diagnostic tools and clinical care have improved, pyogenic liver abscess (PLA) remains a potentially life-threatening infectious disease. The literature has reported a wide range of short-term mortality rates, with in-hospital mortality ranging from 0.6% to 11% 1-3.

Numerous studies have identified various prognostic factors for mortality among patients with PLA. Older age has been highlighted as a significant predictor of mortality 4, while some literature has found no significant correlation 5. Malignancy has been recognized as an important prognostic factor for fatal outcomes, but the estimates vary 6. Moreover, diabetes mellitus has been characterized as a key comorbidity in infection, but its role in clinical outcomes of PLA remains inconsistent 7. Additionally, the relationship between causative pathogens, their patterns of drug resistance, and disease prognosis has been explored, with heterogeneous findings observed 8, 9. While drainage has been acknowledged as a principal treatment modality, the comparative effects of percutaneous and surgical approaches on PLA remain uncertain 10.

Given the limitations of existing evidence and the paucity of high-quality and comprehensive research on prognostic predictors in PLA, this review sought to identify adjusted prognostic factors for mortality across different timeframes.

## Methods

This work adhered to the Preferred Reporting Items for Systematic Reviews and Meta-Analyses (PRISMA) (Table S1) and the Checklist for Critical Appraisal and Data Extraction for Systematic Reviews of Prognostic Factor Studies (CHARMS-PF) guidelines 11, 12. The study protocol has been registered in INPLASY with the identifier INPLASY202380006.

### Search strategy

Two reviewers independently searched eight databases (PubMed, Cochrane Library, Web of Science, Europe PMC, EMBASE, Airiti Library, LILACS, and Google Scholar) and two trial registries (ClinicalTrials.gov and the WHO International Clinical Trials Registry Platform [ICTRP]) from their inception through July 31, 2023. The reference lists of included studies and six related systematic reviews, as well as conference abstracts from three major annual meetings were also screened. The search strategy in detail was shown in Table S2.

### Eligibility criteria

Studies fulfilling the following criteria were enrolled: (1) studies of patients with PLA; (2) studies reporting factors associated with mortality and analyzed using logistic regression or Cox proportional hazards regression; and (3) observational designs. Furthermore, duplicate records, non-relevant studies, studies without full-text availability, non-human studies, case reports, studies not addressing the outcome of interest, and those reporting combined outcomes were excluded. There were no restrictions on language, publication period, or publication status. Preprints were eligible if they met the predefined inclusion criteria and provided sufficient methodological details and extractable data.

### Study selection

Based on the predefined eligibility criteria, two reviewers independently assessed article titles/abstracts and subsequently the full texts of the retrieved articles. Any discrepancies were resolved in consultation with a third reviewer. Publication timeframe, publication status, and language were not restricted.

### Outcomes

The primary outcome was short-term mortality, including in-hospital mortality, intensive care unit (ICU) mortality, 28-day or 30-day mortality, and post-discharge 30-day mortality. Secondary outcomes included other measures of long-term mortality.

### Data extraction

Two reviewers independently extracted relevant study data from each article. Any discrepancies were resolved in consultation with a third reviewer. To minimize potential duplication of patient populations, the characteristics of potentially related studies, including disease context, recruitment period, and study institutions, were examined. When potential overlap between studies was suspected for the same prognostic factor, only the study with the largest sample size was selected for inclusion in the meta-analysis. Studies assessing different prognostic factors from the same cohort were retained because they did not lead to duplication within each factor-specific analysis. Study characteristics were collected, and adjusted and unadjusted prognostic factors with their estimates were documented. When multiple models were available, we preferentially extracted adjusted estimates from the most fully adjusted multivariable model that accounted for most of the predefined core confounders—age, sex, chronic kidney disease (CKD), malignancy, and diabetes mellitus.

### Risk of bias assessment

The quality assessment of included studies was performed using the Quality in Prognostic Studies (QUIPS) tool by two independent reviewers 13. Any discrepancies were resolved in consultation with a third reviewer. The six domains for assessment are: (1) bias due to participation; (2) bias due to attrition; (3) bias due to prognostic factor measurement; (4) bias due to outcome measurement; (5) bias due to confounding; and (6) bias in statistical analysis and reporting. In the domain of bias due to confounding, the core confounders—specified as a predefined set of prognostic factors for adjustment—denote five major covariates: age, sex, CKD, malignancy, and diabetes mellitus, which were accounted for in the multivariate analysis 12. Based on these six domains, an overall high risk of bias was assigned if any domain was rated high risk or if four or more domains were rated moderate risk. For studies without any high-risk domains, the overall risk was classified as moderate when two to three domains were rated moderate risk, whereas those with at most one moderate-risk domain were classified as low risk.

### Statistical analysis

All statistical analyses were performed using Review Manager version 5.3.5 (The Nordic Cochrane Centre) and R version 4.3.1 within RStudio. Adjusted estimates of prognostic factors contributing to mortality from each study were pooled as the main results using a random-effects model with the inverse variance method in this meta-analysis. Outcomes were expressed as odds ratios (ORs) or hazard ratios (HRs) with corresponding 95% confidence intervals (CIs), based on the statistics reported in the included studies. For each prognostic factor evaluated using different regression models, separate meta-analyses were conducted for studies reporting ORs and HRs. Conference abstracts lacking sufficient methodological information to allow risk-of-bias assessment were excluded from the quantitative synthesis. Cochran's Q test and the I² statistic were used to examine heterogeneity. Egger's test was used to assess small-study effects only for meta-analyses including at least 10 studies. Funnel plots were also used to assess publication bias by examining the symmetry of the included studies. A *p*-value of less than 0.05 indicated statistical significance, and all analyses were conducted using two-sided tests.

Subgroup analyses of the primary outcome were conducted. For each prognostic factor, pooled unadjusted estimates were reported in sensitivity analyses when available. Univariate random-effects meta-regression analysis was performed to determine the association between study characteristics and the effect of malignancy on mortality.

### Certainty of evidence

The certainty of evidence for each prognostic factor was addressed separately through application of the Grading of Recommendations, Assessment, Development and Evaluations (GRADE) system by two independent reviewers 14, 15. For this systematic review of prognostic factors, a body of evidence from observational studies began as high certainty initially. The five GRADE domains for rating down certainty of evidence were risk of bias, imprecision, inconsistency, indirectness, and publication bias. Certainty could be rated up for two additional domains: dose-response gradient and large effect. Ultimately, certainty was categorized as high, moderate, low, or very low. Any discrepancies were resolved in consultation with a third reviewer.

## Results

### Study selection

The PRISMA flowchart of this systematic review is shown in Figure S1. Initially, 3,596 studies were identified from databases and registers, and another 2,363 from reference lists and conference abstracts. After exclusions, 71 studies were included in the systematic review, and 57 studies involving 126,056 individuals contributed to the meta-analyses.

### Study characteristics

Table 1 and Table S3 show the characteristics of these 71 observational studies, including 70 cohort studies and 1 case-control study. Their mean or median ages ranged from 50 to 72 years and the majority were male. Sixty-six articles reported primary outcomes with mortality rates ranging from 1% to 34%, and 5 studies described only secondary outcomes and could not be pooled in meta-analyses. Adjusted and unadjusted factors reported across the included 71 articles were provided in Table S4. Of them, 66 articles documented both adjusted and unadjusted estimates of prognostic factors, and 5 presented only unadjusted results. Among the 66 articles employing multivariate analyses, 65% incorporated adjustments for at least two core confounders. Fifty-seven studies contributed to the quantitative synthesis, and all pooled meta-analyses were derived from studies reporting ORs as effect measures. Studies reporting HRs addressed different prognostic factors or timeframes and therefore could not be pooled quantitatively.

### Risk of bias assessment

The comprehensive appraisal of risk of bias for individual studies is illustrated in detail in Figure S2. Assessment using the QUIPS tool revealed that 34% of studies had moderate or high risk of bias from participation, 58% from prognostic factor measurement, 37% from confounding, and 80% from statistical analysis and reporting. Only 6% and 15% of studies were judged as having moderate risk exclusively due to attrition and outcome measurement, respectively, with no studies rated as high risk in these two domains. In terms of overall judgment, 21% of studies were rated as low risk of bias, 48% as moderate, and 31% as high.

### Prognostic factors for primary outcome

A total of 66 enrolled studies reported predictors of the primary outcome, and 57 of them were meta-analyzed for different factors. Table 2 presents the pooled estimates of all factors for short-term mortality among patients with PLA, and Table 3 provides the GRADE summary of findings. Subgroup analyses are summarized in Table S5, and sensitivity analyses according to adjustment status are detailed in Table S6.

### Demographic data

For age as a categorical variable, older patients had a higher pooled estimate of short-term mortality compared with younger patients, based on 9 studies enrolling 3,048 individuals (aOR 2.33, 95% CI 1.29-4.19) (Figure S3A). Cochran's Q test for heterogeneity disclosed an *I²* statistic of 77% with p < 0.001. The subgroup analyses showed no significant differences for age threshold, outcome type, risk of bias, or country, but significant differences for year of publication and sample size. The sensitivity analysis demonstrated that the pooled result from unadjusted estimates (OR 1.97, 95% CI 1.29-3.01) was similar to that from adjusted estimates. The funnel plot showed no obvious asymmetry (Figure S3C). Its GRADE certainty was rated high. For age as a continuous variable (Figure S3B), the aOR per 1-year increase in age was 1.02 (95% CI 1.01-1.04).

A total of six studies with 80,403 participants were included in the meta-analysis of sex as a predictor of mortality. Females had a higher mortality rate than males (aOR 1.18, 95% CI 1.04-1.33, p = 0.01) (Figure S4). Statistical significance of heterogeneity was observed among these trials (*I²* = 66%, p = 0.01), but the majority of point estimates lay on the same side of the null effect threshold. The GRADE certainty was downgraded to moderate due to imprecision.

### Comorbidities

**Figure 1:**
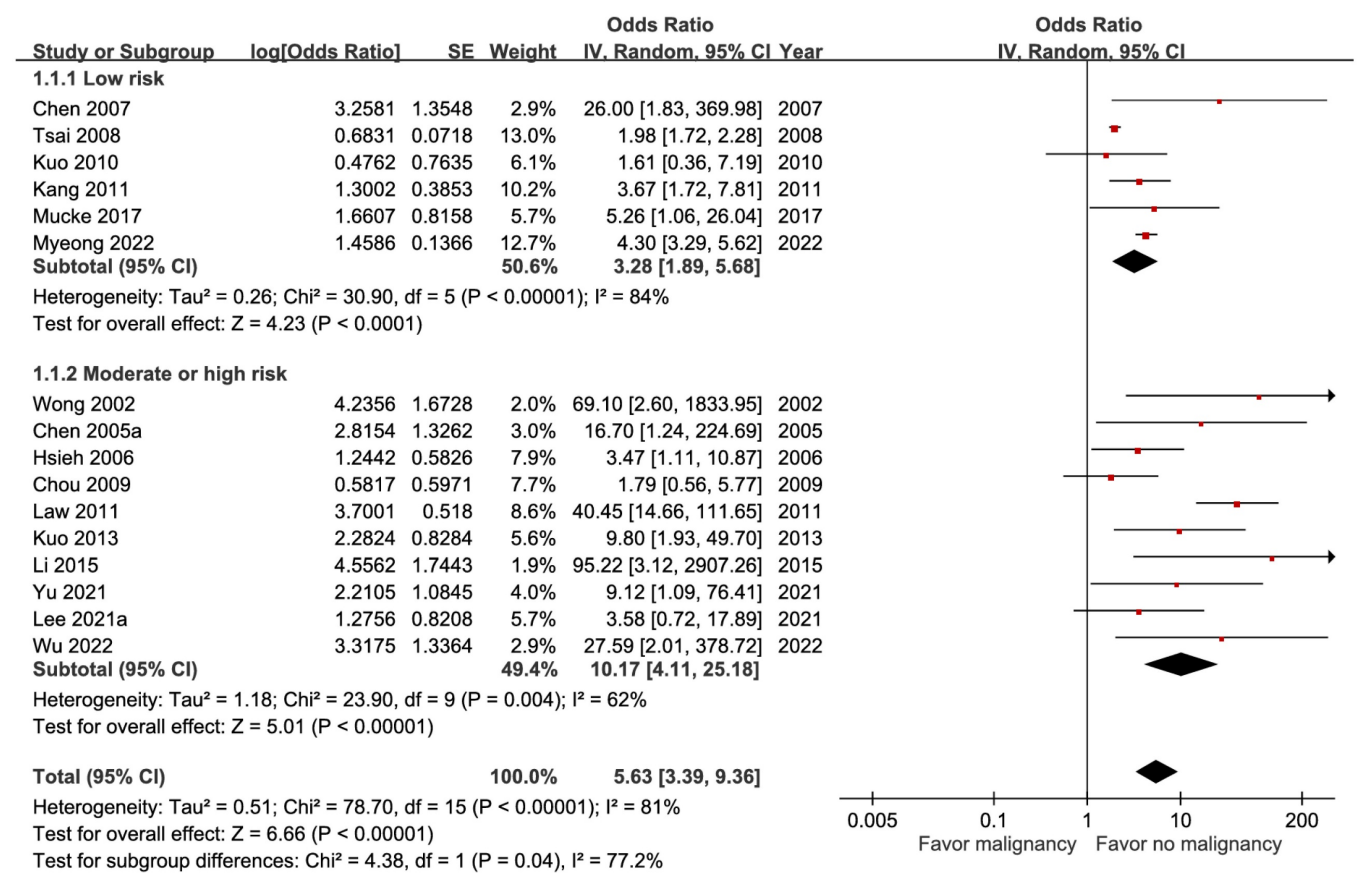
Forest plot of the association between malignancy (subgroup by risk of bias) and short-term mortality in pyogenic liver abscess.

The forest plot of malignancy predicting mortality from 16 studies including 65,972 patients showed an aOR of 5.63 (95% CI, 3.39-9.36) (Figure 1). Univariate random-effects meta-regression for the prognostic effect of malignancy (Table S7) revealed that low risk of bias and younger age were both significant predictors of its association with decreased mortality. The funnel plot (Figure S5) showed obvious asymmetry and the GRADE certainty was moderate.

**Figure 2:**
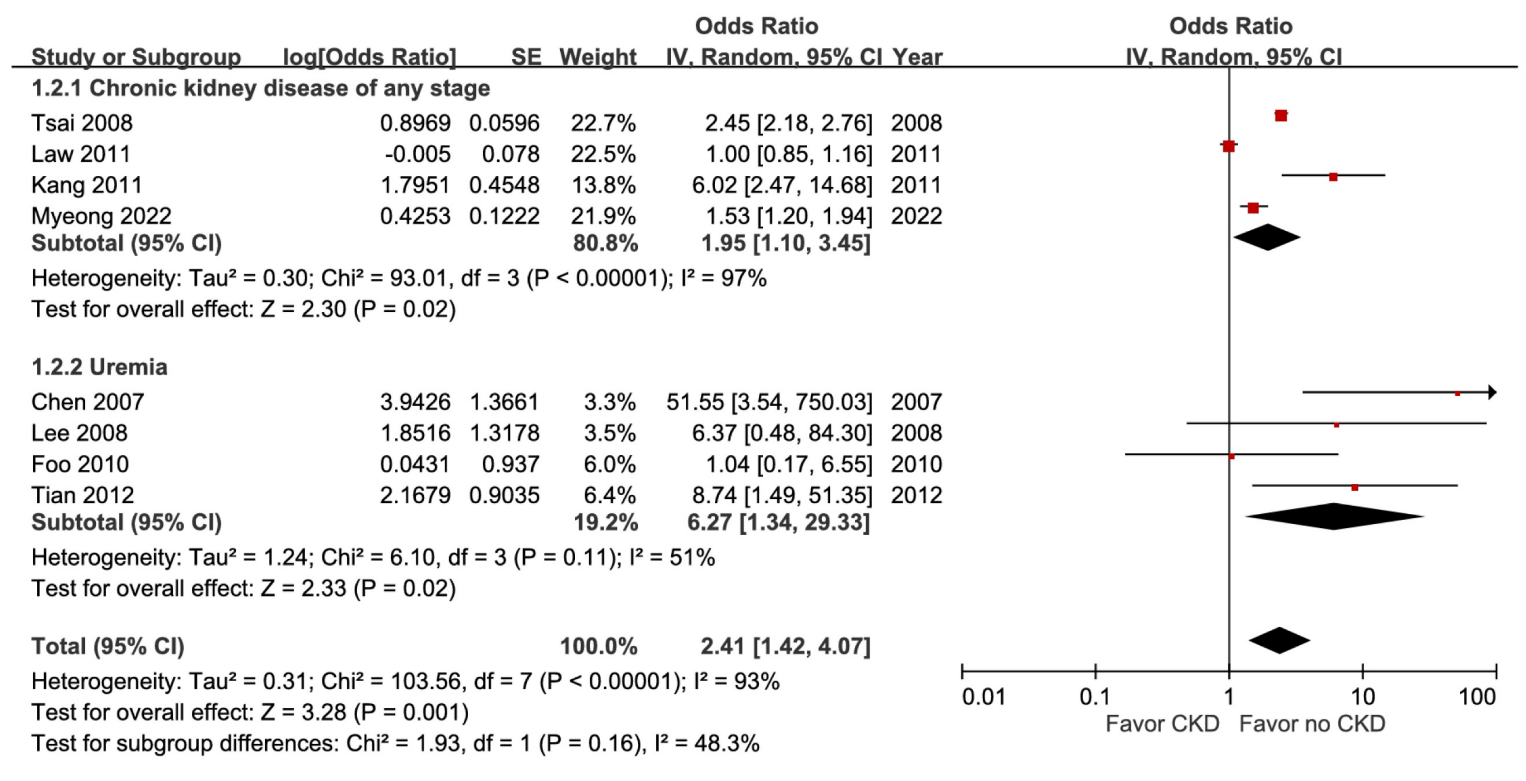
Forest plot of the association between chronic kidney disease (subgroup by severity) and short-term mortality in pyogenic liver abscess.

A pooled analysis of eight studies with 64,077 subjects indicated a statistically significant association between CKD and mortality (aOR 2.41, 95% CI 1.42-4.07, p = 0.001). Subgroup analysis identified a disease-severity-related gradient in point estimates (Figure 2), and the certainty of evidence was high.

Six studies including 62,349 patients examined the association between diabetes mellitus and mortality, and no significant effect was observed (aOR 1.06, 95% CI 0.83-1.36) (Figure S6). The heterogeneity was significant (*I*^2^ = 80%, *p* = 0.0001). The certainty of evidence was very low. There was also no significant association between cirrhosis or hypertension and mortality (Figure S7 and Figure S8).

### Clinical presentation and severity score

**Figure 3:**
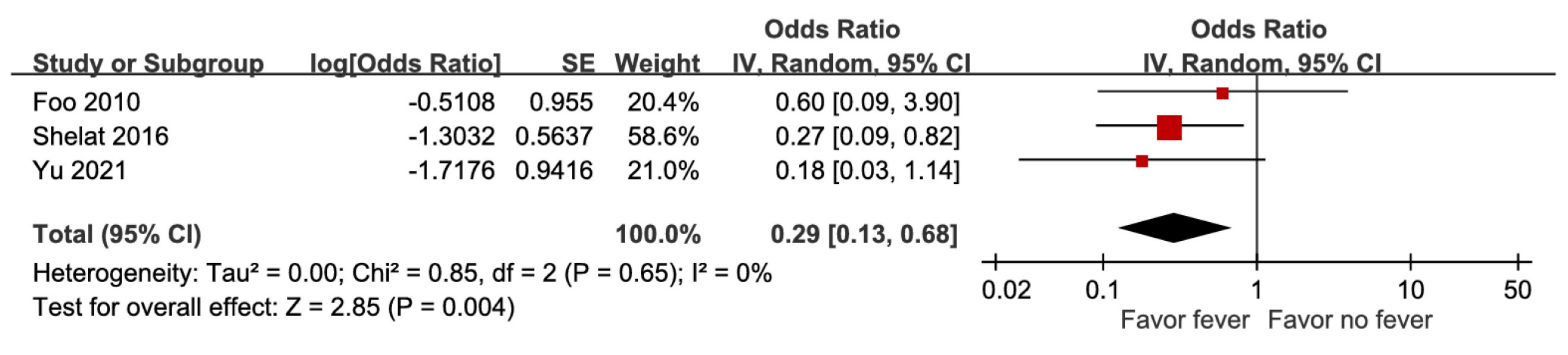
Forest plot of the association between fever and short-term mortality in pyogenic liver abscess.

Data from three trials showed that fever was a prognostic factor associated with decreased mortality, with an aOR of 0.29 (95% CI 95% CI 0.13-0.68, p = 0.004) (Figure 3). A high percentage of moderate-to-high risk of bias was noted, and sensitivity analysis based on adjustment revealed a discrepancy. Hence, the certainty of evidence was low.

**Figure 4:**
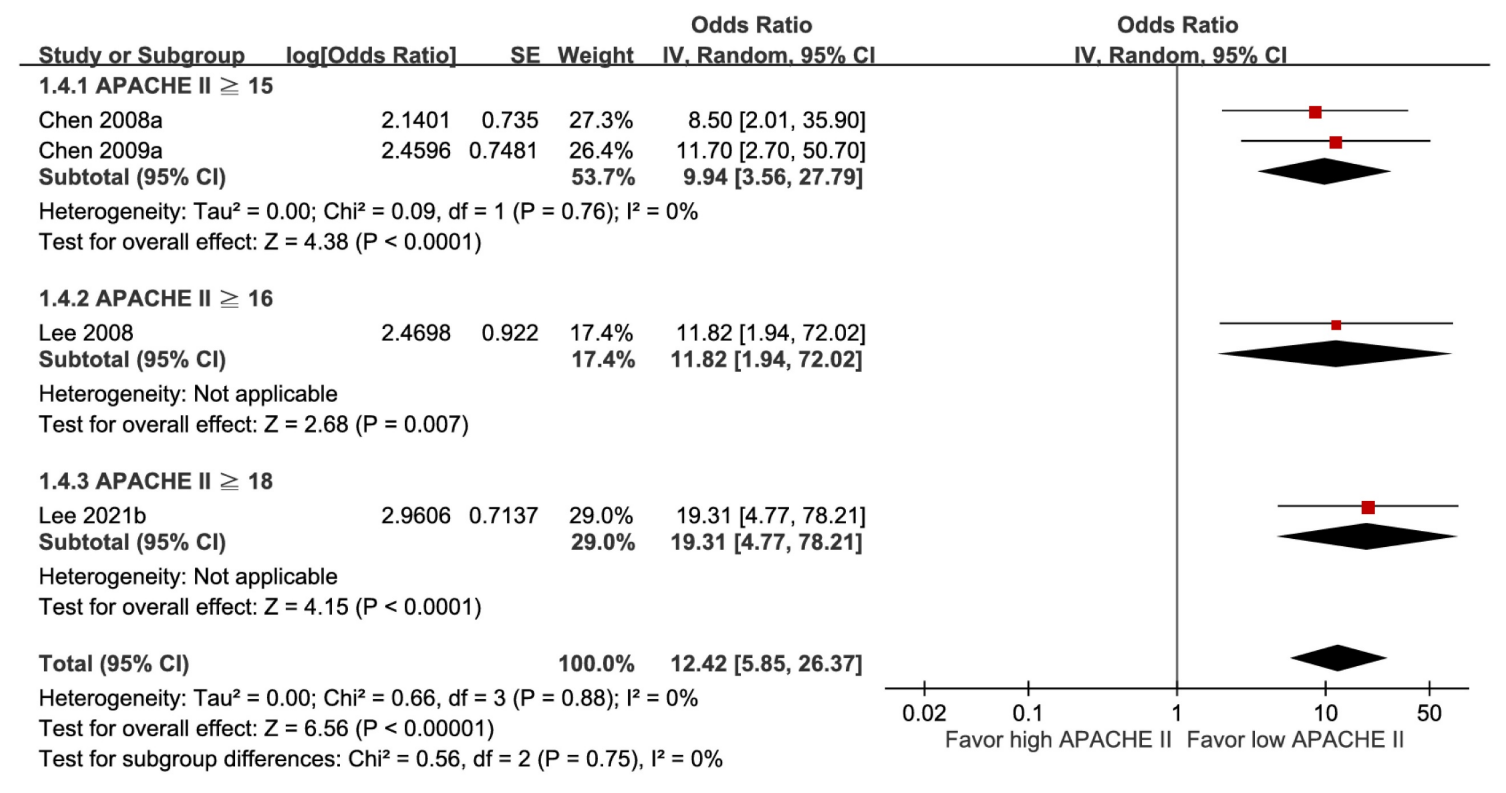
Forest plot of the association between APACHE II score (subgroup by cut-off value) and short-term mortality in pyogenic liver abscess.

Irrespective of whether the Acute Physiology and Chronic Health Evaluation II (APACHE II) score was treated as a categorical or continuous factor, a higher score was associated with increased mortality (aOR 12.42, 95% CI 5.85-26.37; and aOR 1.34, 95% CI 1.21-1.48) (Figure 4 and Figure S9A). As a categorical variable, the cut-off value showed a dose-response gradient relationship (Figure 4), whereas as a continuous variable, the funnel plot illustrated asymmetry (Figure S9B). As a result, their GRADE certainties were rated as high and moderate, respectively.

**Figure 5:**
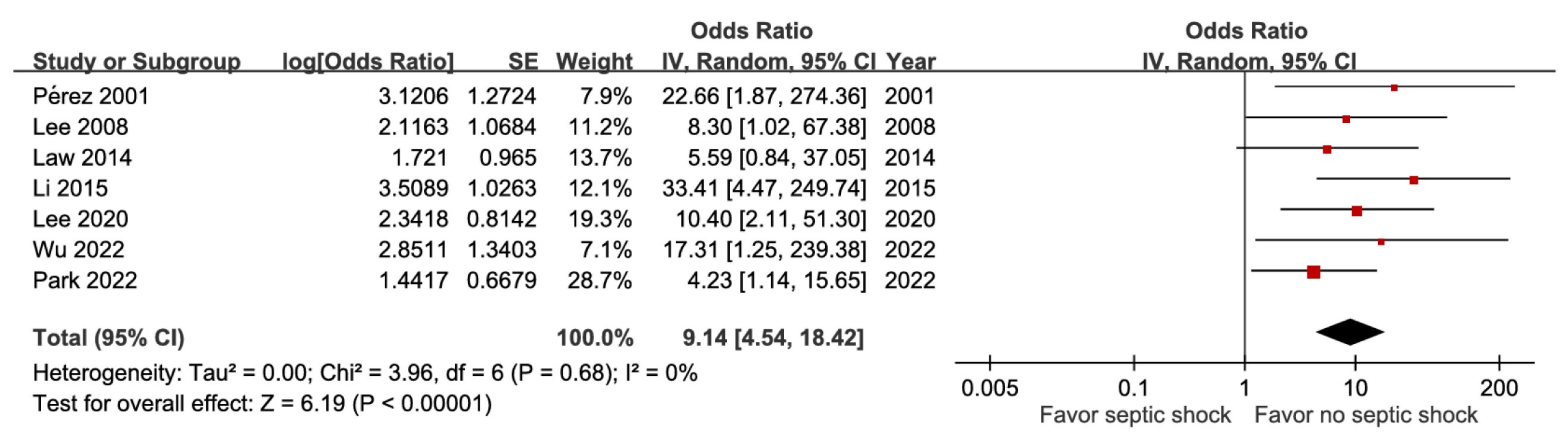
Forest plot of the association between septic shock and short-term mortality in pyogenic liver abscess.

A total of seven studies including 2,046 participants demonstrated a positive relationship between septic shock and poor outcome (aOR 9.14, 95% CI 4.54-18.42), without evidence of heterogeneity (I² = 0%) (Figure 5). The GRADE certainty was rated as high. The combined results showed that jaundice and altered mental status did not correlate with mortality (Figure S10 and Figure S11).

### Abscess pattern

**Figure 6:**
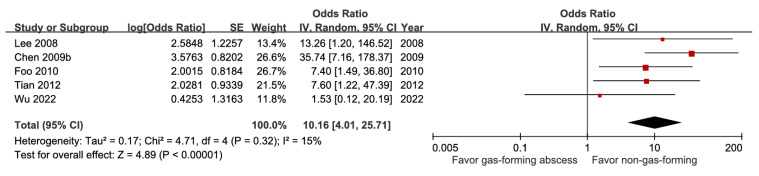
Forest plot of the association between gas-forming abscess and short-term mortality in pyogenic liver abscess.

For biliary origin, abscess size, or multiple abscesses, there was no significant effect on prognosis (Figure S12, Figure S13, and Figure S14). However, year of publication was a significant subgroup factor for both abscess size and multiple abscesses. The GRADE certainties for these factors were downgraded to moderate to very low, mainly due to imprecision. Gas-forming abscess was identified as a prognostic factor for mortality, with an aOR of 10.16 (95% CI 4.01-25.7), and this evidence had a moderate degree of certainty (Figure 6).

### Laboratory findings

Anemia and thrombocytopenia were predictive of mortality, with aORs of 4.33 (95% CI 1.05-17.91) and 4.18 (95% CI 2.05-8.50) (Figures S17 and Figure S18), in which a large effect was observed for both GRADE ratings. Nevertheless, no significant association with mortality was observed for serum white blood cell (WBC) count, analyzed either continuously or categorically (Figures S15 and Figure S16).

**Figure 7:**
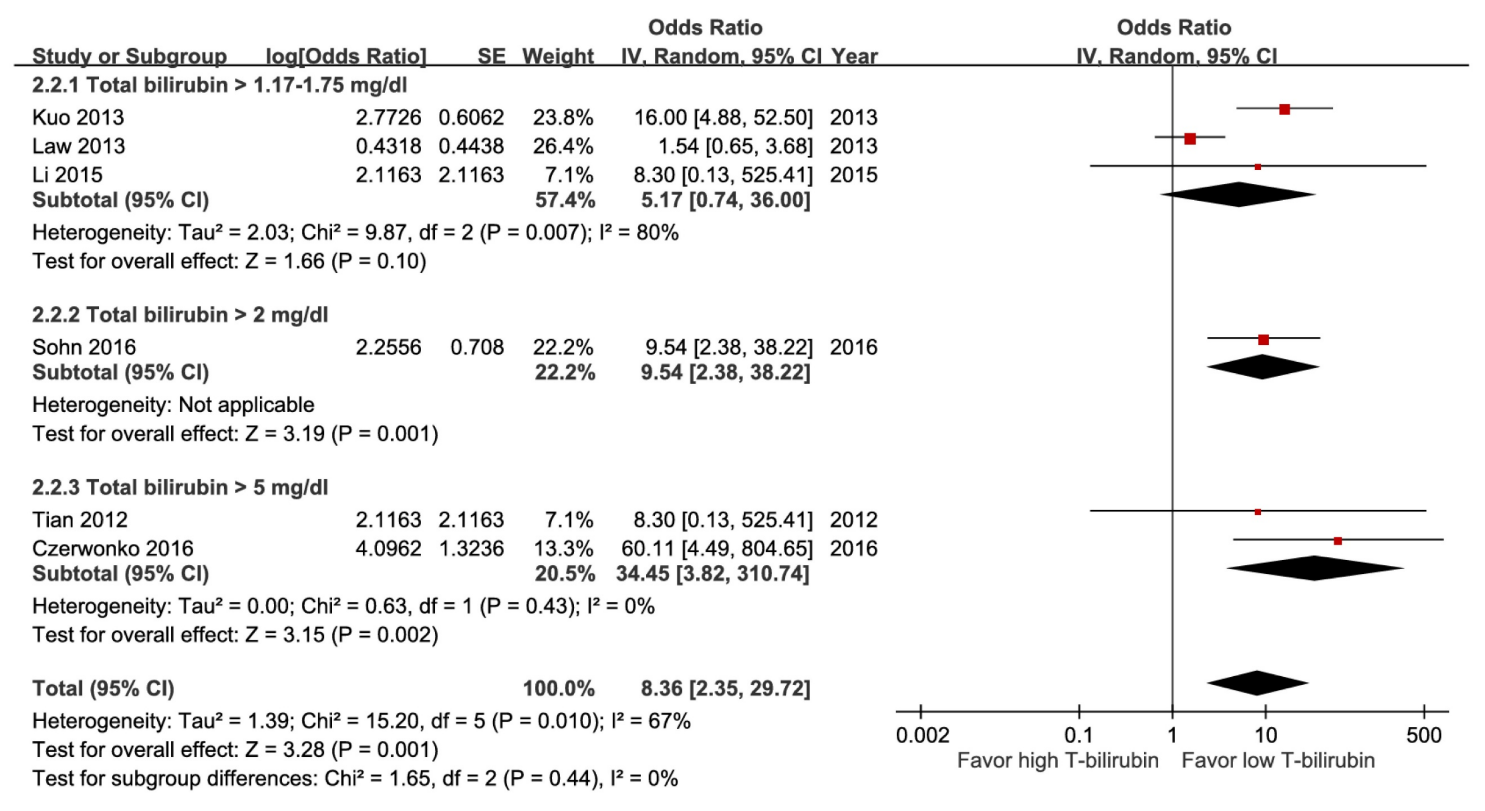
Forest plot of the association between hyperbilirubinemia (subgroup: cut-off value) and short-term mortality in pyogenic liver abscess.

**Figure 8:**
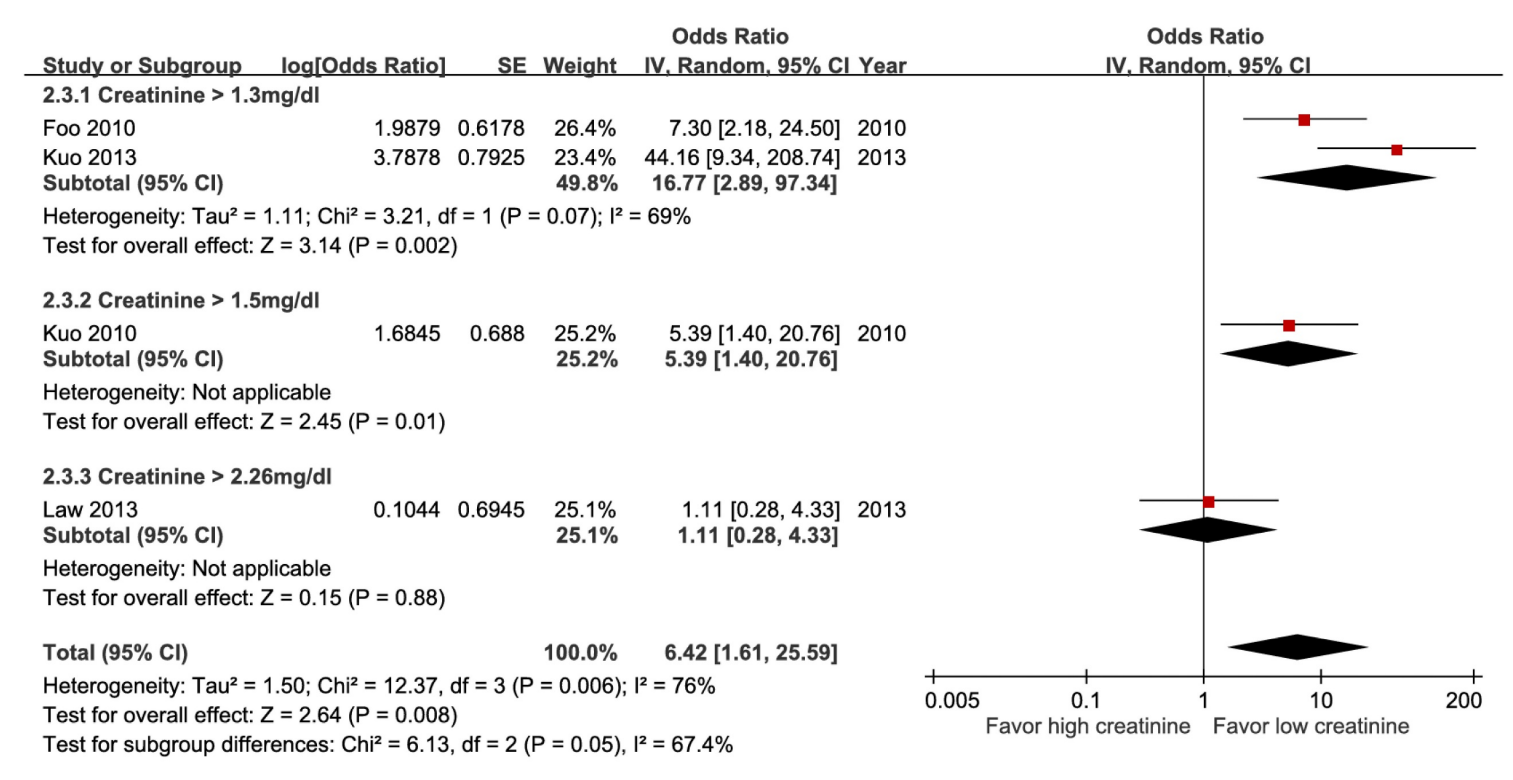
Forest plot of the association between impaired renal function (subgroup: cut-off value) and short-term mortality in pyogenic liver abscess.

**Figure 9:**
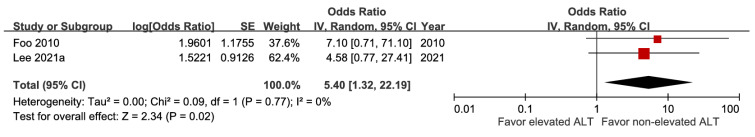
Forest plot of the association between elevated ALT and short-term mortality in pyogenic liver abscess.

Among these meta-analyses, participants with hypoalbuminemia (Figure S19), hyperbilirubinemia (Figure 7), impaired renal function (Figure 8), elevated alanine aminotransferase (ALT) (Figure 9), or azotemia (Figure S20) had a higher risk of mortality than those without. The pooled estimate for hypoalbuminemia from eight studies was aOR 4.12 (95% CI, 2.60-6.53). In the sensitivity analysis excluding one preprint, the pooled result remained unchanged (aOR 4.05; 95% CI, 2.46-6.67) (Table S8) 16. However, the funnel plot of the standard error of the log odds ratio versus the odds ratio for hypoalbuminemia illustrated an asymmetrical distribution on visual inspection (Figure S19). Nevertheless, subgroup analysis of hyperbilirubinemia using different cut-off values demonstrated a dose-response gradient (Figure 7).

### Microbiological factors

**Figure 10:**
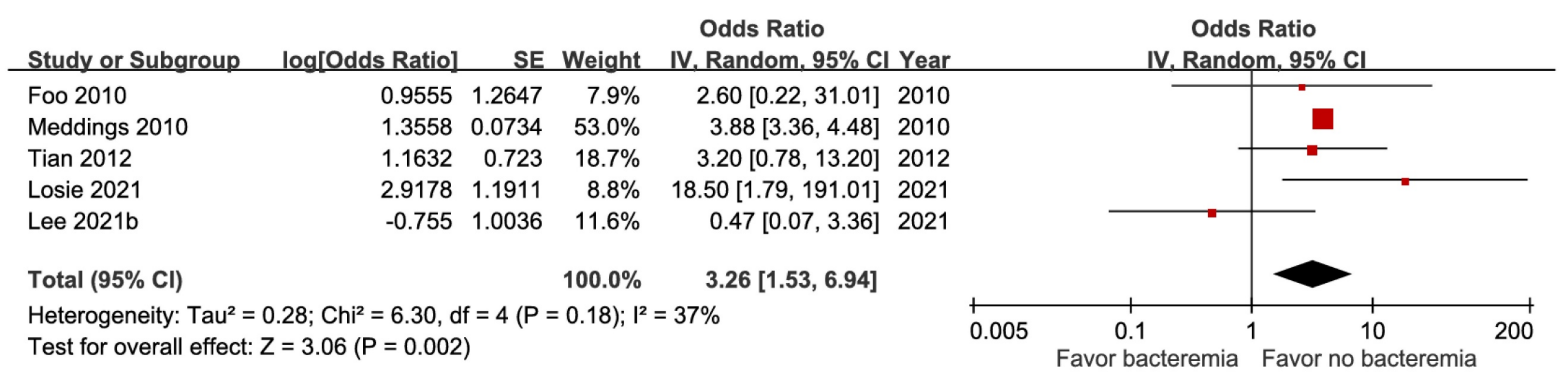
Forest plot of the association between bacteremia and short-term mortality in pyogenic liver abscess.

**Figure 11:**
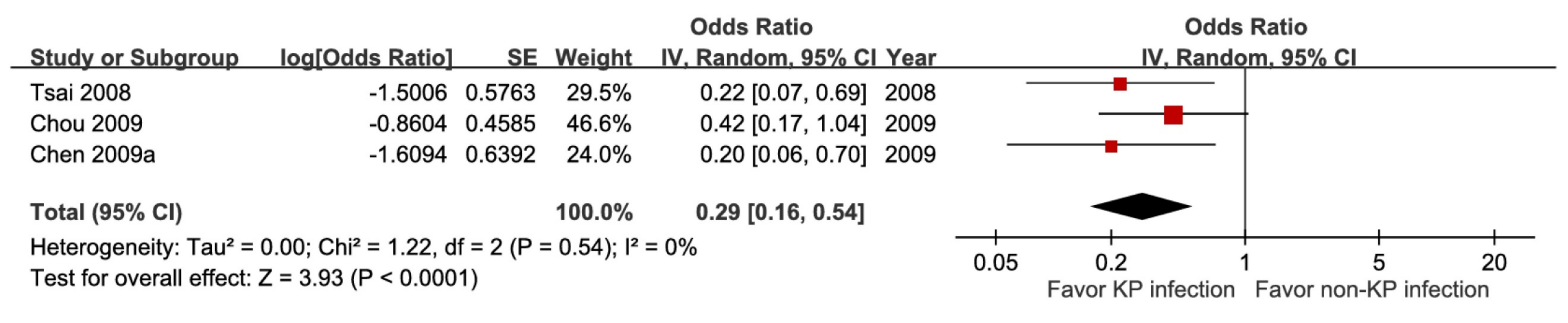
Forest plot of the association between *Klebsiella pneumoniae* infection and short-term mortality in pyogenic liver abscess.

**Figure 12:**
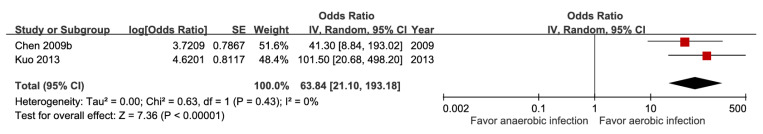
Forest plot of the association between anaerobic infection and short-term mortality in pyogenic liver abscess.

**Figure 13:**
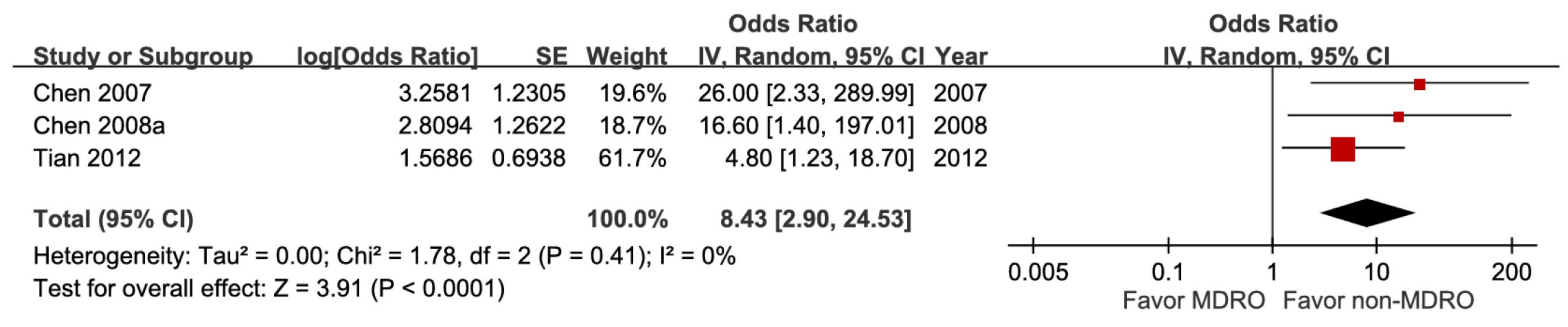
Forest plot of the association between multidrug-resistant organism and short-term mortality in pyogenic liver abscess.

Five studies involving 18,981 patients demonstrated that bacteremia was associated with increased short-term mortality (aOR 3.26, 95% CI 1.53-6.94), with high certainty of evidence (Figure 10). The pooled estimates of 30,399 participants from three studies evaluating *Klebsiella pneumoniae* infection showed a significant inverse association (aOR 0.29, 95% CI 0.10-0.54) (Figure 11). In contrast, *Escherichia coli* infection (aOR 2.84, 95% CI 1.30-6.21) and anaerobic infection (aOR 63.84, 95% CI 21.10-193.18) were significantly associated with increased mortality (Figure S21 and Figure 12). Polymicrobial infection was not associated with mortality (aOR 2.09, 95% CI 0.73-5.90) (Figure S22), whereas infection with multidrug-resistant organisms was associated with higher mortality (aOR 8.43, 95% CI 2.90-24.50) (Figure 13).

### Complications

**Figure 14:**
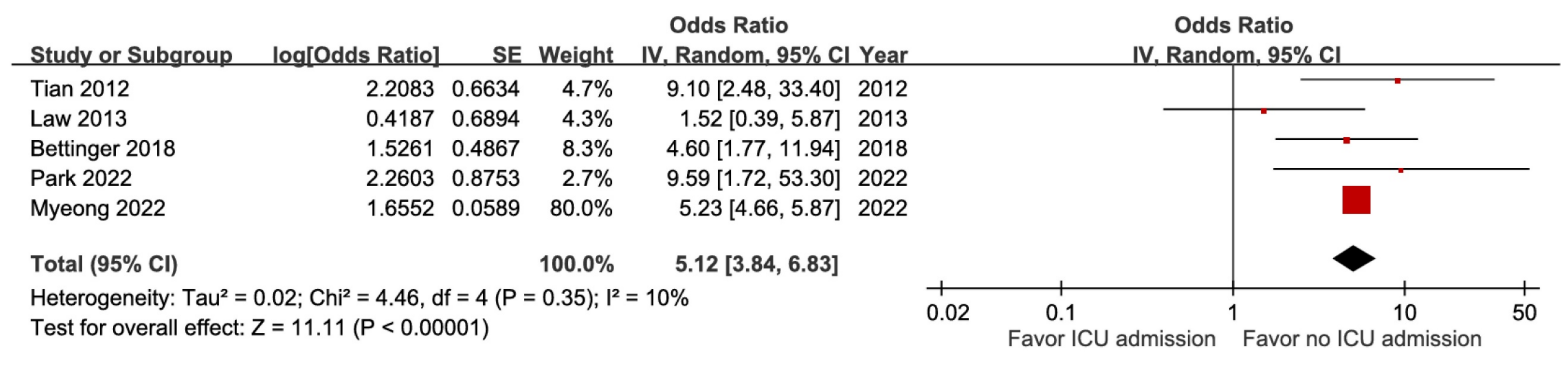
Forest plot of the association between ICU admission and short-term mortality in pyogenic liver abscess.

**Figure 15:**
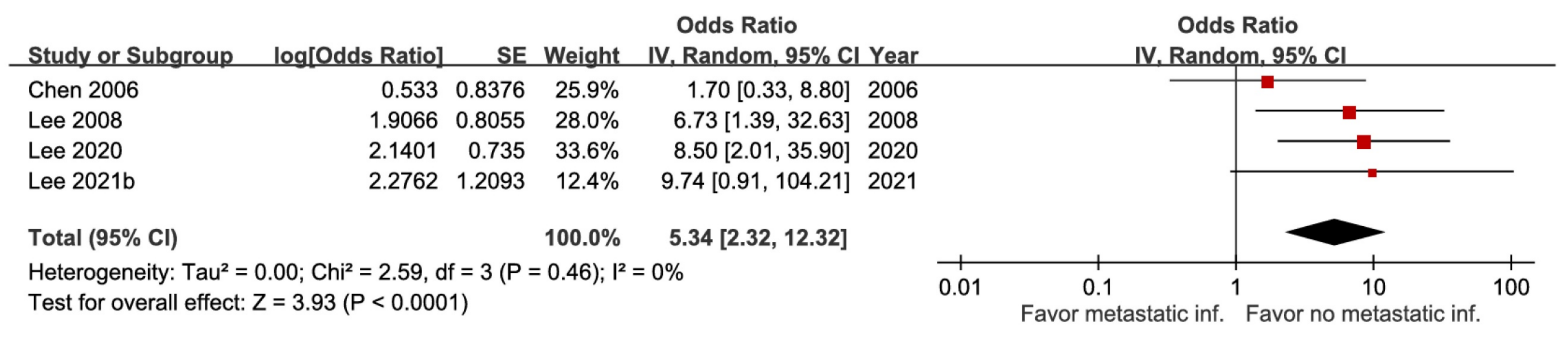
Forest plot of the association between metastatic infection and short-term mortality in pyogenic liver abscess.

Patients with pneumonia had a higher likelihood of fatal outcomes (aOR 1.52, 95% CI 1.33-1.72) (Figure S23), whereas both ICU admission and metastatic infection showed stronger associations (aOR 5.12, 95% CI 3.84-6.83, high certainty; aOR 5.34, 95% CI 2.32-12.32, moderate certainty) (Figure 14 and Figure 15). With very low certainty, ascites was not significantly correlated with mortality (Figure S24).

### Treatment modalities

**Figure 16:**
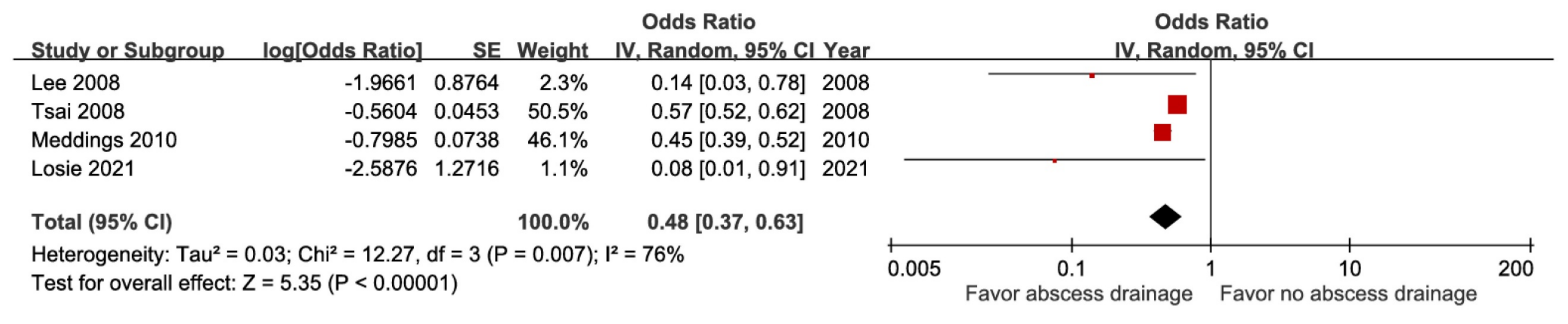
Forest plot of the association between percutaneous drainage and short-term mortality in pyogenic liver abscess.

A meta-analysis of four studies including 47,736 subjects demonstrated a lower risk of mortality among patients treated with percutaneous drainage compared with those not treated (aOR 0.48, 95% CI 0.37-0.63) (Figure 16). Nonetheless, the pooled estimate from two studies enrolling 18,105 patients on surgical drainage indicated no significant association (aOR 0.92, 95% CI 0.74-1.15; moderate GRADE certainty) (Figure S25).

### Prognostic factors for secondary outcomes

A total of five eligible studies described prognostic factors for the secondary outcomes, but meta-analysis could not be conducted because of differences in timeframes, statistical methods, or factors analyzed.

## Discussion

This comprehensive systematic review of prognostic studies, conducted in accordance with the PRISMA and CHARMS-PF guidelines, enrolled 71 observational studies examining prognostic factors for mortality in patients with PLA identified through eight databases, two trial registries, reference lists, and conference abstracts. With risk of bias assessed using the QUIPS tool, 57 articles involving 126,056 subjects were included in meta-analysis of predictors for short-term mortality, validated by subgroup analyses, sensitivity analyses according to adjustment status, meta-regression, and GRADE evaluation. Demographic characteristics, comorbidities, clinical presentation, abscess patterns, laboratory findings, and microbiological variables were identified as prognostic factors, whereas complications and treatment modalities were identified as factors associated with mortality.

One review article demonstrated the higher mortality rate in patients with PLA of biliary origin than that of PLA of nonbiliary origin, inconsistent with our findings 17. Many studies have emphasized the importance of specific etiologic factors for clinical outcomes in patients with PLA 18-20. However, according to methodological guidance for systematic reviews of prognostic factor studies, prognostic associations should ideally be derived from multivariable regression models, such as logistic regression or Cox proportional hazards regression, which allow estimation of the independent prognostic contribution of a factor after adjustment for other established prognostic variables 12. Under these methodological considerations, the number of eligible studies addressing specific etiological factors was limited. Therefore, the above-mentioned literature on etiologic factors was not included in our study because it did not meet our methodological criteria. During our systematic search, most included studies reported demographic, clinical, laboratory, or microbiological prognostic factors, but did not consistently stratify analyses according to detailed etiological subgroups, particularly hepatic artery injury/ischemia or post-transplant ischemic complications. Consequently, a quantitative meta-analysis focusing on these etiological categories was not feasible in this study. Therefore, this evidence gap and methodologic considerations were acknowledged in this prognostic systematic review and highlight the need for further high-quality studies to address this issue.

In this study, short-term mortality was defined to include closely related early mortality endpoints, including in-hospital mortality, ICU mortality, 28- or 30-day mortality, and post-discharge 30-day mortality. These outcomes are commonly considered comparable short-term outcomes in studies of infectious disease. Research on septic shock disclosed highly comparable pooled estimates for ICU mortality (37.3%), in-hospital mortality (39.0%), and 28/30-day mortality (36.7%), suggesting that these endpoints reflect closely related measures of early mortality 21. In systematic reviews of sepsis and liver abscess, mortality outcomes are frequently reported using different but closely related short-term endpoints, including in-hospital mortality, 28-day mortality, 30-day mortality, 6-week mortality, and post-discharge mortality within 30 or 90 days 22-24. Although the exact follow-up duration varies across studies, these endpoints primarily capture deaths occurring within the early clinical course of infection and are therefore regarded as comparable measures of short-term prognosis. This approach follows established methodological guidance for systematic reviews of prognostic factors, which recognizes that outcome definitions may vary across studies and may require harmonization when they represent the same clinical concept 12. Additionally, subgroup analyses stratified by outcome timeframe were performed (Table S5) for several factors, including female sex, diabetes mellitus, higher APACHE II score, abscess size, and azotemia; most showed no significant differences between subgroups in this study.

For demographic characteristics, older age and female sex were significant prognostic factors for mortality in this research. Irrespective of being treated as a categorical or continuous variable, age was shown to have a strong association with prognosis in both unadjusted and adjusted models. This finding was consistent with previous literature indicating that older age is associated with poor prognosis in sepsis and infectious diseases 25. Generally speaking, males may exhibit higher mortality and worse prognoses than females in various diseases, potentially because of lower healthcare utilization, inadequate treatment adherence, the effects of female sex hormones, and lifestyle determinants 26. In contrast, some research still demonstrated more unfavorable outcomes in females than in males, consistent with our findings 27. Although statistical heterogeneity was noted in our forest plot (Figure S4), the point estimates of the included studies consistently lay in the same direction relative to the threshold of the null effect. Hence, the plausible mechanisms through which female sex serves as a prognostic factor merit more biological and epidemiological exploration.

The significant comorbidities related to patients' fatal outcomes detected in our study were malignancy and CKD, but not diabetes mellitus. Previous literature also listed advanced malignancy as a prognostic factor for worse survival in sepsis 28. Geng et al. stated that PLA in pancreatic cancer patients with biliary stents requires prolonged antibiotic regimens due to a high rate of polymicrobial infections, immunocompromised status, and delayed abscess resolution 29. Our meta-regression analysis indicated that studies with low risk of bias and populations of younger mean age may show a decreased predictive effect on mortality. CKD has also been described as an independent predictor of adverse outcomes in literature 30. Furthermore, our subgroup analysis found that participants with uremia undergoing renal replacement therapy had higher pooled estimates of short-term mortality than those with any stage of CKD (aOR 6.27 vs. 1.95), demonstrating a dose-response gradient that led to an upgraded GRADE level. However, our study did not observe a significant effect of diabetes mellitus on mortality, even with very low certainty of evidence according to GRADE. This finding contrasts with longstanding perspectives and prior research, which have linked diabetes to adverse outcomes among adult sepsis survivors 31. Emerging evidence highlights that diabetes mellitus status alone does not impair the prognosis of septic patient 24. Instead, metrics of glycemic control, such as HbA1c, glycemic variability, and antidiabetic medication use, may be more strongly associated with mortality than diabetes status itself 22. Potential confounding by diabetes treatment effects or glycemic control was not uniformly adjusted for in the included studies; therefore, further research with adequate adjustment is needed in the future.

Potential heterogeneity arising from variations among different regions was also an important consideration in this investigation. We conducted subgroup analyses of prognostic factors according to study country (Asian vs. non-Asian), and no significant subgroup differences were observed (Table S5). Within this prognostic systematic review, the three studies investigating *Klebsiella pneumoniae* infection were exclusively performed in Taiwan, demonstrating no heterogeneity (I² = 0%; p = 0.54) 32-34. Therefore, concerns about region-related heterogeneity in this analysis may be reduced. In Southeast Asia, PLA caused by community-acquired, drug-sensitive *Klebsiella pneumoniae* is associated with a lower fatality rate, and our findings are consistent with this observation 35.

In this study, patients with attenuated febrile responses, higher APACHE II scores, ICU admission, or septic shock were associated with increased mortality. An impaired immune response may lead to delayed recognition of infection by clinicians and subsequent delays in antibiotic therapy; accordingly, afebrile patients have been associated with worse outcomes in an infection cohort 36. The APACHE II scoring system was found to be closely correlated with subsequent in-hospital death among ICU patients and has also been validated as a good outcome predictor in infectious diseases 37, 38. In addition, septic shock, characterized by both cellular dysfunction and cardiovascular compromise, contributes to a notably high 30-day mortality of nearly 35% 39. Furthermore, these identified factors—the APACHE II scoring system, septic shock, and ICU admission—primarily function as markers of illness severity rather than independent prognostic determinants.

In accordance with methodological guidelines, adjusted estimates of prognostic factors associated with mortality from each included study were pooled as the primary results in these meta-analyses. When available, unadjusted effect estimates were reported separately in sensitivity analyses. For example, the sensitivity analysis for fever (Table S6) revealed a discrepancy between adjusted and unadjusted estimates: the pooled result based on unadjusted estimates was statistically significant (OR 0.29, 95% CI 0.13-0.68), whereas the pooled result based on adjusted estimates was not statistically significant (aOR 0.53, 95% CI 0.12-2.29). Discrepancies between adjusted and unadjusted estimates probably indicate the presence of confounding, as prognostic factors are often correlated with other predictors. Therefore, adjusted estimates are generally considered more reliable when evaluating the independent prognostic effect 14. In addition, according to the QUIPS tool for assessing risk of bias in prognostic factor studies, inadequate adjustment for important confounders may introduce bias due to confounding. Therefore, in the GRADE assessment of the certainty of evidence, the risk-of-bias domain may be downgraded when studies lack adequate adjustment for key confounders 12, 40.

With respect to radiologic features, gas formation, rather than abscess size or multiple abscesses, emerged as a significant prognostic marker in this systematic review. Gas-forming abscesses result from mixed acid fermentation within the abscess cavity, catalyzed by formic hydrogenlyase. Gas formation in liver abscess has been reported to be associated with poorly controlled DM, which appears to provide a favorable environment for the growth of gas-forming microorganisms and is generally linked to a remarkably high mortality rate. Gas formation usually implies sufficient liquefaction of the abscess for drainage, and thus early abscess drainage, in addition to antibiotic administration, remains the primary principle of infection control 41, 42.

In our study, bacteremia was associated with increased mortality. This association likely reflects greater systemic disease severity rather than a direct causal effect of bloodstream infection per se 43. Additionally, *Klebsiella pneumoniae* infection was inversely associated with mortality, whereas *Escherichia coli* and anaerobic infections were associated with increased mortality in this investigation. These pathogen-specific findings should be interpreted with caution. Differences in underlying host factors, abscess etiology (e.g., biliary versus cryptogenic origin), healthcare exposure, regional practice patterns, and detection methods may confound these associations 44, 45. The extremely large effect estimate observed for anaerobic infection was derived from a limited number of studies and may reflect sparse data bias or residual confounding. Infection with multidrug-resistant organisms was also associated with higher mortality, which may be related to delays in effective antimicrobial therapy and greater underlying comorbidity 46. Overall, microbiological factors appear to be associated with mortality; however, whether they represent independent causal determinants remains uncertain.

In our investigation, percutaneous drainage was associated with a lower mortality rate. Apart from antibiotic administration, drainage was the primary effective therapeutic intervention for PLA 47. The published meta-analyses of randomized controlled trials documented a significantly higher treatment success rate with percutaneous catheter drainage compared with percutaneous needle aspiration, particularly for large abscesses, but no difference in mortality 3, 48. However, in our systematic review, only one of the included studies specified pigtail drainage as the percutaneous drainage modality 49, whereas the other three studies did not clarify the type of percutaneous drainage used. Consequently, percutaneous drainage appears to confer survival benefits based on this meta-analysis of real-world evidence, although the true treatment effect of each specific drainage technique remains uncertain. Additionally, in patients with infected hepatic necrosis due to hepatic artery thrombosis, surgical intervention or liver transplantation may be required, and percutaneous drainage alone may not be sufficient 19, 50. Huang et al. also emphasized that surgical drainage should be regarded as a complementary rather than competing approach, particularly patients in with unsuccessful percutaneous drainage or those requiring operative management of underlying conditions, including multiple large abscesses, steroid use, or ascites 51. Therefore, early source control and pathogen identification through appropriate percutaneous drainage may play an important role in the management of many patients with PLA, although treatment should be individualized according to the underlying etiology and clinical context.

This systematic review has several limitations. First, as these meta-analyses focused on prognostic factors for short-term mortality, further investigation of predictors of long-term prognosis may be necessary. Second, many significant factors were described in this research, but the potential inflation of type I error should be taken into consideration. Third, some factors were recognized as having low to very low certainty of evidence, regardless of whether they were significant or not. The presence of substantial heterogeneity, moderate-to-high risk of bias, and incomplete adjustment for key confounders across studies may compromise the reliability of pooled results for several factors. Because the adjusted estimates were derived from multivariable models with different covariate selections across studies, residual confounding cannot be completely excluded. Fourth, PLA represents a heterogeneous disease with diverse etiologies, including biliary obstruction, hepatic artery injury/ischemia, hematogenous dissemination, and cryptogenic infection. Because most included studies did not stratify outcomes according to detailed etiological categories—particularly hepatic artery injury/ischemia—our pooled analyses may reflect averaged associations across heterogeneous subgroups. This limitation may reduce the ability to identify etiology-specific prognostic determinants and should be considered when interpreting the results. Given the above limitations, future high-quality prospective studies with adequate adjustment for key confounders and detailed etiological classification of PLA are warranted to clarify the prognostic impact of different etiologies, strengthen the evidence base, identify etiology-specific prognostic factors, guide individualized interventions, and improve patient outcomes.

## Conclusion

In this prognostic systematic review and meta-analysis of observational studies, multiple demographic, clinical, laboratory, radiographic, microbiological, and treatment-related factors were associated with short-term mortality in PLA. With high to moderate certainty of evidence, older age, female sex, malignancy, chronic kidney disease, high APACHE II score, septic shock, gas-forming abscess, thrombocytopenia, hyperbilirubinemia, elevated ALT, impaired renal function, bacteremia, *Escherichia coli* infection, multidrug-resistant organism infection, metastatic infection, pneumonia, and ICU admission were consistently associated with increased mortality. In contrast, *Klebsiella pneumoniae* infection and percutaneous drainage were associated with lower mortality. Additional factors — including anemia, anaerobic infection, hypoalbuminemia, and absence of fever — were suggestively associated with increased mortality, although the certainty of evidence was low to very low. These findings should be interpreted as prognostic associations rather than causal relationships, as most included studies were observational and varied in their adjustment for confounding. Several identified factors—particularly disease severity markers and ICU admission—likely reflect underlying disease severity rather than independent, modifiable determinants. Future high-quality prospective studies with standardized adjustment for key confounders are warranted to clarify independent etiology-specific prognostic factors and identify actionable targets for improving clinical outcomes in PLA.

## Supplementary Material

Supplementary figures and tables.

## Figures and Tables

**Table 1:** Characteristics of the included studies

Author, year	Journal	Study design	Country	Population	Sample size	Age (mean or median)	Mortality outcome	Mortality rate	Significant adjusted factors identified
Lee 1991 52	World J Surg	cohort study	Taiwan	PLA	73	50	in-hospital mortality	19%	leukocytosis, albumin, pleural effusion
Mischinger 1994 53	World J Surg	cohort study	Austria	PLA	46	51	in-hospital mortality	17%	serum WBC, hemoglobin, malignancy, APACHE II
Chu 1996 54	Arch Surg	cohort study	China	PLA	83	72	in-hospital mortality	18%	malignancy, hyperbilirubinemia, and elevated APTT
Barakate 1999 55	Aust NZ J Surg	cohort study	Australia	PLA	98	54	in-hospital mortality	8%	NA
Lee 2001 56	Dig Surg	cohort study	Taiwan	PLA	133	53	in-hospital mortality	6%	sepsis
Molle 2001 4	Gut	cohort study	Denmark	PLA	665	NA	30-day mortality	28%	liver cirrhosis, CCI, age, sex
Pérez 2001 57	Am J Surg	cohort study	Spain	PLA	133	60	30-day or in-hospital mortality	14%	biliary origin, shock, multiple abscess, hemoglobin, BUN
Wong 2002 6	J Gastroenterol Hepatol	cohort study	China	PLA	80	63	in-hospital mortality	6%	malignancy
Ng 2002 58	Aliment Pharmacol Ther	cohort study	China	PLA	66	64	in-hospital mortality	3%	NA
Chen 2005a 59*	Wien Klin Wochenschr	cohort study	Taiwan	*E. coli* PLA	72	66	in-hospital mortality	26%	malignancy, hypoalbuminemia, multiple abscess
Chen 2005b 60*	Swiss Med Wkly	cohort study	Taiwan	PLA	86	60	in-hospital mortality	10%	NA
Jepsen 2005 61	Aliment Pharmacol Ther	cohort study	Denmark	PLA	1,448	64	30-day mortality	19%	sex
Chen 2006 62	Swiss Med Wkly	cohort study	Taiwan	PLA	225	60	in-hospital mortality	10%	metastatic infection
Hsieh 2006 63	Dig Liver Dis	cohort study	Taiwan	PLA	314	57	in-hospital mortality	8%	APACHE II score, primary liver cancer history
Chen 2007 64	Am J Med Sci	cohort study	Taiwan	*E. coli* and* K. pneumoniae* PLA	202	58	in-hospital mortality or 30-day mortality	10%	APACHE II score, right-lobar abscess involvement, malignancy, uremia, MDRO
Ruiz-Hernandez 2007 65	Eur J Gastroenterol Hepatol	cohort study	Spain	PLA	84	64	in-hospital mortality	19%	septic shock
Thomsen 2007 7	Clin Infect Dis	case-control study	Denmark	PLA	1,442	64	30-day post-discharge mortality	19%	NA
Chen 2008a 66*	Neth J Med	cohort study	Taiwan	PLA	253	56	in-hospital mortality	9%	gas-forming liver abscess, MDRO, anaerobic infection, BUN > 7.86 mmol/l, APACHE II score ≥ 15
Chen 2008b 67*	Crit Care Med	cohort study	Taiwan	PLA with ICU admission	72	58	in-hospital mortality	28%	acute respiratory failure, APACHE II > 16
Lee 2008 49	Clin Infect Dis	cohort study	Taiwan	*K. pneumoniae* PLA	110	62	in-hospital mortality	10%	APACHE II score, metastatic infection, septic shock, acute respiratory failure, gas formation on imaging, pigtail drainage
Ng 2008 68	Surg Pract	cohort study	China	PLA	143	68	in-hospital mortality	13%	size of abscess, BUN, APTT
Tsai 2008 33	Emerg Infect Dis	cohort study	Taiwan	PLA	29,703	59	in-hospital mortality	11%	age, DM, abscess drainage, biliary procedure, peptic ulcer, urinary tract infection, renal disease, hypertension, cholelithiasis, hepatobiliary malignancy, other malignancy, pneumonia, active viral hepatitis, heart disease
Chen 2009a 32*	Age Ageing	cohort study	Taiwan	PLA in elderly patients	339	59	in-hospital mortality	10%	age, APACHE II score, *K. pneumonia* infection
Chen 2009b 42*	Am J Surg	cohort study	Taiwan	PLA	298	57	in-hospital mortality	10%	APACHE II score at admission, SAPS II score at admission, gas-forming liver abscess, anaerobic infection
Chou 2009 34	Gastroenterol J Taiwan	cohort study	Taiwan	PLA	357	NA	in-hospital mortality	9%	age, albumin
Foo 2010 69	Am J Gastroenterol	cohort study	Taiwan	PLA	377	60	in-hospital mortality	6%	gas forming abscess, creatinine > 1.3mg/dl
Kuo 2010 70	Gastroenterol J Taiwan	cohort study	Taiwan	PLA	120	NA	in-hospital mortality	14%	hypoalbuminemia, renal impairment
Lou 2010 71	Gastroenterol J Taiwan	cohort study	Taiwan	PLA with ICU admission	35	64	ICU mortality	34%	APACHE II score, abscess size
Meddings 2010 43	Am J Gastroenterol	cohort study	USA	PLA	17,787	NA	in-hospital mortality	6%	age, health insurance, hospital characteristics, Elixhauser comorbidity score, cholecystectomy, bacteremia, bacteria classified elsewhere, liver aspiration
Chen 2011 72	J Gastrointest Surg	cohort study	Taiwan	PLA with underlying malignancy	85	66	in-hospital mortality	33%	APACHE II score, multiloculated abscess, polymicrobial infection
Kang 2011 1	J Chin Med Assoc	cohort study	Taiwan	PLA	2,319	60	in-hospital mortality	2%	nephropathy, gastroenterological cancers, acute low respiratory conditions
Law 2011 73	Eur J Gastroenterol Hepatol	cohort study	China	PLA	319	66	in-hospital mortality	16%	malignancy, hypoalbuminemia, DIC, and acute coronary syndrome
Law 2012 10	World J Gastroenterol	cohort study	China	PLA	318	66	in-hospital mortality	16%	malignancy, hypoalbuminemia, DIC, acute coronary syndrome
Tian 2012 9	Clin Microbiol Infect	cohort study	China	PLA	357	60	in-hospital mortality	7%	uremia, gas-forming abscess, MDRO, stay in ICU
Kuo 2013 8	Am J Emerg Med	cohort study	Taiwan	PLA	431	57	in-hospital mortality	15%	higher MEDS scores on admission, malignancy, multiple abscesses, anaerobic infection, TSB > 1.3 mg/dL, serum creatinine > 1.3 mg/dL
Law 2013 74	Int J Infect Dis	cohort study	China	PLA	319	66	in-hospital mortality	16%	age, active hepatic malignancy, serum albumin < 28g/dl, TSB > 30umol/l, DIC
Law 2014 75	Eur J Gastroenterol Hepatol	cohort study	China	PLA	359	66	in-hospital mortality	13%	active hepatic malignancy, DIC, CRP ratio > 0.5 by week 3
Yoon 2014 76	Scand J Infect Dis	cohort study	Korea	*K. pneumoniae* PLA	161	61	in-hospital mortality	2%	APACHE II score
Chen 2014 77	BMC Gastroenterology	cohort study	Taiwan	PLA	134	59	in-hospital mortality	4%	NA
Hong 2014 78	PLoS One	cohort study	Taiwan	PLA in ESRD	447	NA	long-term mortality	NA	DM, chronic liver disease
Li 2015 79	Chin J Crit Care Med	cohort study	China	PLA	272	61	in-hospital mortality	5%	septic shock, malignancy
Czerwonko 2016 80	HPB	cohort study	Argentina	PLA	142	62	in-hospital mortality	8%	TSB >5 mg/dl, bilobular involvement
Shelat 2016 81	Hepatobiliary Pancreat Dis Int	cohort study	Singapore	culture-negative PLA	264	59	in-hospital or 30-day mortality	14%	age, fever, INR, BUN
Sohn 2016 82	Korean J Gastroenterol	cohort study	Korea	PLA	231	64	in-hospital mortality	7%	recurrence, anemia, hyperbilirubinemia, thrombocytopenia
Mucke 2017 83	BMC Infect Dis	cohort study	Germany	PLA	86	62	in-hospital mortality	16%	malignancy, TSB
Bettinger 2018 84	Aliment Pharmacol Ther	cohort study	Germany	PLA	181	NA	in-hospital mortality	22%	Intrahepatic abscess expansion (solitary vs multifocal), bile duct compression, intensive care treatment, treatment with proton pump inhibitors, CCI
Chen 2018 85	J Int Med Res	cohort study	China	PLA	178	55	28-day mortality	4%	NA
Sharma 2018 86	Mayo Clin Proc Innov Qual Outcomes	cohort study	USA	PLA	72	63	3-month mortality	17%	biliary disease, liver disease, malignancy, immunosuppression, cardiovascular disease
Park 2019 87	Eur J Trauma Emerg Surg	cohort study	Korea	PLA	102	65	in-hospital mortality	10%	neutrophil-to-lymphocyte ratio
Xu 2019a 88*	Front Endocrinol	cohort study	China	PLA	240	68	in-hospital mortality	9%	platelet, GNRI < 90, low T3 syndrome
Xu 2019b 89*	BMC Geriatr	cohort study	China	PLA	240	68	in-hospital mortality	9%	platelet, GNRI < 90, PT > 14.8s
Dai 2020 16	preprint	cohort study	China	PLA	240	68	in-hospital mortality	9%	albumin, PT > 14.8s, AST/ALT ratio
Lee 2020 90	Abdom Radiol	cohort study	Korea	PLA	219	61	in-hospital mortality	4%	DM, multiple abscess, internal gas bubble, metastatic infection, septic shock
Ruiz-Hernández 2020 5	Irish J Med Sci	cohort study	Spain	PLA	193	67	in-hospital mortality	16%	*E. coli* PLA
Du 2020 91	BMC Infect Dis	cohort study	China	PLA, excluding malignancy	227	56	6-month mortality	11%	DM
Yoo 2021 2	Liver Int	cohort study	Korea	PLA	30,690	65	in-hospital mortality	10%	sex, age, DM, cancer, CKD, ICU admission
Faridi 2021 92	Int Surg J	cohort study	India	ruptured PLA	40	NA	in-hospital or 30-day mortality	28%	left lobe abscess, shock at presentation, time of presentation, APACHE II score
Lee 2021a 93*	BMC Infect Dis	cohort study	Korea	PLA	648	66	in-hospital mortality	2%	maximal abscess diameter, AKI at admission
Lee 2021b 94*	J Clin Med	cohort study	Taiwan	PLA	324	58	30-day mortality	1%	APACHE II score, concomitant infections
Losie 2021 95	BMC Infect Dis	cohort study	Canada	PLA	136	61	30-day mortality	7%	polymicrobial bacteremia, no drainage performed, history of congestive heart failure, history of liver disease, total bilirubin
Yu 2021 96	Saudi J Gastroenterol	cohort study	China	PLA	239	64	in-hospital mortality	4%	malignancy
Große 2021 97	Sci Rep	cohort study	Germany	PLA	133	65	1-year mortality	9%	enterococcal infection
Chan 2022 98	Malays J Med Sci.	cohort study	Singapore	PLA	213	62	in-hospital mortality	13%	age
Myeong 2022 99	J. Infect. Public Health	cohort study	Korea	PLA	30,690	65	in-hospital mortality	10%	sex, age, antibiotics, DM, colon cancer, other cancer, CKD, endophthalmitis, ICU admission
Park 2022 46	Journal of Clinical Medicine	cohort study	Korea	PLA	833	62	in-hospital mortality	4%	inadequate antibiotics, use of inotropic agents, ICU admission
Wu 2022 100	Chin J Crit Care Med	cohort study	China	PLA with sepsis	120	65	in-hospital mortality	9%	malignancy, liver failure, septic shock
Meister 2022 101	Langenbecks Arch Surg	cohort study	Australia	cryptogenic PLA	98	56	in-hospital mortality	18%	NA
Rossi 2022 102	Infection	cohort study	France	PLA	302	62	3-month mortality	11%	CCI, portal thrombosis, MDRO, drainage
Jiménez-Romero 2023 103	Clin Transplant	cohort study	Spain	PLA following liver transplantation	289	NA	in-hospital mortality	10%	Liver transplantation
Li 2023 104	Front Surg	cohort study	China	PLA	458	53	in-hospital mortality	3%	platelet, hemoglobin, albumin, TSB, creatinine, ARDS, presence of gas
Liu 2023 105	Kaohsiung J Med Sci	cohort study	China	PLA	48	58	In-hospital mortality	15%	NA

*This asterisk symbol denotes two different publications that share the same author surname and publication year; in this article, they are labeled with “a” and “b” following the author surname and year to distinguish them. PLA: Pyogenic liver abscess; ESRD: end-stage renal disease; CNS: central nervous system; WBC: white blood cell count; APACHE II: Acute Physiology and Chronic Health Evaluation II; NA: not applicable; CCI: Charlson Comorbidity Index; SAPS II: Simplified Acute Physiology Score II; MDRO: multidrug-resistant organism; BUN: blood urea nitrogen; TSB: total serum bilirubin; GNRI: Geriatric Nutritional Risk Index;* E. coli*: *Escherichia coli*; *K. pneumoniae*: *Klebsiella pneumoniae*; DM: diabetes mellitus; AST: aspartate aminotransferase; ALT: alanine aminotransferase; CKD: chronic kidney disease; ICU: intensive care unit; APTT: activated partial thromboplastin time; ARDS: acute respiratory distress syndrome; DIC: disseminated intravascular coagulation; MEDS: Mortality in Emergency Department Sepsis score; AKI: acute kidney injury.

**Table 2:** Summary of pooled estimates of all factors for short-term mortality among patients with pyogenic liver abscess

Factor	Number of studies	Sample size	aOR [95% CI], *p* value (Inverse variance method and random-effects model)	Heterogeneity (I^2^, *p* value)	Egger's test^**^ (*p* value)
Age (older vs younger)	9	3,048	2.33 [1.29, 4.19], *p* = 0.005^*^	77%, *p* < 0.001	NA
Age (per 1-year increase)	4	30,559	1.02 [1.01, 1.04], *p* = 0.01^*^	27%, *p* = 0.25	NA
Female sex	6	80,403	1.18 [1.04, 1.33], *p* = 0.01^*^	66%, *p* = 0.01	NA
Malignancy	16	65,972	5.63 [3.39, 9.36], *p* < 0.00001^*^	81%, *p* < 0.00001	0.0064
Chronic kidney disease	8	64,077	2.41 [1.42, 4.07], *p* = 0.001^*^	93%, *p* < 0.00001	NA
Diabetes mellitus	6	62,349	1.06 [0.83, 1.36],* p* = 0.64	80%,* p* = 0.0001	NA
Liver cirrhosis	2	31,355	1.95 [0.45, 8.49], *p* = 0.37	89%,* p* = 0.003	NA
Hypertension	2	30,027	0.94 [0.17, 5.23], *p* = 0.94	86%, *p* = 0.007	NA
Fever	3	880	0.29 [0.13, 0.68], *p* = 0.004^*^	0%, *p* = 0.65	NA
Higher APACHE II score	4	1,026	12.42 [5.85, 26.37], *p* < 0.00001^*^	0%, *p* = 0.88	NA
APACHE II score (per 1-point increase)	4	696	1.34 [1.21, 1.48], *p* < 0.00001^*^	0%, *p* = 0.64	NA
Septic shock	7	2,046	9.14 [4.54, 18.42],* p* < 0.00001^*^	0%, *p* = 0.68	NA
Jaundice	2	734	1.08 [0.01, 137.40], *p* = 0.98	66%, *p* = 0.09	NA
Altered mental status	2	1,025	0.85 [0.04, 18.51], *p* = 0.92	46%, *p* = 0.17	NA
Biliary origin	5	1,202	1.57 [0.66, 3.73], *p* = 0.31	46%, *p* = 0.12	NA
Abscess size (per 1-cm increase)	3	280	1.64 [0.77, 3.51], *p* = 0.20	97%, *p* < 0.00001	NA
Multiple abscesses	8	2,044	2.95 [0.89, 9.78], *p* = 0.08	80%, *p* < 0.00001	NA
Gas-forming abscess	5	1,262	10.16 [4.01, 25.71], *p* < 0.00001^*^	15%, *p* = 0.32	NA
Serum WBC count (per 10⁹/L increase)	2	245	1.02 [0.99, 1.05], *p* = 0.21	43%, *p* = 0.18	NA
Leukocytosis	4	1,218	1.87 [0.30, 11.68], *p* = 0.50	77%, *p* = 0.004	NA
Anemia	5	1,706	4.33 [1.05, 17.91], *p* = 0.04^*^	68%, *p* = 0.01	NA
Thrombocytopenia	3	929	4.18 [2.05, 8.50], *p* < 0.0001^*^	0%, *p* = 0.51	NA
Hypoalbuminemia	8	2,186	4.12 [2.60, 6.53], *p* < 0.00001^*^	0%, *p* = 0.73	NA
Hyperbilirubinemia	6	1,752	8.36 [2.35, 29.72], *p* = 0.001^*^	0%, *p* = 0.43	NA
Elevated ALT	2	1,025	5.40 [1.32, 22.19], *p* = 0.02^*^	0%, *p* = 0.77	NA
Azotemia	4	1,027	5.12 [0.84, 31.24], *p* = 0.08	80%, *p* = 0.002	NA
Impaired renal function	4	1,207	6.42 [1.61, 25.59], *p* = 0.008^*^	76%, *p* = 0.006	NA
Bacteremia	5	18,981	3.26 [1.53, 6.94], *p* = 0.002^*^	37%, *p* = 0.18	NA
*Klebsiella pneumoniae* infection	3	30,399	0.29 [0.16, 0.54], *p* < 0.0001^*^	0%,* p* = 0.54	NA
*Escherichia coli* infection	3	772	2.84 [1.30, 6.21], *p* = 0.009^*^	0%, *p* = 0.90	NA
Anaerobic infection	2	729	63.84 [21.10, 193.18], *p* < 0.00001^*^	0%, *p* = 0.43	NA
Polymicrobial infection	4	1,043	2.09 [0.73, 5.97], *p* = 0.17	47%, *p* = 0.13	NA
Multidrug-resistant organism	3	812	8.43 [2.90, 24.53], *p* < 0.0001^*^	0%,* p* = 0.41	NA
Pneumonia	2	29,823	1.52 [1.33, 1.72], *p* < 0.00001^*^	0%, *p* = 0.96	NA
ICU admission	5	32,380	5.12 [3.84, 6.83], *p* < 0.00001^*^	10%, *p* = 0.35	NA
Metastatic infection	4	878	5.34 [2.32, 12.32], *p* < 0.0001^*^	0%, *p* = 0.46	NA
Ascites	2	497	0.84 [0.06, 11.07], *p* = 0.89	54%,* p* = 0.14	NA
Percutaneous drainage	4	47,736	0.48 [0.37, 0.63], *p* < 0.00001^*^	76%, *p* = 0.007	NA
Surgical drainage	2	18,105	0.92 [0.74, 1.15], *p* = 0.46	0%, *p* = 0.32	NA

^*^ Significance with p value less than 0.05 ^**^ Egger's test was performed only for meta-analyses including at least 10 studies; otherwise, it was not conducted because of limited statistical power. CI: confidence interval; NA: Not Applicable; APACHE II: Acute Physiology and Chronic Health Evaluation II; WBC: white blood cell; ALT: alanine aminotransferase; ICU: intensive care unit.

**Table 3:** GRADE summary of findings on prognostic factors for short-term mortality among patients with liver abscess.

Factors	Study number^1^	Patient number	Risk of bias^2^	Inconsistency^3^	Indirectness^4^	Imprecision^5^	Large effect^6^	Other considerations^7^	Baseline risk^8^	Relative effect (aOR) with 95% CI	Absolute effect with 95% CI^9^	GRADE Certainty^10^
Age(older vs younger)	9	3,048	0	0	0	0	+1	0	8.5%	2.33 [1.29, 4.19]	87 more per 1,000(from 22 more to 195 more)	⨁⨁⨁⨁High
Age (per 1-year increase)	4	30,559	0	0	0	0	0	0	8.5%	1.02 [1.01, 1.04]	2 more per 1,000(from 1 more to 3 more)	⨁⨁⨁⨁High
Female sex	6	80,403	0	0	0	-1^f^	0	0	9.0%	1.18 [1.04, 1.33]	15 more per 1,000(from 3 more to 26 more)	⨁⨁⨁Moderate
Malignancy	16	65,972	0	0	0	-2^g^	+2	Publication bias (-1)^i^	9.1%	5.63 [3.39, 9.36]	269 more per 1,000(from 162 more to 393 more)	⨁⨁⨁Moderate
Chronic kidney disease	8	64,077	0	0	0	0	+1	Dose response (+1)^j^	5.8%	2.41 [1.42, 4.07]	71 more per 1,000(from 22 more to 142 more)	⨁⨁⨁⨁High
Diabetes mellitus	6	62,349	0	-1^c, e^	0	-2^h^	0	0	13.2%	1.06 [0.83, 1.36]	7 more per 1,000(from 20 fewer to 39 more)	⨁ Very low
Liver cirrhosis	2	31,355	0	-1^c, d^	0	-2^h^	0	0	26.9%	1.95 [0.45, 8.49]	149 more per 1,000(from 127 fewer to 489 more)	⨁ Very low
Hypertension	2	30,027	0	-1^c, d, e^	0	-2^h^	0	0	10.9%	0.94 [0.17, 5.23]	6 fewer per 1,000(from 88 fewer to 281 more)	⨁ Very low
Fever	3	880	-1^a^	0	0	-2^g^	+1	0	25.3%	0.29 [0.13, 0.68]	164 fewer per 1,000(from 211 fewer to 66 fewer)	⨁⨁ Low
High APACHE II score	4	1,026	0	0	0	-2^g^	+2	Dose response (+1)^j^	2.8%	12.42 [5.85, 26.27]	234 more per 1,000(from 115 more to 401 more)	⨁⨁⨁⨁High
APACHE II score (per 1-point increase)	4	696	0	0	0	0	0	Publication bias (-1)^i^	2.8%	1.34 [1.21, 1.48]	9 more per 1,000(from 6 more to 13 more)	⨁⨁⨁Moderate
Septic shock	7	2,046	0	0	0	-2^g^	+2	0	4.9%	9.14 [4.54, 18.42]	270 more per 1,000(from 140 more to 436 more)	⨁⨁⨁⨁High
Jaundice	2	734	-1^a^	-1^c, e^	0	-2^h^	0	0	5.6%	1.08 [0.01, 137.40]	4 more per 1,000(from 55 fewer to 834 more)	⨁ Very low
Altered mental status	2	1,025	-1^a^	-1^c, e^	0	-2^h^	0	0	3.6%	0.85 [0.04, 18.51]	5 fewer per 1,000(from 35 fewer to 373 more)	⨁ Very low
Biliary origin	5	1,202	-1^a^	0	0	-2^h^	0	0	7.3%	1.57 [0.66, 3.73]	37 more per 1,000(from 24 fewer to 154 more)	⨁ Very low
Abscess size (per 1-cm increase)	3	280	0	0	0	-2^h^	0	0	14.3%	1.64 [0.77, 3.51]	72 more per 1,000(from 29 fewer to 226 more)	⨁⨁ Low
Multiple abscesses	8	2,044	0	0	0	-1^f^	0	0	7.3%	2.95 [0.89, 9.78]	115 more per 1,000(from 7 fewer to 362 more)	⨁⨁⨁Moderate
Gas-forming abscess	5	1,262	0	0	0	-2^g^	+1	0	7.1%	10.16 [4.01, 25.71]	366 more per 1,000(from 164 more to 592 more)	⨁⨁⨁Moderate
Serum WBC count (per 10⁹/L increase)	2	245	-1^a, b^	0	0	-1^f^	0	0	7.5%	1.02 [0.99, 1.05]	1 more per 1,000(from 1 fewer to 3 more)	⨁⨁ Low
Leukocytosis	4	1,218	-1^a^	-1^c, d, e^	0	-2^h^	0	0	7.5%	1.87 [0.30, 11.68]	57 more per 1,000(from 51 fewer to 412 more)	⨁ Very low
Anemia	5	1,746	-1^a^	-1^c, d, e^	0	-2^g^	+1	0	4.9%	4.33 [1.05, 17.91]	133 more per 1,000(from 2 more to 430 more)	⨁ Very low
Thrombocytopenia	3	929	0	0	0	0	+1	0	1.1%	4.18 [2.05, 8.50]	33 more per 1,000(from 11 more to 75 more)	⨁⨁⨁⨁High
Hypoalbuminemia	8	2,186	0	0	0	-2^g^	+1	Publication bias (-1)^i^	5.9%	4.12 [2.60, 6.53]	145 more per 1,000(from 81 more to 230 more)	⨁⨁ Low
Hyperbilirubinemia	6	1,752	0	0	0	-2^g^	0	Dose response (+1)^j^	3.8%	8.36 [2.35, 29.72]	211 more per 1,000(from 47 more to 502 more)	⨁⨁⨁Moderate
Elevated ALT	2	1,025	-1^a^	0	0	-2^g^	+2	0	3.6%	5.40 [1.32, 22.19]	132 more per 1,000(from 11 more to 418 more)	⨁⨁⨁Moderate
Azotemia	4	1,027	-1^a, b^	-1^c, d, e^	0	-2^h^	0	0	6.5%	5.12 [0.84, 31.24]	198 more per 1,000(from 10 fewer to 621 more)	⨁ Very low
Impaired renal function	4	1,247	0	0	0	-2^g^	+1	0	7.0%	6.42 [1.61, 25.59]	256 more per 1,000(from 38 more to 588 more)	⨁⨁⨁Moderate
Bacteremia	5	18,981	0	0	0	0	+1	0	2.8%	3.26 [1.53, 6.94]	58 more per 1,000(from 14 more to 139 more)	⨁⨁⨁⨁High
*Klebsiella pneumoniae* infection	3	30,399	0	0	0	0	+1	0	13.5%	0.29 [0.16, 0.54]	91 fewer per 1,000(from 110 fewer to 57 fewer)	⨁⨁⨁⨁High
*Escherichia coli* infection	3	772	0	0	0	0	+1	0	4.1%	2.84 [1.30, 6.21]	67 more per 1,000(from 12 more to 168 more)	⨁⨁⨁⨁High
Anaerobic infection	2	729	0	0	0	-2^g^	0	0	9.7%	63.84 [21.10, 193.18]	776 more per 1,000(from 597 more to 857 more)	⨁⨁Low
Polymicrobial infection	4	1,043	-1^a^	0	0	-2^h^	0	0	6.5%	2.09 [0.73, 5.97]	62 more per 1,000(from 17 fewer to 229 more)	⨁ Very low
Multidrug-resistant organism	3	812	0	0	0	-2^g^	+1	0	4.8%	8.43 [2.90, 24.53]	250 more per 1,000(from 79 more to 505 more)	⨁⨁⨁Moderate
Pneumonia	2	29,823	0	0	0	-1^f^	0	0	2.9%	1.52 [1.33, 1.72]	15 more per 1,000(from 9 more to 20 more)	⨁⨁⨁Moderate
ICU admission	5	32,380	0	0	0	0	+2	0	5.3%	5.12 [3.84, 6.83]	170 more per 1,000(from 124 more to 224 more)	⨁⨁⨁⨁High
Metastatic infection	4	878	0	0	0	-2^g^	+1	0	6.2%	5.34 [2.32, 12.32]	198 more per 1,000(from 71 more to 385 more)	⨁⨁⨁Moderate
Ascites	2	497	-1^a^	-1^c, e^	0	-2^h^	0	0	5.7%	0.84 [0.06, 11.07]	9 fewer per 1,000(from 53 fewer to 343 more)	⨁ Very low
Percutaneous drainage	4	47,736	0	0	0	0	+1	0	7.5%	0.48 [0.37, 0.63]	37 fewer per 1,000(from 46 fewer to 26 fewer)	⨁⨁⨁⨁High
Surgical drainage	2	18,105	0	0	0	-1^f^	0	0	5.6%	0.92 [0.74, 1.15]	4 fewer per 1,000(from 14 fewer to 8 more)	⨁⨁⨁Moderate

^1^ Study type was all observational studies. ^2^ The risk of bias domain was downgraded by one level if: ^a^ More than 60% of the studies had a high overall risk of bias, or fewer than 40% of the studies had a low overall risk of bias. ^b^ A discrepancy between studies at high risk of bias and those at low risk of bias was observed in the subgroup analysis, if available. ^3^ The inconsistency domain was downgraded by one level if any of the following criteria were met: ^c^ There was an important difference in the point estimates across studies. ^d^ There was limited overlap in their CIs. ^e^ A substantial proportion of point estimates fell on opposite sides of the null effect threshold. ^4^ The indirectness domain was downgraded by one level if there was a considerable mismatch between the PICO elements in the included studies and the target research question. ^5^ For the assessment of the imprecision domain, the minimally important difference (MID) was defined as follows: For categorical variables, the MID for benefit was set at an absolute risk reduction of 10 deaths per 1,000 patients (1.0%), and the MID for harm was set at an absolute risk increase of 10 deaths per 1,000 patients (1.0%). For continuous variables, including laboratory values or age increments per unit, the MID for benefit was set at an absolute risk reduction of 1 death per 1,000 patients (0.1%), and the MID for harm was set at an absolute risk increase of 1 death per 1,000 patients (0.1%). The threshold for a large effect of absolute risk for any variable was defined as 20 times the corresponding minimally important difference (MID). The imprecision domain was downgraded under the following conditions: ^f^ By one level if the CI crossed any MID. ^g^ By two levels if the CI crossed any large effect threshold and the aOR CI ratio was greater than 2.5 for binary outcomes. ^h^ By two levels if the CI crossed both MIDs for benefit and harm.^6^ The large effect domain was assessed using the pooled point estimate of the aOR for relative risk.: The domain was upgraded by one level if the estimated aOR was > 2 or < 0.5. The domain was upgraded by two levels if the estimated aOR was > 5 or < 0.2. No or limited upgrading was applied when substantial downgrading was present in the risk of bias, inconsistency, or imprecision domains. ^7^ Other considerations included the publication bias domain and the dose-response domain: ^i^ The publication bias domain was downgraded by one level if a significant result in Egger's test or an asymmetric funnel plot was observed. ^j^ The dose-response domain was upgraded by one level if a dose-response gradient was identified in subgroup analyses. ^8^ Baseline risks were calculated based on the number of events and total participants in the control groups (i.e., those without the prognostic factor) of studies examining the factor of interest. ^9^ Outcome was short-term mortality, including in-hospital mortality, 28-day mortality, or 30-day mortality. Absolute effects were calculated using baseline risks and relative effects via GRADEpro (https://www.gradepro.org/). ^10^ All certainty ratings began at high certainty because this was a meta-analysis of prognostic factor studies. aOR: adjusted odds ratio; GRADE: grading of recommendations, assessment, development and evaluation; CI: confidence interval; No: number; APACHE II: Acute Physiology and Chronic Health Evaluation II; WBC: white blood cell; ALT: alanine aminotransferase; ICU: intensive care unit.

## Data Availability

The authors stated that all data supporting the findings of this research are provided within the article and its supplementary materials.

## Abbreviations

PLA: pyogenic liver abscess
PRISMA: Preferred Reporting Items for Systematic Reviews and Meta-Analyses
CHARMS-PF: Checklist for Critical Appraisal and Data Extraction for Systematic Reviews of Prognostic Factor Studies
ICTRP: International Clinical Trials Registry Platform
ICU: intensive care unit
QUIPS: Quality in Prognostic Studies
CKD: chronic kidney disease
OR: odds ratio
aOR: adjusted odds ratio
HR: hazard ratio
CI: confidence interval
GRADE: Grading of Recommendations, Assessment, Development and Evaluations
APACHE II: Acute Physiology and Chronic Health Evaluation II
ALT: alanine aminotransferase
