# Supplementary material for: Prognostic Factors for Mortality in Patients with Pyogenic Liver Abscess: A Systematic Review and Meta-Analysis
Source: Int J Med Sci. 2026 Mar 25;23(5):1685–706. doi: 10.7150/ijms.130156 (PMC13133893; doi:10.7150/ijms.130156)
Supplement: Supplementary file 1 — Supplementary figures and tables. [file ijmsv23p1685s1.pdf]

Table S1 PRISMA Checklist

Section and Topic	Item #	Checklist item	Location where item is reported
TITLE			
Title	1	Identify the report as a systematic review.	Title
ABSTRACT			
Abstract	2	See the PRISMA 2020 for Abstracts checklist.	Abstract section
INTRODUCTION			
Rationale	3	Describe the rationale for the review in the context of existing knowledge.	Introduction section
Objectives	4	Provide an explicit statement of the objective(s) or question(s) the review addresses.	Introduction section
METHODS			
Eligibility criteria	5	Specify the inclusion and exclusion criteria for the review and how studies were grouped for the syntheses.	Methods section
Information sources	6	Specify all databases, registers, websites, organisations, reference lists and other sources searched or consulted to identify studies. Specify the date when each source was last searched or consulted.	Methods section
Search strategy	7	Present the full search strategies for all databases, registers and websites, including any filters and limits used.	Methods section

Selection process	8	Specify the methods used to decide whether a study met the inclusion criteria of the review, including how many reviewers screened each record and each report retrieved, whether they worked independently, and if applicable, details of automation tools used in the process.	Methods section
Data collection process	9	Specify the methods used to collect data from reports, including how many reviewers collected data from each report, whether they worked independently, any processes for obtaining or confirming data from study investigators, and if applicable, details of automation tools used in the process.	Methods section
Data items	10a	List and define all outcomes for which data were sought. Specify whether all results that were compatible with each outcome domain in each study were sought (e.g. for all measures, time points, analyses), and if not, the methods used to decide which results to collect.	Methods section
	10b	List and define all other variables for which data were sought (e.g. participant and intervention characteristics, funding sources). Describe any assumptions made about any missing or unclear information.	Methods section
Study risk of bias assessment	11	Specify the methods used to assess risk of bias in the included studies, including details of the tool(s) used, how many reviewers assessed each study and whether they worked independently, and if applicable, details of automation tools used in the process.	Methods section

Effect measures	12	Specify for each outcome the effect measure(s) (e.g. risk ratio, mean difference) used in the synthesis or presentation of results.	Methods section
Synthesis methods	13a	Describe the processes used to decide which studies were eligible for each synthesis (e.g. tabulating the study intervention characteristics and comparing against the planned groups for each synthesis (item #5)).	Methods section
	13b	Describe any methods required to prepare the data for presentation or synthesis, such as handling of missing summary statistics, or data conversions.	Methods section
	13c	Describe any methods used to tabulate or visually display results of individual studies and syntheses.	Methods section
	13d	Describe any methods used to synthesize results and provide a rationale for the choice(s). If meta-analysis was performed, describe the model(s), method(s) to identify the presence and extent of statistical heterogeneity, and software package(s) used.	Methods section
	13e	Describe any methods used to explore possible causes of heterogeneity among study results (e.g. subgroup analysis, meta-regression).	Methods section
	13f	Describe any sensitivity analyses conducted to assess robustness of the synthesized results.	Methods section
Reporting bias assessment	14	Describe any methods used to assess risk of bias due to missing results in a synthesis (arising from reporting biases).	Methods section

Certainty assessment	15	Describe any methods used to assess certainty (or confidence) in the body of evidence for an outcome.	Methods section
RESULTS			
Study selection	16a	Describe the results of the search and selection process, from the number of records identified in the search to the number of studies included in the review, ideally using a flow diagram.	Results section and Figure S1
	16b	Cite studies that might appear to meet the inclusion criteria, but which were excluded, and explain why they were excluded.	Results section and Figure S1
Study characteristics	17	Cite each included study and present its characteristics.	Results section, and Table 1, S3, S4
Risk of bias in studies	18	Present assessments of risk of bias for each included study.	Results section and Figure S2
Results of individual studies	19	For all outcomes, present, for each study: (a) summary statistics for each group (where appropriate) and (b) an effect estimate and its precision (e.g. confidence/credible interval), ideally using structured tables or plots.	Table 1, S3, S4 and Figure 1, 2, S3-S25
Results of syntheses	20a	For each synthesis, briefly summarise the characteristics and risk of bias among contributing studies.	Results section, Figure S2 and Table 2

	20b	Present results of all statistical syntheses conducted. If meta-analysis was done, present for each the summary estimate and its precision (e.g. confidence/credible interval) and measures of statistical heterogeneity. If comparing groups, describe the direction of the effect.	Results section, Figure 1, 2, S3-S25, and Table 2
	20c	Present results of all investigations of possible causes of heterogeneity among study results.	Table S5
	20d	Present results of all sensitivity analyses conducted to assess the robustness of the synthesized results.	Table S6
Reporting biases	21	Present assessments of risk of bias due to missing results (arising from reporting biases) for each synthesis assessed.	Table 2
Certainty of evidence	22	Present assessments of certainty (or confidence) in the body of evidence for each outcome assessed.	Table 3
DISCUSSION			
Discussion	23a	Provide a general interpretation of the results in the context of other evidence.	Discussion section
	23b	Discuss any limitations of the evidence included in the review.	Discussion section
	23c	Discuss any limitations of the review processes used.	Discussion section
	23d	Discuss implications of the results for practice, policy, and future research.	Conclusion section
OTHER INFORMATION			

Registration and protocol	24a	Provide registration information for the review, including register name and registration number, or state that the review was not registered.	Methods section
	24b	Indicate where the review protocol can be accessed, or state that a protocol was not prepared.	Methods section
	24c	Describe and explain any amendments to information provided at registration or in the protocol.	Methods section
Support	25	Describe sources of financial or non-financial support for the review, and the role of the funders or sponsors in the review.	Declarations section
Competing interests	26	Declare any competing interests of review authors.	Declarations section
Availability of data, code and other materials	27	Report which of the following are publicly available and where they can be found: template data collection forms; data extracted from included studies; data used for all analyses; analytic code; any other materials used in the review.	Declarations section

Table S2 Search strategy

Database or registry	Search term or method
PubMed	#1 hepatic abscess[All Fields]<br>#2 liver abscess[All Fields]<br>#3 liver abscess[MeSH Terms]<br>#4 hepatic abscess[MeSH Terms]<br>#5 #1 OR #2 OR #3 OR #4<br>#6 risk factor[All Fields]<br>#7 prognosis [All Fields]<br>#8 prognostic factor[All Fields]<br>#9 predictor[All Fields]<br>#10 #6 OR #7 OR #8 OR #9<br>#11 mortality[All Fields]<br>#12 #5 AND #10 AND #11
Cochrane (Cochrane Database of Systematic Reviews and *Cochrane* Central Register of Controlled Trials)	#1 liver abscess<br>#2 MeSH descriptor: [Liver Abscess] explode all trees<br>#3 hepatic abscess<br>#4 MeSH descriptor: [Risk Factors] explode all trees<br>#5 MeSH descriptor: [Prognosis] explode all trees<br>#6 "prognosis" OR "risk factor" OR "predictor" OR "prognostic"<br>#7 MeSH descriptor: [Mortality] explode all trees<br>#8 Mortality<br>#9 (#1 OR #2 OR #3) AND (#4 OR #5 OR #6) AND (#7 OR #8)
Web of Science	(ALL=(liver abscess) OR ALL=(hepatic abscess)) AND (ALL=(risk factor) OR ALL=(prognosis) OR ALL=(prognostic factor) OR ALL=(predictor)) AND (ALL=(mortality))
Embase	#1 ‘liver abscess’/exp OR ‘liver abscess’ OR ((‘liver’/exp OR liver) AND (‘abscess’/exp OR abscess))<br>#2 ‘hepatic abscess’/exp OR ‘hepatic abscess’ OR (hepatic AND (‘abscess’/exp OR abscess))<br>#3 ‘risk factor’/exp OR ‘risk factor’ OR ((‘risk’/exp OR risk) AND factor)<br>#4 ‘prognostic factor’/exp OR ‘prognostic factor’ OR (prognostic AND factor)

	#5 ‘prognosis’/exp OR prognosis<br>#6 predictor<br>#7 ‘mortality’/exp OR mortality<br>#8 #1 OR #2<br>#9 #3 OR #4 OR #5 OR #6<br>#10 #7 AND #8 AND #9
Europe PMC	(ABSTRACT:"liver abscess" OR ABSTRACT:"hepatic abscess") AND ("risk factor" OR "risk factors" OR “prognosis" OR “prognostic factor" OR "prognostic factors" OR “predictors" OR “predictor") AND ("mortality")
LILACS (via VHL Regional Portal)	("liver abscess" OR "hepatic abscess") AND ("prognosis" OR "risk factor" OR "risk factors" OR "predictor" OR "predictors" OR "prognostic factor" OR "prognostic factors")
Airiti Library	("liver abscess" OR "hepatic abscess" OR "肝膿瘍") AND ("prognosis" OR "risk factor" OR "predictor" OR "predictive" OR "prognostic factor" OR "因子" OR "風險" OR "預測" OR "危險" OR "預後")
Google Scholar	("liver abscess" OR "hepatic abscess") AND ("prognosis" OR "risk factor" OR "risk factors" OR "predictor" OR "predictors" OR "prognostic factor" OR "prognostic factors") AND ("mortality")<br>[The first 200 results]
International Clinical Trials Registry Platform (ICTRP) (WHO)	("liver abscess" OR "hepatic abscess")
ClinicalTrials.gov	Search term:<br>Condition or disease: "liver abscess" OR "hepatic abscess"<br>Study type: All studies<br>Study result: All studies
Meeting abstracts of three international conferences	1. IDWeek meeting [IDWeek is the joint annual meeting of the Infectious Diseases Society of America (IDSA), Society for Healthcare Epidemiology of America (SHEA), the HIV Medicine

	Association (HIVMA), the Pediatric Infectious Diseases Society (PIDS), and the Society of Infectious Diseases Pharmacists (SIDP)]:<br>Browse supplements and IDWeek Abstracts in the *Open Forun Infectious Diseases* journal website (https://academic.oup.com/ofid/supplements).<br><br>2. The annual meeting of the Society for Academic Emergency Medicine:<br>Search the keywords "SAEM annual meeting abstracts" in the *Academic Emergency Medicine* journal website *(*https://onlinelibrary.wiley.com/journal/15532712).<br><br>3. The AASLD (American Association for the Study of Liver Disease) annual meeting<br>Search the keywords "AASLD annual meeting abstract" in the *Hepatology* journal website *(*https://aasldpubs.onlinelibrary.wiley.com).
Citation searching from 6 reviews	Search references of the six reviews[1-6] via Web of Science, Google Scholar or PubMed.
Reference list of included studies	Search references via Web of Science, Google Scholar or PubMed.

Table S3. Other characteristics of the included studies

Author, year	Male	Malignancy	DM	CKD	*Klebsiella* spp.	Sepsis or septic shock	Interventions: PCD / PNA / SD	ICU admission	Hospital stays (mean or median days)
Lee 1991	66%	1%	5%	NA	25%	4%	44% / NA / 45%	NA	NA
Mischinger 1994	65%	9%	NA	NA	3%	NA	41% / NA / 59%	NA	NA
Chu 1996	28%	6%	NA	NA	24% (abscess); 17% (blood)	52%	29% / 24% / 11%	NA	NA
Barakate 1999	61%	20%	6%	NA	10% (abscess); 9% (blood)	NA	71% / 81% / 21%	NA	NA
Lee 2001	62%	2%	29%	7%	66%	18%	82% / 96% / 16%	NA	NA
Molle 2001	55%	NA	NA	NA	NA	NA	NA / NA / NA	NA	NA
Pérez 2001	62%	22%	13%	NA	13%	6%	38% / 9% / 34%	NA	34
Wong 2002	63%	6%	41%	NA	43% (abscess); 35% (blood)	18%	49% / 28% / 0%	3%	NA
Ng 2002	59%	3%	41%	NA	27% (abscess); 25% (blood)	13%	48% / 24% / 1%	NA	35
Chen 2005a	57%	31%	31%	6%	0%	NA	71% / 8% / 1%	NA	19
Chen 2005b	62%	8%	44%	9%	73%	16%	77% / 15% / NA	NA	NA
Jepsen 2005	54%	NA	NA	NA	NA	NA	NA / NA / NA	NA	NA
Chen 2006	64%	12%	49%	2%	68%	4%	84% (all interventions)	NA	19
Hsieh 2006	64%	11%	46%	NA	56%	NA	63% / NA / 11%	NA	NA
Chen 2007	63%	16%	50%	5%	73%	NA	85% / 8% / 1%	NA	NA
Ruiz-Hernandez 2007	67%	14%	26%	NA	17%	18%	61% / 16% / 30%	NA	NA
Thomsen 2007	54%	18%	11%	NA	NA	8%	NA / NA / NA	NA	NA
Chen 2008a	58%	8%	42%	9%	73% (abscess); 55% (blood)	NA	77% / 14% / 1%	NA	21
Chen 2008b	64%	7%	51%	10%	74%	56%	82% (PCD or PNA) / 8% (SD)	100%	31
Lee 2008	54%	7%	61%	5%	100%	25%	88% (PCD or PNA) / NA (SD)	NA	23
Ng 2008	62%	7%	NA	NA	49%	7%	82% / 18% / 4%	NA	30
Tsai 2008	62%	14%	33%	7%	80%	24%	34% (all interventions)	NA	17
Chen 2009a	62%	12%	NA	NA	78%	NA	81% / 7% / 1%	NA	22
Chen 2009b	59%	9%	42%	9%	72%	NA	92% (all interventions)	NA	22
Chou 2009	57%	9%	36%	NA	56%	14%	NA / NA / NA	NA	NA
Foo 2010	68%	12%	48%	7%	73% (abscess)	18%	90% / 1% / 3%	NA	19.
Kuo 2010	57%	20%	50%	NA	58%	13%	67% (PCD or PNA) / NA (SD)	NA	12

Lou 2010	74%	23%	60%	NA	60%	69%	100% (PCD or PNA) / 29% (SD)	100%	NA
Meddings 2010	60%	NA	20%	1%	9%	52%	NA / 53% / 10%	NA	NA
Chen 2011	52%	100%	40%	NA	48%	NA	95% (PCD or PNA) / 11% (SD)	NA	29
Kang 2011	63%	6%	38%	5%	NA	NA	NA / NA / NA	NA	17
Law 2011	57%	13%	28%	NA	44%	29%	71% / 82% / 6%	10%	NA
Law 2012	57%	11%	28%	NA	44%	29%	67% / 82% / 6%	10%	NA
Tian 2012	56%	7%	39%	3%	63%	6%	73% / NA / 12%	NA	27
Kuo 2013	61%	10%	44%	11%	76%	NA	92% / NA / 1%	22%	21
Law 2013	57%	13%	28%	NA	44%	29%	71% / 82% / 6%	10%	NA
Law 2014	57%	NA	28%	NA	50%	27%	83% / NA / 5%	NA	NA
Yoon 2014	62%	9%	27%	3%	100%	10%	86% (all interventions)	NA	23
Chen 2014	29%	31%	64%	38%	71%	NA	NA / NA / NA	NA	NA
Hong 2014	46%	10%	57%	100%	NA	NA	NA / NA / NA	NA	NA
Li 2015	59%	NA	100%	NA	21%	NA	57% (all interventions)	NA	18
Czerwonko 2016	64%	NA	22%	NA	14%	8%	83% / NA / 3%	NA	NA
Shelat 2016	61%	6%	33%	7%	0%	NA	35% (PCD or PNA) / 0% (SD)	NA	16
Sohn 2016	60%	15%	23%	NA	38%	NA	62% / 9% / 3%	NA	NA
Mucke 2017	64%	35%	24%	NA	17%	NA	91% (PCD or PNA) / 9% (SD)	28%	20
Bettinger 2018	67%	55%	NA	NA	10% (abscess); 12% (blood)	NA	67% (PCD or PNA) / NA (SD)	25%	27
Chen 2018	64%	8%	35%	2%	66%	10%	48% (PCD or PNA) / 10% (SD)	19%	NA
Sharma 2018	64%	24%	32%	1%	NA	NA	43% / 28% / 16%	NA	NA
Park 2019	55%	0%	14%	NA	26%	15%	41% (PCD or PNA) / 0% (SD)	14%	19
Xu 2019a	61%	NA	48%	NA	75%	8%	53% (PCD or PNA) / 2% (SD)	NA	18
Xu 2019b	61%	8%	48%	NA	75%	8%	53% (PCD or PNA) / 2% (SD)	NA	18
Dai 2020	61%	NA	NA	NA	75%	8%	53% (PCD or PNA) / 2% (SD)	NA	18
Lee 2020	69%	12%	34%	7%	61%	16%	94% (PCD or PNA) / 6% (SD)	NA	15
Ruiz-Hernández 2020	58%	14%	36%	10%	15%	70%	73% (PCD or PNA) / 23% (SD)	NA	29
Du 2020	59%	0%	27%	NA	43% (abscess); 13% (blood)	NA	60% (PCD or PNA) / 12% (SD)	NA	14
Yoo 2021	61%	26%	37%	3%	NA	NA	NA / NA / NA	17%	20
Faridi 2021	93%	NA	33%	NA	NA	35%	NA / NA / 83%	NA	13
Lee 2021a	63%	15%	28%	NA	36%	NA	56% / 2% / 0%	NA	19
Lee 2021b	69%	11%	45%	4%	42%	11%	56% / NA / 5%	34%	18
Losie 2021	63%	9%	24%	3%	25%	NA	NA / 74% / NA	NA	NA
Yu 2021	56%	8%	52%	NA	21%	28%	NA / NA / NA	NA	18
Große 2021	59%	39%	NA	8%	NA	NA	87% (PCD or PNA) / NA (SD)	29%	NA

Chan 2022	62%	NA	41%	14%	82% (abscess); 70% (blood)	8%	57% / NA / NA	NA	NA
Myeong 2022	61%	25%	37%	3%	NA	NA	NA / NA / NA	17%	20
Park 2022	64%	16%	26%	NA	73% (abscess); 71% (blood)	NA	69% (PCD or PNA) / NA (SD)	NA	NA
Wu 2022	61%	7%	49%	NA	58%	100%	72% (all interventions)	18%	22
Meister 2022	55%	15%	17%	NA	NA	NA	72% (PCD or PNA) / 38% (SD)	NA	40
Rossi 2022	65%	42%	23%	7%	18%	24%	45% / 2% / 10%	NA	NA
Jiménez-Romero 2023	65%	12%	29%	NA	21%	7%	44% (PCD or PNA) / 14% (SD)	11%	19
Li 2023	71%	5%	22%	NA	62%	4%	61% (PCD or PNA) / 7% (SD)	3%	NA
Liu 2023	65%	33%	38%	NA	31%	10%	NA / NA / NA	NA	NA

PCD: percutaneous catheter drainage; PNA: percutaneous needle aspiration; SD: surgical drainage; DM: diabetes mellitus; CKD: chronic kidney disease; ICU: intensive care unit; NA: not available.

Table S4. Adjusted and unadjusted factors reported across the included studies

Author, year	Univariate analysis		Multivariate analysis		
	Unadjusted factors	Number of unadjusted factors	Adjusted factors	Number of adjusted factors	Number of included core confounders*
Lee 1991	WBC, albumin, pleural effusion, ALK-P, bilirubin, AST, bacterial culture, jaundice, abscess location	9	WBC, albumin, pleural effusion, ALK-P	4	0
Mischinger 1994	WBC, Hb, malignancy, APACHE II score, bilirubin, ALK-P, albumin	7	WBC, Hb, malignancy, APACHE II score	4	3
Chu 1996	Female sex, abscess rupture, emergency laparotomy, malignancy, glucose, bilirubin, PT, APTT, treatment without aspiration, treatment without drainage	10	Malignancy, bilirubin, APTT	3	1
Barakate 1999	Malignancy, multi-loculation, failed percutaneous drainage, bilirubin, Hb	5	Nil	0	0
Lee 2001	Abscess size, DM, jaundice, sepsis, sex, age, fever, chills, renal failure, cirrhosis, abscess rupture, abscess number, biliary origin, pleural effusion, gas-forming abscess, albumin, bilirubin, AST, ALK-P, WBC, bacterial culture	21	Abscess size, DM, jaundice, sepsis	4	1
Molle 2001	Cirrhosis, sex, age, CCI	4	Cirrhosis, sex, age, CCI	4	5
Pérez 2001	Biliary origin, shock, abscess number, Hb, BUN, WBC, ALK-P, bilirubin, AST, Cre, albumin, PT, APTT, bacteremia, polymicrobial infection	15	Biliary origin, shock, abscess number, Hb, BUN	5	1
Wong 2002	Multiple abscesses, DM, malignancy	3	Multiple abscesses, DM, malignancy	3	2
Ng 2002	Sex, abscess rupture, emergency laparotomy, malignancy, hyperglycemia, bilirubin, elevated PT, treatment without aspiration or drainage, hospital, year of hospital admission, route of antibiotic administration	11	Nil	0	0
Chen 2005a	Malignancy, pleural effusion, bilobar abscesses, multiple abscesses, abscess size, albumin, bilirubin, prolonged PT, elevated AST, elevated BUN	10	Malignancy, albumin, multiple abscess	3	1
Chen 2005b	Abscess etiology	1	Abscess etiology, age, sex, duration of symptoms	4	2
Jepsen 2005	Year of diagnosis, age, sex	3	Year of diagnosis, age, sex	3	2
Chen 2006	Metastatic infection, sex, age, duration of symptoms	4	Metastatic infection, sex, age, duration of symptoms	4	2
Hsieh 2006	APACHE II score, liver cancer, albumin, bilirubin, BUN, Alk-P, ALT, hyperglycemia	8	APACHE II score, liver cancer, albumin, bilirubin, BUN, Alk-P, ALT	7	4
Chen 2007	*E. coli* group: APACHE II score, right-lobar abscess involvement, multiple abscesses, MDRO, malignancy<br>KP group: uremia, gas-forming abscess, MDRO, and ineffective initial antibiotic treatment	*E. coli* group: 5<br>KP group: 4	*E. coli* group: APACHE II score, right-lobar abscess involvement, malignancy<br>KP group: uremia, MDRO	*E. coli* group: 3<br>KP group: 2	3

Ruiz-Hernandez 2007	Age, coronary artery disease, the absence of fever, sepsis or septic shock, jaundice, higher bilirubin levels, *E. coli* infection, *Candida* infection, biliary origin, abscess etiology, development of complication, pneumonia	12	Sepsis or septic shock	1	0
Thomsen 2007	Age, sex, comorbidity, alcoholism-related disorder, time period of diagnosis	5	Age, sex, comorbidity, alcoholism-related disorder, time period of diagnosis	5	5
Chen 2008a	Gas-forming liver abscess, MDRO, anaerobic infection, BUN, APACHE score, DM, uremia, malignancy, bacteremia, polymicrobial infection, non-*KP* infection, multiple abscess, bilirubin, Cre, antibiotic treatment alone	15	Gas-forming liver abscess, MDRO, anaerobic infection, BUN, APACHE score	5	2
Chen 2008b	Septic shock, acute respiratory failure, acute renal failure, APACHE II score	4	Septic shock, acute respiratory failure, acute renal failure, APACHE II score	4	2
Lee 2008	Age, sex, APACHE II score, metastatic infection, pigtail drainage, DM, ESRD, delay in presentation, septic shock, acute respiratory failure, *rmpA* gene, *magA* gene, gas-forming abscess	13	age, sex, APACHE II score, metastatic infection, pigtail drainage, DM, ESRD, delay in presentation, septic shock, acute respiratory failure, *rmpA* gene, *magA* gene, gas-forming abscess	13	4
Ng 2008	WBC, abscess size, BUN, APTT	4	WB, abscess size, BUN, APTT	4	1
Tsai 2008	Age, sex, DM, drainage, biliary procedure, peptic ulcer, urinary tract infection, renal disease, HTN, cerebrovascular accident, cholelithiasis, hepatobiliary malignancy, other malignancy, pneumonia, active viral hepatitis, heart disease, cirrhosis, *klebsiella* infection, length of hospital stays	18	Age, sex, DM, drainage, biliary procedure, peptic ulcer, urinary tract infection, renal disease, HTN, cerebrovascular accident, cholelithiasis, hepatobiliary malignancy, other malignancy, pneumonia, active viral hepatitis, heart disease	16	5
Chen 2009a	Age, APACHE II score, KP infection, hypotension	4	Age, APACHE II score, KP infection, hypotension	4	2
Chen 2009b	APACHE II score, SAPS II score, gas-forming abscess, anaerobic infection	4	APACHE II score, SAPS II score, gas-forming abscess, anaerobic infection	4	2
Chou 2009	Age, malignancy, *KP* infection, polymicrobial infection, albumin, sex, DM, cirrhosis, abscess size, abscess type, monomicrobial, *E. coli* infection, total cholesterol	13	age, malignancy, KP infection, polymicrobial infection, albumin	5	2
Foo 2010	Age, alcoholism, uremia, pathogenesis through the portal system, fever, lethargy, cough and dyspnea, jaundice, epigastric tenderness, ascites, elevated diaphragm, *E. coli* bacteremia, WBC, gas-forming abscess, Hb, prolonged coagulation time, neutrophils percentage, AST, ALT, BUN, Cre, hypoglycemia	22	Full model: age, alcoholism, uremia, pathogenesis through the portal system, fever, lethargy, cough and dyspnea, jaundice, epigastric tenderness, ascites, elevated diaphragm, *E. coli* bacteremia, WBC, gas-forming abscess, Hb, prolonged coagulation time, neutrophils percentage, AST, ALT, BUN, Cre,	Full model: 22<br>Final model: 2	2

			hypoglycemia<br>Final model: gas forming abscess, Cre		
Kuo 2010	Ag, albumin, Cre, WBC, malignancy, sex, DM, biliary stones, cirrhosis, PLT, CRP, MDRO, gas-forming abscess, abscess size, K. pneumoniae infection, symptoms to diagnosis (> 7 Days), shock, percutaneous transhepatic abscess drainage or aspiration	18	Age, albumin, Cre, WBC, malignancy	5	3
Lou 2010	APACHE II score, abscess size, gas-forming abscess	3	APACHE II score, abscess size, gas-forming abscess	3	2
Meddings 2010	Age, sex, race, insurance, hospital characteristic, hospital region, Elixhauser Comorbidity Score, cholecystectomy, bacteremia, bacteria classified elsewhere, surgery, ERCP, liver aspirate	13	Age, sex, race, insurance, hospital characteristic, hospital region, Elixhauser Comorbidity Score, cholecystectomy, bacteremia, bacteria classified elsewhere, surgery, ERCP, liver aspirate	13	5
Chen 2011	APACHE score, multi-loculation, polymicrobial infection	3	APACHE score, multi-loculation, polymicrobial infection	3	2
Kang 2011	Nephropathy, gastroenterological cancer, acute low respiratory conditions	3	Nephropathy, gastroenterological cancer, acute low respiratory conditions	3	2
Law 2011	Malignancy, albumin, DIC, ACS, Cre, bilirubin, septic shock, recurrent pyogenic cholangitis, surgery	9	Malignancy, albumin, DIC, ACS, Cre, bilirubin, septic shock, recurrent pyogenic cholangitis, surgery	9	2
Law 2012	Hepatic malignancy, albumin, DIC, ACS, renal impairment, bilirubin, septic shock, surgery	8	Hepatic malignancy, albumin, DIC, ACS, renal impairment, bilirubin, septic shock, surgery	8	2
Tian 2012	Cardiovascular system occurrence, uremia, multiple abscesses, gas-forming abscess, bacteremia/septicemia, polymicrobial infection, jaundice, bilirubin, Hb, albumin, ICU care, DM, bilobar involvement, BUN, fever duration	16	Cardiovascular system occurrence, uremia, multiple abscess, gas-forming abscess, bacteremia/septicemia, polymicrobial infection, jaundice, bilirubin, Hb, albumin, ICU care	12	1
Kuo 2013	MEDS, malignancy, multiple abscess, anaerobic infection, bilirubin, Cre	6	MEDS, malignancy, multiple abscess, anaerobic infection, bilirubin, Cre	6	3
Law 2013	Age, hepatic malignancy, albumin, bilirubin, DIC, ACS, septic shock, ICU care, renal impairment, DM, CAD, stroke, bacteremia, metastatic infection	14	Age, hepatic malignancy, albumin, bilirubin, DIC, ACS, septic shock, ICU care, renal impairment	9	3
Law 2014	Hepatic malignancy, DIC, CRP, age, albumin, ACS, septic shock	7	Hepatic malignancy, DIC, CRP, age, albumin, ACS, septic shock	7	2
Yoon 2014	Age, APACHE score	2	Age, APACHE score	2	2
Chen 2014	Sex, malignancy, respiratory distress, blood pressure, jaundice, abscess rupture, endophthalmitis, multiple organ failure	8	Nil	0	0

Hong 2014	Age, sex, dialysis modality, DM, CAD, stroke, COPD, polycystic kidney disease, malignancy, chronic liver disease, biliary tract disease, alcoholism	13	Age, sex, dialysis modality, DM, CAD, stroke, COPD, polycystic kidney disease, malignancy, chronic liver disease, biliary tract disease, alcoholism	13	5
Li 2015	Septic shock, malignancy, invasive procedure, bilirubin	4	Septic shock, malignancy, invasive procedure, bilirubin	4	1
Czerwonko 2016	Bilirubin, multiple abscess, bilobular involvement, biliary etiology, age, DM, liver transplant recipient, ALK-P, abscess size, bacteremia, polymicrobial infection, MDRO, duration of antibiotic therapy	13	Bilirubin, multiple abscess, bilobular involvement, biliary etiology	4	0
Shelat 2016	sex, age, DM, HTN, CKD, uncomplicated gallstones, other hepatobiliary diseases, ASA Physical Status Classification System score, fever, constitutional symptom, abdominal pain, jaundice, septic shock, WBC, Hb, PLT, INR, Cre, BUN, albumin, bilirubin, AST, ALT, GGT, CRP, multiple abscess, abscess size, drainage	29	Age, fever, INR, BUN	4	2
Sohn 2016	Cirrhosis, malignancy, biliary operation history, recurrence, pleural effusion, albumin, bilirubin, PLT, Hb, gas-forming abscess, DM	11	Recurrence, bilirubin, PLT, Hb	4	0
Mucke 2017	Malignancy, bilirubin, age, sex, cirrhosis, DM, proton-pump inhibitor use, immunosuppression, CRP, WBC, Cre, INR, cholangitis, mycotic coinfection, carbapenem based antibiotic, glycopeptide based antibiotic, tigecycline based antibiotic, metronidazole based antibiotic, MDRO	19	Malignancy, bilirubin	2	1
Bettinger 2018	Multiple abscess, bile duct compression, ICU care, PPI use, CCI, age, sex, immunosuppressive treatment, length of hospital stays	9	Multiple abscess, bile duct compression, ICU care, PPI use, CCI	5	4
Chen 2018	Abscess etiology, age, sex, and the duration of symptoms before admission.	4	Abscess etiology, age, sex, and the duration of symptoms before admission.	4	2
Sharma 2018	Age, sex, race, drainage, PCD, PNA, surgery, systemic disease, intra-abdominal disease, biliary disease, liver disease, malignancy, immunosuppression, cardiovascular disease	14	Nil	0	0
Park 2019	Age, sex, SBP, DBP, pulse rate, body temperature, underlying or concomitant conditions, leukocyte, lymphocyte, neutrophil, NLR, Hb, PLT, AST, ALT, ALK-P, CRP, glucose, BUN, Cre, abscess size, multiple abscess, percutaneous drainage	24	SBP, DBP, leukocyte, lymphocyte, neutrophil, NLR, Hb, CRP, abscess size	9	4
Xu 2019a	Age, sex, Hb, PLT, PT, percutaneous drainage, abscess size, Cre, DM, HTN, GNRI, low T3 syndrome	12	PLT, GNRI, low T3 syndrome	3	0
Xu 2019b	Age, sex, Hb, PLT, PT, percutaneous drainage, abscess size, Cre, DM, HTN, GNRI	11	PLT, GNRI, PT	3	0
Dai 2020	Age, sex, BMI, albumin, Hb, PLT, PT, percutaneous drainage, abscess size, Cre, DM, HTN, AST/ALT ratio	13	Albumin, PT, AST/ALT ratio	3	0
Lee 2020	DM, multiple abscess, gas-forming abscess, metastatic infection, septic shock, malignancy, bacteremia, polymicrobial infection, non-KP infection, thrombophlebitis	10	DM, multiple abscess, gas-forming abscess, metastatic infection, septic shock	5	1

Ruiz-Hernández 2020	Age, sex, polymicrobial infection, antibiotic alone, biliary origin, *E. coli* infection,	6	Age, sex, polymicrobial infection, antibiotic alone, biliary origin, *E. coli* infection,	6	2
Du 2020	DM, abscess site, E. coli infection, age, sex, smoking, drinking, hypertension, cirrhosis, biliary tract infection, WBC, Hb, PLT, bilirubin, PT, BUN, Cre, abscess number, abscess size, g gas-forming abscess, *K. pneumoniae infection*, treatments	22	DM, abscess site, E. coli infection	3	1
Yoo 2021	Sex, age, DM, cirrhosis, liver transplantation, malignancy, CKD, ICU care, residence, income	10	Sex, age, DM, cirrhosis, malignancy, CKD, ICU care	7	5
Faridi 2021	Sex, age, socio-economic status, alcoholism, smoking, DM, abscess cavities, abscess size, left lobe abscess, abscess rupture, emergency laparotomy, colon perforation, shock, time of presentation, bilirubin, albumin, WBC, APACHE score	18	Left lobe abscess, shock, time of presentation, bilirubin, albumin, WBC, APACHE score	7	2
Lee 2021a	Age, malignancy, biliary disease, decreased mentality, WBC, Hb, ALT, albumin, hs-CRP, multiple abscess, abscess size, qSOFA score, sex, AKI, DM, HTN, shock, Na, PCT, PCD, PCD timing, culture positive, KP infection, SIRS criteria	24	Age, malignancy, biliary disease, decreased mentality, WBC, Hb, ALT, albumin, hs-CRP, multiple abscess, abscess size, AKI, qSOFA score	13	3
Lee 2021b	Age, DM, HTN, SOFA, APACHE score, bacteremia, metastatic infection, concomitant infection	8	Age, DM, HTN, SOFA, APACHE score, bacteremia, metastatic infection, concomitant infection	8	3
Losie 2021	Polymicrobial bacteremia, no drainage, congestive heart failure, liver disease, bilirubin	5	Polymicrobial bacteremia, no drainage, congestive heart failure, liver disease, bilirubin	5	0
Yu 2021	Age, CCI, abscess size, malignancy, afebrile	5	Age, CCI, abscess size, malignancy, afebrile	5	4
Große 2021	Enterococcus infection, age, sex	3	Enterococcus infection, age, sex	3	2
Chan 2022	Age, ASA Physical Status Classification System score, HTN, hyperlipidemia, DM, bilirubin, Cre, albumin, ALT, multiple abscess, gas-forming abscess, renal impairment, CAD, Hb	14	Age, ASA Physical Status Classification System score, HTN, hyperlipidemia, DM, bilirubin, Cre, albumin, ALT, multiple abscess, gas-forming abscess, renal impairment, CAD, Hb	14	4
Myeong 2022	Sex, age, antibiotic, DM, cirrhosis, colon cancer, other cancer, CKD, endophthalmitis, ICU care, income, liver transplantation, vertebral osteomyelitis, pyomyositis, necrotizing fasciitis, prostate abscess	16	Sex, age, antibiotic, DM, cirrhosis, colon cancer, other cancer, CKD, endophthalmitis, ICU care	10	5
Park 2022	Inadequate antibiotics, use of inotropic agents, ICU care, age, BUN, abscess rupture, other metastatic infection, MDRO	8	Inadequate antibiotics, use of inotropic agents, ICU care	3	0
Wu 2022	Malignancy, poor appetite, pulmonary infection, ascites, gas-forming abscess, liver failure, septic shock, ICU care, abscess etiology, respiratory symptoms, Hb, PLT	12	Malignancy, poor appetite, pulmonary infection, ascites, gas-forming abscess, liver failure, septic shock	7	1

Meister 2022	Age, sex, abscess size, multiple abscess, polymicrobial infection, mycotic infection, WBC, bilirubin, AST, ALT, GGT, INR, CRP, Cre, adherence to therapy, conservative treatment, interventional treatment, escalation of treatment	18	Nil	0	0
Rossi 2022	CCI, portal thrombosis, MDRO, drainage, age, sex, hepato-biliary malignancy, hepatic metastasis, DM, malnutrition, cirrhosis, CKD, cardiomyopathy, respiratory insufficiency, liver transplantation, history of PLA, ambulatory health-care associated infection, nosocomial infection, biliary abnormality, tumoral obstruction, ischemic cholangitis, bilio-digestive anastomosis stenosis, multiple abscesses, abscess size, *E. coli, Klebsiella* spp., *Enterobacterale*s, *Enterococcus spp.*, *Streptococcus spp., Candida spp.*	30	CCI, portal thrombosis, MDRO, drainage	4	4
Jiménez-Romero 2023	Liver transplantation, GGT, PLT, Cre, Hb, vascular origin, alcoholism, hepatic artery thrombosis, portal thrombosis, Enterococcus spp.	10	Liver transplantation, PLT, Enterococcus spp.	3	0
Li 2023	Age, sex, Hb, bilirubin, Cre, WBC, ARDS, gas-forming abscess, pleural effusion, PLT, fever, lymphocyte count, ALT, PT, albumin, CRP, PCT, NT-pro-BNP, cardiac Troponin T, DM, cardiovascular disease, HTN, malignancy, liver and gallbladder stones, viral hepatitis, multi-loculation	26	Age, sex, Hb, bilirubin, Cre, WBC, ARDS, gas-forming abscess, pleural effusion, PLT	10	3
Liu 2023	WBC, neutrophil, bilirubin, age, sex, abscess location, abscess number, abscess size, body temperature, positive culture, ALT, AST, PT, BUN, Cre, CRP, ALP, PCT, albumin, non-liver cancer	21	WBC, neutrophil, bilirubin	3	0

* The core confounders denote five major covariates—age, sex, CKD (or BUN, creatinine, renal function), malignancy, and DM—accounted for in multivariate analysis. Adjustment for these confounders was also reflected in composite indices, for example, the APACHE II score (including age and renal function) and the CCI (including age, DM, CKD, and malignancy) [7].

CKD: chronic kidney disease; ALK-P: alkaline phosphatase; AST: aspartate aminotransferase; WBC: white blood count; Hb: hemoglobin; PT: Prothrombin time; APTT: activated partial thromboplastin time; DM: diabetes mellitus; CCI: Charlson Comorbidity index; Cre: creatinine; MDRO: multidrug-resistant organism; BUN: blood urea nitrogen; APACHE II: Acute Physiology and Chronic Health Evaluation II; ESRD: end-stage renal disease; SAPS II: Simplified Acute Physiology Score II; CRP: C-reactive protein; hs-CRP: high sensitivity C-reactive protein; ERCP: endoscopic retrograde cholangiopancreatography; DIC: disseminated intravascular coagulation; ACS: acute coronary syndrome; MEDS: Mortality in Emergency Department Sepsis Score; INR: International Normalized Ratio; PPI: proton pump inhibitor; SBP: systolic blood pressure; DBP: diastolic blood pressure; NLR: neutrophil-to-lymphocyte ratio; ALT: alanine aminotransferase; GNRI: Geriatric Nutritional Risk Index; PLT: platelet; HTN: hypertension; KP: *K. pneumoniae*; *E. coli: Escherichia coli*; AKI: acute kidney injury; aSOFA: quick Sequential Organ Failure Assessment; SIRS: systemic inflammatory response syndrome; PCD: percutaneous catheter drainage; ASA: American Society of Anesthesiologists; CAD: coronary artery disease; GGT: gamma-glutamyl transferase; PCT: procalcitonin.

Table S5. Subgroup analyses of prognostic factors

Factor	Category	Subgroups	Number of studies	Sample size	aOR [95% CI], p value (Inverse variance method and random-effects model)	Heterogeneity (I^2, p value)	Test for subgroup differences (I^2, p value)
Age (older vs younger)	All	All	9	3,048	2.33 [1.29 – 4.19], p = 0.005	77%, p < 0.001	NA
	Cut-off value	Age ≧ 75	1	120	2.86 [0.64, 12.86], p = 0.17	NA	47.6% p = 0.13
		Age ≧ 64 - 66	6	1,886	1.71 [1.01, 2.89], p = 0.05	50%, p = 0.07	
		Age ≧ 60	1	377	7.20 [1.09, 47.40], p = 0.04	NA	
		Age ≧ 55	1	665	3.90 [2.27, 6.70], p < 0.000001	NA	
	Outcome	In-hospital mortality	8	2,383	2.00 [1.17, 3.41], p = 0.01	54%, p = 0.03	66.4% p = 0.08
		30-day mortality	1	665	3.90 [2.27, 6.70], p < 0.00001	77%, p < 0.0001	
	Risk of bias (overall QUIPS)	Low risk	2	439	3.08 [1.40, 6.80], p = 0.005	0%, p = 0.91	0% p = 0.51
		Moderate or high risk	7	2,609	2.16 [1.09, 4.28], p = 0.03	79%, p < 0.0001	
	Country	Asian	8	2,383	2.00 [1.17, 3.41], p = 0.01	54%, p = 0.03	66.4% p = 0.08
		Non-Asian	1	665	3.90 [2.27, 6.70], p < 0.00001	77%, p < 0.0001	
	Year of publication	2001-2010	5	1,629	3.29 [2.19, 4.95], p < 0.00001	0%, p = 0.63	93.5% p < 0.00001
		2011-2020	1	319	3.17 [1.25, 8.04], p = 0.02	NA	
		2021-2023	3	1,100	1.10 [1.00, 1.21], p = 0.04	0%, p = 0.53	
	Sample size	Sample size < 300	4	682	1.11 [1.01, 1.22], p = 0.03	0%, p = 0.42	96.5% p < 0.00001
		Sample size > 300	5	2,366	3.28 [2.23, 4.82], p < 0.00001	0%, p = 0.53	
Female sex	All	All	6	80,403	1.18 [1.04 – 1.33], p = 0.01	66%, p = 0.01	NA
	Outcome	In-hospital mortality	4	78,290	1.09 [1.03, 1.15], p = 0.004	0%, p = 0.60	82.4% P = 0.02
		30-day mortality	2	2,113	1.65 [1.18, 2.31], p = 0.004	52%, p = 0.15	

	Risk of bias (overall QUIPS)	Low risk	2	60,393	1.10 [1.03, 1.17], p = 0.003	77%, p = 0.004	38.1% P = 0.20
		Moderate to high risk	4	20,010	1.39 [0.97, 1.99], p = 0.07	0%, p = 0.37	
	Country	Asian	6	60,503	1.10 [1.03, 1.17], p = 0.003	0%, p = 0.58	26.0% P = 0.25
		Non-Asian	3	19,900	1.38 [0.94, 2.03], p = 0.10	85%, p = 0.001	
	Year of publication	2001-2010	5	49,713	1.23 [1.02, 1.49], p = 0.03	72%, p = 0.006	0% p = 0.47
		2021-2023	1	30,690	1.14 [1.03, 1.26], p = 0.01	NA	
	Sample size	Sample size < 300	1	110	1.67 [0.34, 8.33], p = 0.53	NA	0% p = 0.67
		Sample size > 300	5	80,293	1.18 [1.04, 1.34], p = 0.01	72%, p = 0.006	
Malignancy	All	All	16	65,972	5.63 [3.39, 9.36], p < 0.00001	80%, p < 0.00001	NA
	Pathology	Any malignancy type	15	65,658	5.92 [3.46, 10.13], p < 0.00001	82%, p < 0.00001	0% p = 0.41
		Hepatobiliary malignancy	1	314	3.47 [1.11, 10.87], p < 0.00001	NA	
	Risk of bias (overall QUIPS)	Low risk	6	63,120	3.28 [1.89, 5.68], p < 0.0001	84%, p < 0.00001	77.2% p = 0.04
		Moderate or high risk	10	2,852	10.17 [4.11, 25.18], p < 0.00001	62%, p = 0.004	
	Country	Asian	15	65,886	5.67 [3.35, 9.61], p < 0.00001	82%, p < 0.00001	0% p = 0.93
		Non-Asian	1	86	5.26 [1.06, 26.04], p = 0.04	NA	
	Year of publication	2001-2010	7	30,848	3.12 [1.59, 6.14], p = 0.001	48%, p = 0.07	40.7% p = 0.19
		2011-2020	5	3,427	11.62 [3.39, 39.83], p < 0.0001	75%, p = 0.003	
		2021-2023	4	31,697	4.41 [3.40, 5.72], p < 0.00001	0%, p = 0.49	
	Sample size	Sample size < 300	8	1,191	4.44 [2.49, 7.92], p < 0.00001	88%, p < 0.00001	52.8% p = 0.15
		Sample size > 300	8	64,781	10.35 [3.87, 27.64], p < 0.00001	32%, p = 0.17	
Diabetes mellitus	All	All	6	62,349	1.06 [0.83, 1.36], p = 0.64	80%, p = 0.0001	NA
	Outcome	In-hospital mortality	4	60,583	1.01 [0.76, 1.36], p = 0.93	87%, p < 0.0001	0% p = 0.55
		30-day mortality	1	324	0.92 [0.27, 3.19], p = 0.90	NA	

		30-day post-discharge mortality	1	142	1.34 [0.88, 2.05], p = 0.18	NA	
	Risk of bias (overall QUIPS)	Low risk	2	60,393	0.99 [0.73, 1.33], p = 0.93	95%, p < 0.00001	28.9%
		Moderate or high risk	4	1,956	1.33 [0.90, 1.97], p = 0.15	0%, p = 0.57	p = 0.24
	Country	Asian	5	60,907	1.01 [0.76, 1.33], p = 0.96	82%, p = 0.0001	17.7%
		Non-Asian	1	1,442	1.34 [0.88, 2.05], p = 0.18	NA	p = 0.27
	Year of publication	2001-2010	4	31,335	1.07 [0.69, 1.67], p = 0.75	56%, p = 0.08	0%
		2021-2023	2	31,014	1.15 [1.04, 1.27], p = 0.006	0%, p = 0.73	p = 0.77
	Sample size	Sample size < 300	2	190	2.71 [0.33, 22.09], p = 0.35	20%, p = 0.26	0%
		Sample size > 300	4	62,159	1.04 [0.81, 1.34], p = 0.74	87%, p < 0.0001	p = 0.38
Chronic kidney disease	All	All	8	64,077	2.41 [1.42, 4.07], p = 0.001	93%, p < 0.00001	NA
	Severity	Uremia	4	1,046	6.27 [1.34, 29.33], p = 0.02	51%, p = 0.11	48.3%
		Any stage	4	63,031	1.95 [1.10, 3.45], p = 0.02	97%, p < 0.00001	p = 0.16
	Risk of bias (overall QUIPS)	Low risk	4	62,914	2.67 [1.61, 4.43], p = 0.001	86%, p < 0.0001	0%
		Moderate or high risk	4	1,163	2.09 [0.65, 6.68], p = 0.22	61%, p = 0.05	p = 0.70
	Year of publication	2001-2010	4	30,392	3.56 [1.09, 11.63], p = 0.04	53%, p = 0.10	26.3%
		2011-2020	3	2,995	3.28 [0.70, 15.43], p = 0.13	90%, p < 0.0001	p = 0.26
		2021-2023	1	30,690	1.53 [1.20, 1.94], p = 0.0005	NA	
	Sample size	Sample size < 300	2	312	17.57 [2.27, 136.23], p = 0.006	18%, p = 0.27	74.5%
		Sample size > 300	6	63,765	2.07 [1.22, 3.53], p = 0.007	95%, p < 0.00001	p = 0.05
Higher APACHE II score	All	All	4	1,026	12.42 [5.85, 26.37], p < 0.00001	0%, p = 0.88	NA
	Cut-off value	APACHE II ≧ 15	2	592	9.94 [3.56, 27.79], p < 0.0001	0%, p = 0.76	0%
		APACHE II ≧ 16	1	110	11.82 [1.94, 72.02], p = 0.007	NA	p = 0.75
		APACHE II ≧ 18	1	324	19.31 [4.77, 78.21], p < 0.0001	NA	
	Outcome	In-hospital mortality	3	702	10.37 [4.25, 25.34], p < 0.00001	0%, p = 0.94	0%

		30-day mortality	1	324	19.31 [4.77, 78.21], p < 0.0001	NA	p = 0.46
	Year of publication	2001-2010	3	702	10.37 [4.25, 25.34], p < 0.00001	0%, p = 0.94	0%
		2021-2023	1	324	19.31 [4.77, 78.21], p < 0.0001	NA	p = 0.46
	Sample size	Sample size < 300	2	363	9.66 [3.13, 29.80], p < 0.0001	0%, p = 0.78	0%
		Sample size > 300	2	663	15.21 [5.53, 41.85], p < 0.00001	0%, p = 0.63	p = 0.56
Abscess size (per 1-cm increase)	All	All	3	280	1.64 [0.77, 3.51], p = 0.20	97%, p < 0.00001	NA
	Outcome	In-hospital mortality	2	245	1.19 [0.74, 1.94], p = 0.47	86%, p = 0.008	91.3%
		ICU mortality	1	35	2.99 [2.39, 3.72], p < 0.00001	NA	p = 0.0007
	Year of publication	2001-2010	2	178	2.19 [1.16, 4.12], p = 0.02	90%, p = 0.001	83.9%
		2011-2020	1	102	0.95 [0.82, 1.12], p = 0.55	97%, p < 0.00001	p = 0.01
Multiple abscesses	All	All	8	2,044	2.95 [0.89, 9.78], p = 0.08	80%, p < 0.00001	NA
	Country	Asian	5	1588	3.94 [0.88, 17.71], p = 0.07	76%, p = 0.002	0%
		Non-Asian	3	456	1.91 [0.22, 16.71], p = 0.56	83%, p = 0.002	p = 0.59
	Year of publication	2001-2010	3	285	13.69 [4.02, 46.60], p < 0.0001	0%, p = 0.47	87.0% p = 0.0005
		2011-2020	4	1111	1.93 [0.44, 8.52], p = 0.39	83%, p = 0.0005	
		2021-2023	1	648	0.29 [0.07, 1.31], p = 0.11	80%, p < 0.00001	
	Sample size	Sample size < 300	5	608	4.87 [0.67, 35.44], p = 0.12	83%, p = 0.0001	0%
		Sample size > 300	3	1436	1.80 [0.33, 9.77], p = 0.50	82%, p = 0.004	p = 0.45
Leukocytosis	All	All	4	1,218	1.87 [0.30, 11.68], p = 0.50	77%, p = 0.004	NA
	Risk of bias (overall QUIPS)	Low risk	1	120	2.46 [0.52, 11.53], p = 0.25	NA	0%
		Moderate or high risk	3	1098	1.85 [0.14, 25.30], p = 0.64	84%, p = 0.002	p = 0.85
	Year of publication	Before 2000	1	73	51.59 [4.04, 659.35], p = 0.002	NA	75.5% p = 0.02
		2001-2010	2	497	0.83 [0.11, 6.56], p = 0.86	75%, p = 0.05	
		2021-2023	1	648	0.75 [0.13, 4.27], p = 0.74	NA	
	Sample size	Sample size < 300	2	193	9.45 [0.49, 182.80], p = 0.14	75%, p = 0.05	73.0%

		Sample size > 300	2	497	0.43 [0.14, 1.26], p = 0.12	0%, p = 0.42	p = 0.05
Anemia	All	All	5	1,706	4.33 [1.05, 17.91], p = 0.04	68%, p = 0.01	NA
	Cut-off value	Hb < 14 (men), or 12 (women)	1	377	6.60 [0.73, 59.61], p = 0.09	NA	0% p = 0.85
		Hb < 12	2	588	6.75 [1.76, 25.95], p = 0.005	0%, p = 0.45	
		Hb < 10	2	781	2.26 [0.07, 75.77], p = 0.65	91%, p = 0.001	
	Country	Asian	4	1613	3.12 [0.61, 16.05], p = 0.17	66%, p = 0.03	41.5% p = 0.19
		Non-Asian	1	133	13.33 [3.17, 56.00], p = 0.004	NA	
	Year of publication	2001-2010	2	510	10.81 [3.25, 35.96], p = 0.0001	0%, p = 0.60	83.0% p = 0.003
		2011-2020	2	588	6.75 [1.76, 25.95], p = 0.005	0%, p = 0.45	
		2021-2023	1	648	0.37 [0.08, 1.82], p = 0.22	68%, p = 0.01	
	Sample size	Sample size < 300	2	364	13.31 [4.00, 44.27], p < 0.0001	0%, p = 1.00	64.0% p = 0.10
		Sample size > 300	3	1382	2.04 [0.32, 12.97], p = 0.45	68%, p = 0.04	
Thrombocytopenia	All	All	3	929	4.18 [2.05, 8.50], p < 0.0001	0%, p = 0.51	NA
	Cut-off value	Platelet < 125 x 10^9/L	2	698	4.28 [1.59, 11.52], p = 0.004	26%, p = 0.25	0% p = 0.97
		Platelet < 140 x 10^9/L	1	231	4.40 [1.13, 17.10], p = 0.03	NA	
	Year of publication	2011-2020	2	471	3.36 [1.48, 7.61], p = 0.004	0%, p = 0.63	10.2% p = 0.29
		2021-2023	1	458	8.16 [1.95, 34.22], p = 0.004	NA	
	Sample size	Sample size < 300	2	471	3.36 [1.48, 7.61], p = 0.004	0%, p = 0.63	10.2% p = 0.29
		Sample size > 300	1	458	8.16 [1.95, 34.22], p = 0.004	NA	
Hypoalbuminemia	All	All	8	2,186	4.12 [2.60, 6.53], p < 0.00001	0%, p = 0.73	NA
	Cut-off level	Albumin < 3.5 mg/dl	1	357	6.40 [0.46, 89.60], p = 0.17	NA	0% p = 0.90
		Albumin < 3.0 mg/dl	3	1008	5.22 [2.18, 12.48], p = 0.0002	0%, p = 0.72	
		Albumin < 2.8 mg/dl	1	319	3.42 [1.35, 8.69], p = 0.010	NA	
		Albumin < 2.5 mg/dl	3	502	5.25 [1.67, 16.47], p = 0.004	36%, p = 0.21	

	Risk of bias (overall QUIPS)	Low risk	2	439	4.25 [1.70, 10.64], p = 0.002	5%, p = 0.30	0% p = 0.95
		Moderate or high risk	6	1747	4.10 [2.37, 7.10], p < 0.00001	0%, p = 0.65	
	Year of publication	Before 2000	1	73	11.42 [1.71, 76.37], p = 0.01	NA	0% p = 0.78
		2001-2010	3	549	5.00 [1.61, 15.49], p = 0.005	31%, p = 0.23	
		2011-2020	3	916	3.94 [1.93, 8.06], p = 0.0002	0%, p = 0.87	
		2021-2023	1	648	4.28 [0.94, 19.59], p = 0.06	NA	
	Sample size	Sample size < 300	4	505	7.50 [3.13, 18.01], p < 0.00001	0%, p = 0.72	60.0% p = 0.11
		Sample size > 300	4	1681	3.27 [1.90, 5.63], p < 0.0001	0%, p = 0.91	
Hyperbilirubinemia	All	All	6	1,752	8.36 [2.35, 29.72], p = 0.001	0%, p = 0.43	NA
	Cut-off level	Total bilirubin > 1.17-1.75 mg/dl	3	1022	5.17 [0.74, 36.00], p = 0.10	80%, p = 0.007	0% p = 0.44
		Total bilirubin > 2.0 mg/dl	1	231	9.54 [2.38, 38.22], p = 0.001	NA	
		Total bilirubin > 5.0mg/dl	2	499	34.45 [3.82, 310.74], p = 0.002	0%, p = 0.43	
	Risk of bias (overall QUIPS)	Low risk	1	319	1.54 [0.65, 3.68], p = 0.33	NA	92.6% p = 0.0002
		Moderate or high risk	5	1433	14.49 [6.39, 32.85], p < 0.00001	0%, p = 0.80	
	Country	Asian	5	1610	6.08 [1.71, 21.65], p = 0.005	65%, p = 0.02	58.6% p = 0.12
		Non-Asian	1	142	60.11 [4.49, 804.65], p = 0.002	NA	
	Sample size	Sample size < 300	3	645	13.75 [4.25, 44.47], p < 0.0001	0%, p = 0.46	0% p = 0.40
		Sample size > 300	3	1107	5.17 [0.74, 36.00], p = 0.10	80%, p = 0.007	
Azotemia	All	All	4	1,027	5.12 [0.84, 31.24], p = 0.08	80%, p = 0.002	NA
	Cut-off value	BUN > 22 mg/dl	2	630	2.80 [0.03, 256.48], p = 0.66	90%, p = 0.002	0% p = 0.63
		BUN > 28 mg/dl	2	397	8.57 [3.42, 21.44], p < 0.00001	0%, p = 0.43	
	Outcome	In-hospital mortality	2	630	2.80 [0.03, 256.48], p = 0.66	90%, p = 0.002	0%

		In-hospital or 30-day mortality	2	397	8.57 [3.42, 21.44], p < 0.00001	0%, p = 0.43	p = 0.63
	Risk of bias (overall QUIPS)	Low risk	1	264	6.64 [2.17, 20.30], p = 0.0009	NA	0%
		Moderate or high risk	3	763	4.81 [0.27, 84.45], p = 0.28	86%, p = 0.0007	p = 0.84
	Country	Asian	1	133	3.65 [0.33, 40.92], p = 0.29	84%, p = 0.002	0%
		Non-Asian	3	894	14.49 [2.91, 72.08], p = 0.001	NA	p = 0.35
	Year of publication	2001-2010	3	763	4.81 [0.27, 84.45], p = 0.28	86%, p = 0.0007	0%
		2011-2020	1	264	6.64 [2.17, 20.30], p = 0.0009	NA	p = 0.84
	Sample size	Sample size < 300	3	650	10.16 [4.33, 23.85], p < 0.00001	0%, p = 0.45	92.6%
		Sample size > 300	1	377	0.30 [0.06, 1.60], p = 0.16	NA	p = 0.0002
Impaired renal function	All	All	4	1,207	6.42 [1.61, 25.59], p = 0.008	76%, p = 0.006	NA
	Cut-off value	Creatinine > 1.3 mg/dl	2	808	16.77 [2.89, 97.34], p = 0.002	69%, p = 0.07	67.4%
		Creatinine > 1.5 mg/dl	1	120	5.39 [1.40, 20.76], p = 0.01	NA	p = 0.05
		Creatinine > 2.26 mg/dl	1	319	1.11 [0.28, 4.33], p = 0.88	NA	
	Risk of bias (overall QUIPS)	Low risk	2	439	2.45 [0.52, 11.54], p = 0.26	62%, p = 0.11	61.3%
		Moderate or high risk	2	808	16.77 [2.89, 97.34], p = 0.002	69%, p = 0.07	p = 0.11
	Sample size	Sample size < 300	1	120	5.39 [1.40, 20.76], p = 0.01	NA	0%
		Sample size > 300	3	1127	6.90 [0.97, 49.21], p = 0.05	84%, p = 0.002	p = 0.84
Percutaneous drainage	All	All	4	47,736	0.48 [0.37, 0.63], p < 0.00001	76%, p = 0.007	NA
	Outcome	In-hospital mortality	3	47,600	0.50 [0.39, 0.64], p < 0.00001	80%, p = 0.007	54.2%
		30-day mortality	1	136	0.08 [0.01, 0.91], p = 0.04	NA	p = 0.14
	Risk of bias (overall QUIPS)	Low risk	1	29,703	0.57 [0.52, 0.62], p < 0.00001	NA	50.1%
		Moderate or high risk	3	18,033	0.27 [0.09, 0.76], p = 0.01	46%, p = 0.16	p = 0.16
	Country	Asian	2	29,813	0.37 [0.10, 1.32], p = 0.13	61%, p = 0.11	0%
		Non-Asian	2	17,923	0.29 [0.06, 1.31], p = 0.11	49%, p = 0.16	p = 0.80

	Year of publication	2001-2010	3	47,600	0.50 [0.39, 0.64], p < 0.00001	80%, p = 0.007	54.2% p = 0.14
		2021-2023	1	136	0.08 [0.01, 0.91], p = 0.04	NA	
	Sample size	Sample size < 300	2	246	0.11 [0.03, 0.47], p = 0.003	0%, p = 0.69	76.0% p = 0.04
		Sample size > 300	2	47,490	0.51 [0.40, 0.64], p < 0.00001	87%, p = 0.006	

* Significant with p value less than 0.05

CI: Confidence Interval; NA: Not Applicable; QUIPS: Quality In Prognosis Studies tool; APACHE II: Acute Physiology and Chronic Health Evaluation II; Hb: Hemoglobin; BUN: Blood Urea Nitrogen.

Table S6. Sensitivity analyses according to adjustment status

Factor	Category	Number of studies	Sample size	aOR [95% CI], p value (Inverse variance method and random-effects model)	Heterogeneity (I^2, *p* value)
Age (older vs younger)	adjusted	9	3,048	2.33 [1.29 – 4.19], p = 0.005	77%, *p* < 0.001
	unadjusted	10	2,692	1.97 [1.29 – 3.01], p = 0.002	64%, *p* = 0.003
Age (per 1-year increase) [1]	adjusted	4	30,559	1.02 [1.01 – 1.04], p = 0.01	27%, *p* = 0.25
	unadjusted	4	30,179	1.03 [1.01 – 1.04], p = 0.03	45%, *p* = 0.14
Female sex	adjusted	6	80,403	1.18 [1.04 – 1.33], p = 0.01	66%, *p* = 0.01
	unadjusted	11	62,579	1.17 [1.04 – 1.31], p = 0.01	31%, *p* = 0.15
Malignancy	adjusted	16	65,972	5.63 [3.39, 9.36], p < 0.00001	80%, *p* < 0.00001
	unadjusted	10	62,334	3.73 [2.49, 5.58], p < 0.00001	80%, *p* < 0.00001
Chronic kidney disease	adjusted	8	64,077	2.41 [1.42, 4.07], p = 0.001	93%, *p* < 0.00001
	unadjusted	6	61,450	2.91 [2.18, 3.88], p < 0.00001	65%, *p* = 0.01
Diabetes mellitus	adjusted	6	62,349	1.06 [0.83, 1.36], p = 0.64	80%, *p* = 0.0001
	unadjusted	14	64,433	1.22 [0.89, 1.68], p = 0.21	91%, *p* < 0.00001
Liver cirrhosis	adjusted	2	31,355	1.95 [0.45, 8.49], p = 0.37	89%, *p* = 0.003
	unadjusted	5	61,067	1.52 [1.00, 2.30], p = 0.05	86%, *p* < 0.00001
Fever*	adjusted	3	880	0.29 [0.13, 0.68], p = 0.004	0%, *p* = 0.65
	unadjusted	3	1,034	0.53 [0.12, 2.29], p = 0.4	83%, *p* = 0.003
Higher APACHE II score	adjusted	4	1,026	12.42 [5.85, 26.37], p < 0.00001	0%, *p* = 0.88
	unadjusted	3	687	24.01 [12.35, 46.67], p < 0.00001	0%, *p* = 0.84
Septic shock	adjusted	7	2,046	9.14 [4.54, 18.42], p < 0.00001	0%, *p* = 0.68

	unadjusted	3	1,162	13.33 [6.90, 25.78], p < 0.00001	0%, *p* = 0.93
Jaundice*	adjusted	2	734	1.08 [0.01, 137.40], p = 0.98	66%, *p* = 0.09
	unadjusted	2	694	5.27 [1.44, 19.32], p = 0.01	63%, *p* = 0.1
Abscess size (per 1-cm increase) [1]	adjusted	3	280	1.64 [0.77, 3.51], p = 0.2	97%, *p* < 0.00001
	unadjusted	2	101	1.19 [0.84, 1.68], p = 0.33	68%, *p* = 0.08
Multiple abscesses	adjusted	8	2,044	2.95 [0.89, 9.78], p = 0.08	80%, *p* < 0.00001
	unadjusted	10	2,361	2.00 [0.97, 4.11], p = 0.06	75%, *p* < 0.00001
Gas-forming abscess	adjusted	5	1,262	10.16 [4.01, 25.71], p < 0.00001	15%, *p* = 0.32
	unadjusted	8	2,040	4.79 [3.24, 7.08], p < 0.00001	0%, *p* = 0.68
Serum WBC count (per 10^9/L increase) *, [1]	adjusted	2	245	1.02 [0.99, 1.05], p = 0.21	43%, *p* = 0.18
	unadjusted	3	610	1.04 [1.01, 1.08], p = 0.02	0%, *p* = 0.81
Anemia*	adjusted	5	1,706	4.33 [1.05, 17.91], p = 0.04	68%, *p* = 0.01
	unadjusted	4	1,223	2.04 [0.69, 6.05], p = 0.2	81%, *p* = 0.001
Thrombocytopenia	adjusted	3	929	4.18 [2.05, 8.50], p < 0.0001	0%, *p* = 0.51
	unadjusted	4	1,218	4.01 [1.57, 10.29], p = 0.004	77%, *p* = 0.004
Hypoalbuminemia	adjusted	8	2,186	4.12 [2.60, 6.53], p < 0.00001	0%, *p* = 0.73
	unadjusted	5	1,833	2.75 [1.73, 4.39], p < 0.0001	57%, *p* = 0.05
Hyperbilirubinemia	adjusted	6	1,752	8.36 [2.35, 29.72], p = 0.001	0%, *p* = 0.43
	unadjusted	6	1,217	8.81 [5.15, 15.09], p < 0.00001	0%, *p* = 0.84
Azotemia*	adjusted	4	1,027	5.12 [0.84, 31.24], p = 0.08	80%, *p* = 0.002
	unadjusted	4	1,820	3.47 [1.40, 8.61], p = 0.007	72%, *p* = 0.01
Impaired renal function	adjusted	4	1,207	6.42 [1.61, 25.59], p = 0.008	76%, *p* = 0.006
	unadjusted	4	1,119	3.67 [1.41, 9.52], p = 0.008	76%, *p* = 0.006
Bacteremia	adjusted	5	18,981	3.26 [1.53, 6.94], p = 0.002	37%, *p* = 0.18

	unadjusted	6	1,632	3.41 [2.03, 5.74], p < 0.00001	28%, *p* = 0.22
Klebsiella spp. infection	adjusted	3	30,399	0.29 [0.16, 0.54], p < 0.0001	0%, *p* = 0.54
	unadjusted	4	1,477	0.37 [0.23, 0.59], p < 0.001	0%, *p* = 0.94
Escherichia coli infection	adjusted	3	772	2.84 [1.30, 6.21], p = 0.009	0%, *p* = 0.90
	unadjusted	2	734	2.92 [1.22, 7.01], p = 0.02	0%, *p* = 0.48
Polymicrobial infection*	adjusted	4	1,043	2.09 [0.73, 5.97], p = 0.17	47%, *p* = 0.13
	unadjusted	7	1,530	3.73 [2.24, 6.21], p < 0.00001	37%, *p* = 0.15
Multidrug-resistant organism	adjusted	3	812	8.43 [2.90, 24.53], p < 0.0001	0%, *p* = 0.41
	unadjusted	5	1,671	4.07 [1.94, 8.56], p = 0.0002	54%, *p* = 0.07
Pneumonia	adjusted	2	29,823	1.52 [1.33, 1.72], p < 0.00001*	0%, *p* = 0.96
	unadjusted	2	30,161	6.24 [0.44, 87.83], p = 0.18	93%, *p* = 0.0002
ICU admission	adjusted	5	32,380	5.12 [3.84, 6.83], p < 0.00001	10%, *p* = 0.35
	unadjusted	3	31,704	4.78 [2.35, 9.72], p < 0.0001	67%, *p* = 0.05
Metastatic infection	adjusted	4	878	5.34 [2.32, 12.32], p < 0.0001	0%, *p* = 0.46
	unadjusted	4	1,486	6.76 [3.78, 12.09], p < 0.00001	0%, *p* = 0.44
Percutaneous drainage	adjusted	4	47,736	0.48 [0.37, 0.63], p < 0.00001	76%, *p* = 0.007
	unadjusted	5	30,837	0.45 [0.28, 0.73], p = 0.001	43%, *p* = 0.14
Surgical drainage	adjusted	2	18,105	0.92 [0.74, 1.15], p = 0.46	0%, *p* = 0.32
	unadjusted	2	106	0.87 [0.26, 2.87], p = 0.82	23%, *p* = 0.26

[1] Continuous variable, each 1-unit increase

*Different significance of factors between adjusted and unadjusted results in this sensitivity analysis

CI: Confidence Interval; APACHE II: Acute Physiology and Chronic Health Evaluation II; WBC: white blood cell; ICU: intensive care unit.

Table S7. Univariate random-effects meta-regression for the effect of malignancy on mortality

Variable	Number of studies	Sample size	Effect size (slope estimate)	95% confidence interval	*p* value
Risk of bias (low risk vs others)	16	65,972	-1.0022	-1.9186 to -0.0859	0.0321
Country (Asian vs non-Asian)	16	65,972	0.0755	-2.1264 to 2.2774	0.9464
Hospital number (multiple hospitals vs. single hospital)	16	65,972	-0.5876	-1.5429 to 0.3677	0.2280
Mean age	14	65,495	-0.1730	-0.3281 to -0.0178	0.0289
Publication year	16	65,972	0.0053	-0.0962 to 0.1069	0.9178
Study sample size	16	65,972	-0.0000	-0.0001 to 0.0000	0.1014
Mortality rate	16	65,972	4.5624	-5.2698 to 14.3945	0.3631
Number of adjustment factors	16	65,972	-0.0702	-0.1776 to 0.0372	0.1999
Percentage of male sex	16	65,972	-8.2412	-26.2115 to 9.7290	0.3687
Percentage of diabetes mellitus	16	65,972	2.2411	-2.1412 to 6.6234	0.3162
Percentage of *Klebsiella spp. infection*	14	32,963	-2.0225	-5.0117 to 0.9667	0.1848
Percentage of malignancy	15	65,700	-0.8865	-7.9123 to 6.1393	0.8047

Table S8. Sensitivity analysis according to peer-review status

Factor	Category	Number of studies	Sample size	aOR [95% CI], p value (Inverse variance method and random-effects model)	Heterogeneity (I^2, *p* value)
Hypoalbuminemia	All	8	2,186	4.12 [2.60, 6.53], p < 0.00001	0%, p = 0.73
	Excluding preprint	7	1946	4.05 [2.46, 6.67], p < 0.00001	0%, p = 0.63

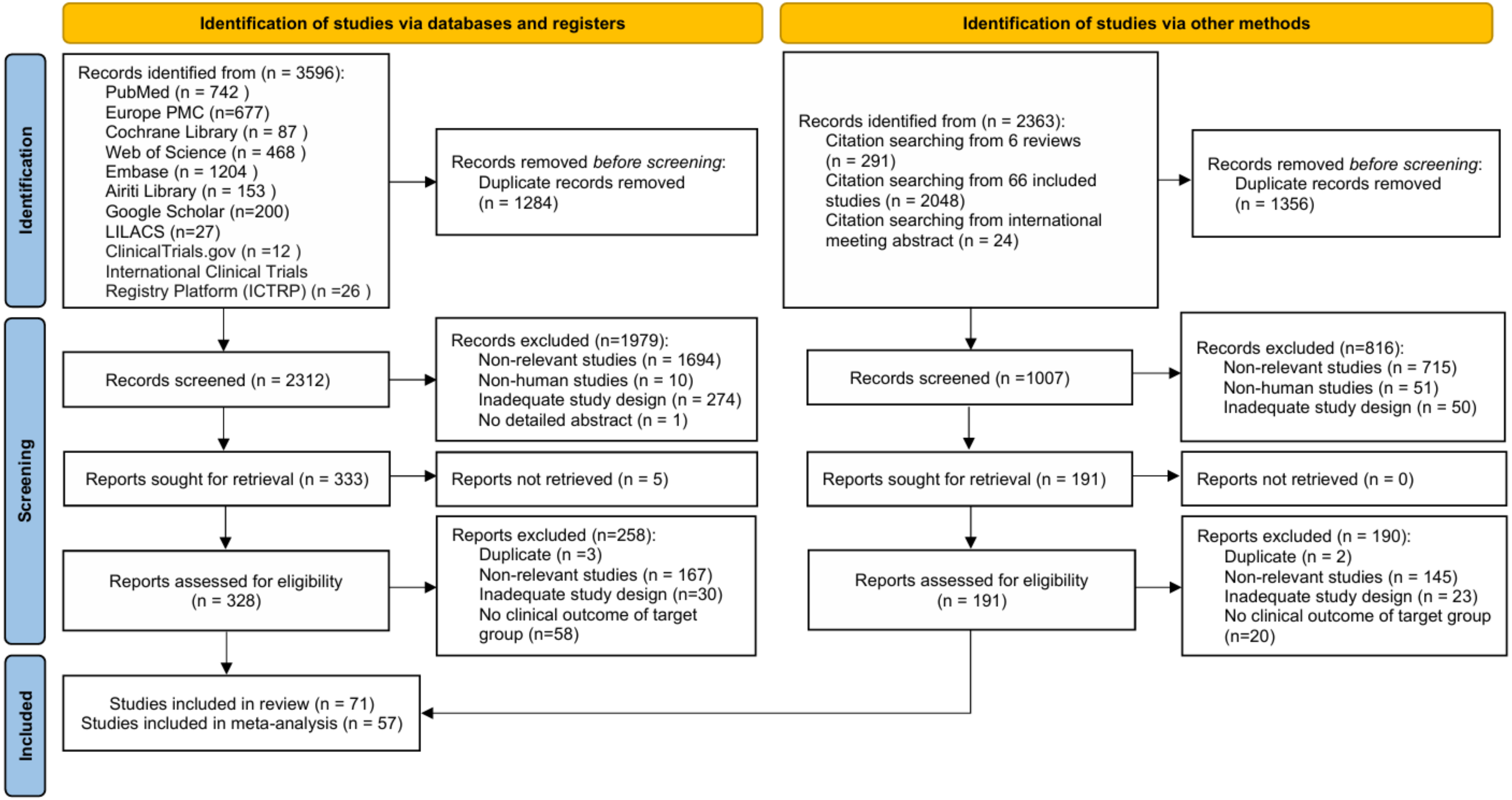

Figure S1 PRISMA flow diagram

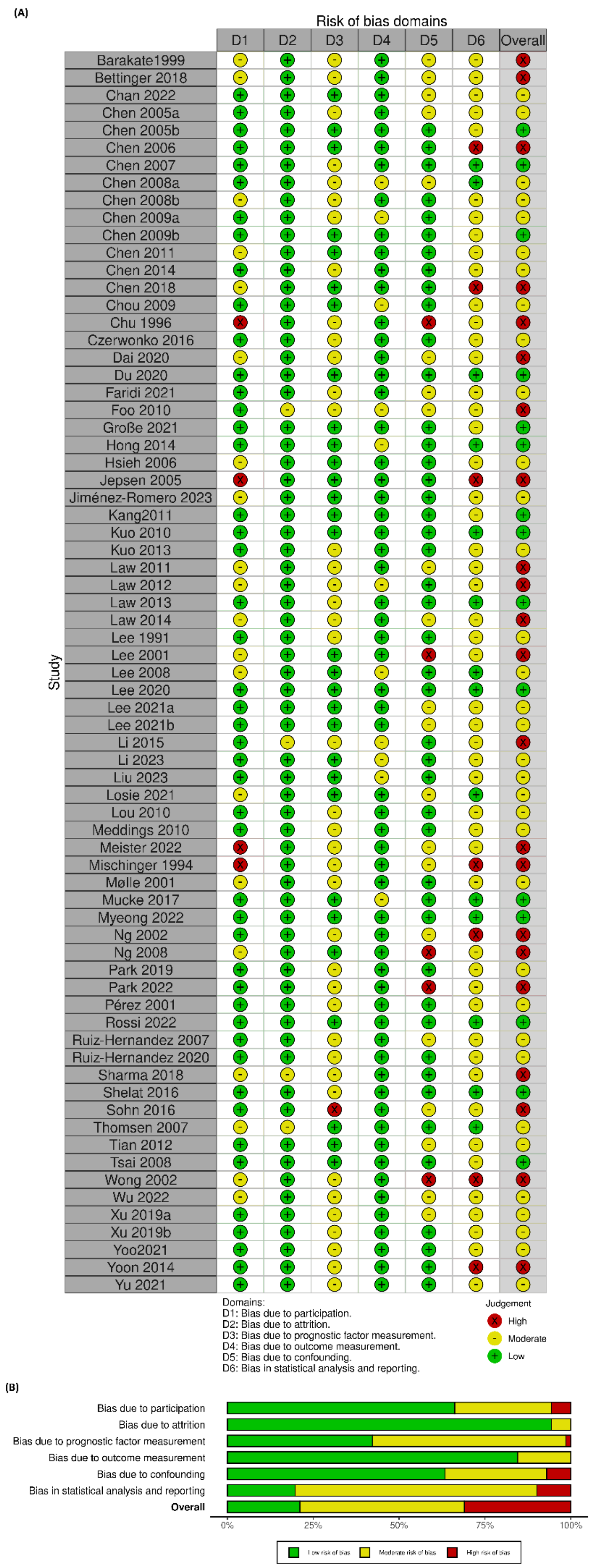

Figure S2 Risk of bias graph (A) and risk of bias summary (B) for all included studies.

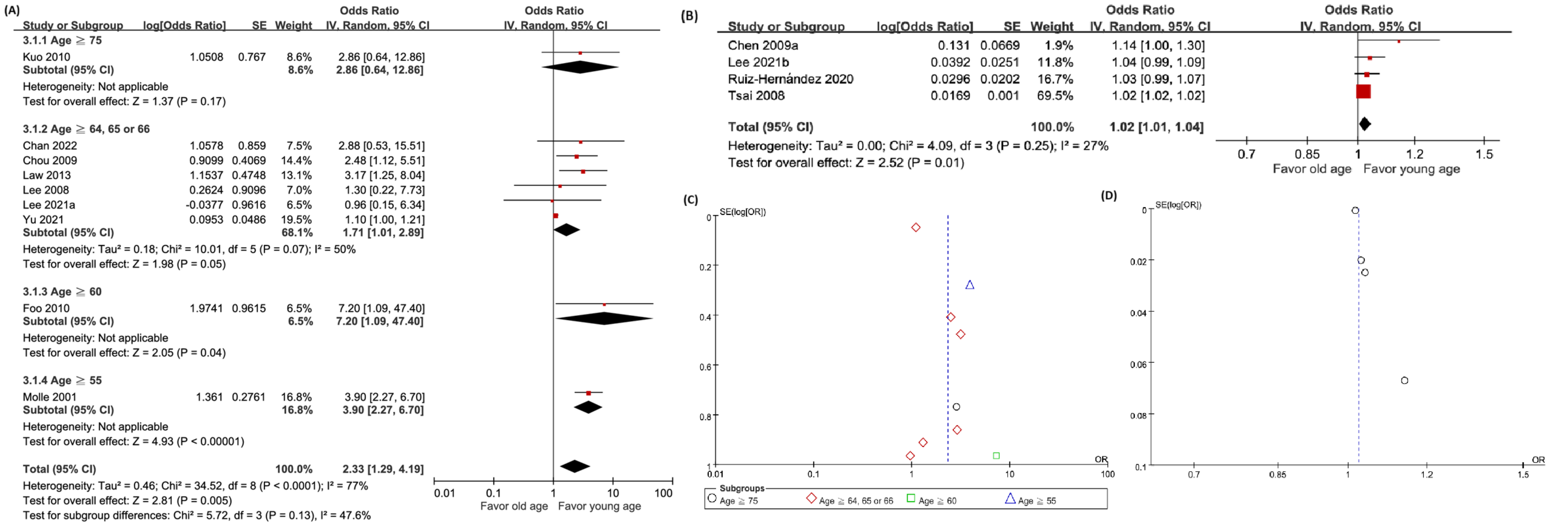

Figure S3. Forest and funnel plots of the association between age and short-term mortality in pyogenic liver abscess. (A) Forest plot of age (older vs younger; subgroup by cut-off value); (B) Forest plot of age (per 1-year increase); (C) Funnel plot of age (older vs younger; subgroup by cut-off value); (D) Funnel plot of age (per 1-year increase).

Study or Subgroup	log[Odds Ratio]	SE	Weight	Odds Ratio IV, Random, 95% CI
**3.3.1 In-hospital mortality**				
Lee 2008	0.5146	0.819	0.6%	1.67 [0.34, 8.33]
Meddings 2010	0.0237	0.0723	23.0%	1.02 [0.89, 1.18]
Myeong 2022	0.131	0.0518	27.1%	1.14 [1.03, 1.26]
Tsai 2008	0.0723	0.0395	29.3%	1.07 [0.99, 1.16]
**Subtotal (95% CI)**			**80.0%**	**1.09 [1.03, 1.15]**
Heterogeneity: Tau² = 0.00; Chi² = 1.88, df = 3 (P = 0.60); I² = 0%				
Test for overall effect: Z = 2.89 (P = 0.004)				
**3.3.2 30-day mortality**				
Jepsen 2005	0.3464	0.1475	11.7%	1.41 [1.06, 1.89]
Molle 2001	0.6931	0.1896	8.2%	2.00 [1.38, 2.90]
**Subtotal (95% CI)**			**20.0%**	**1.65 [1.18, 2.31]**
Heterogeneity: Tau² = 0.03; Chi² = 2.08, df = 1 (P = 0.15); I² = 52%				
Test for overall effect: Z = 2.90 (P = 0.004)				
**Total (95% CI)**			**100.0%**	**1.18 [1.04, 1.33]**
Heterogeneity: Tau² = 0.01; Chi² = 14.75, df = 5 (P = 0.01); I² = 66%				
Test for overall effect: Z = 2.59 (P = 0.010)				
Test for subgroup differences: Chi² = 5.68, df = 1 (P = 0.02), I² = 82.4%				

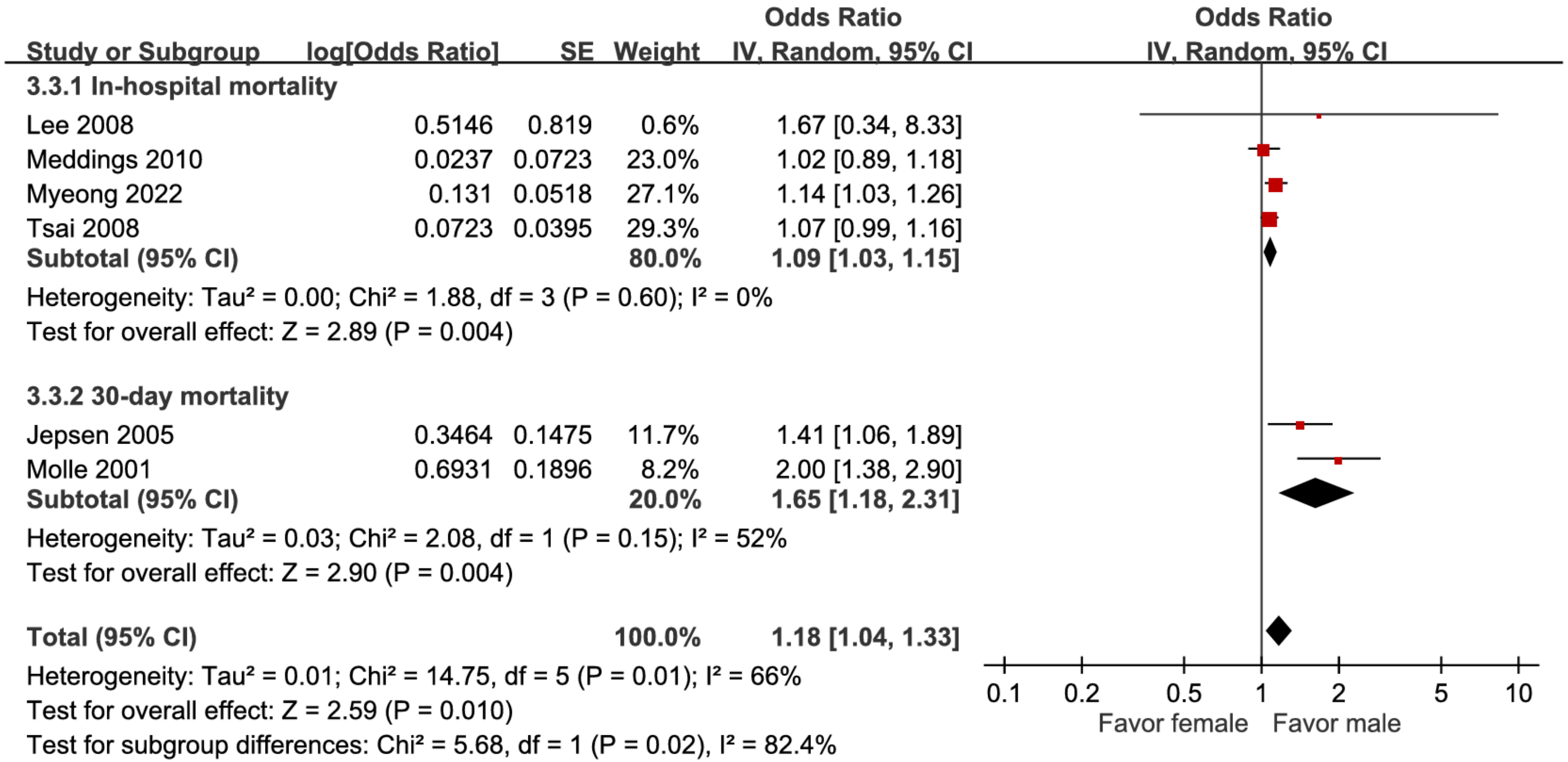

Figure S4. Forest plot of the association between sex (female vs male) and short-term mortality in pyogenic liver abscess.

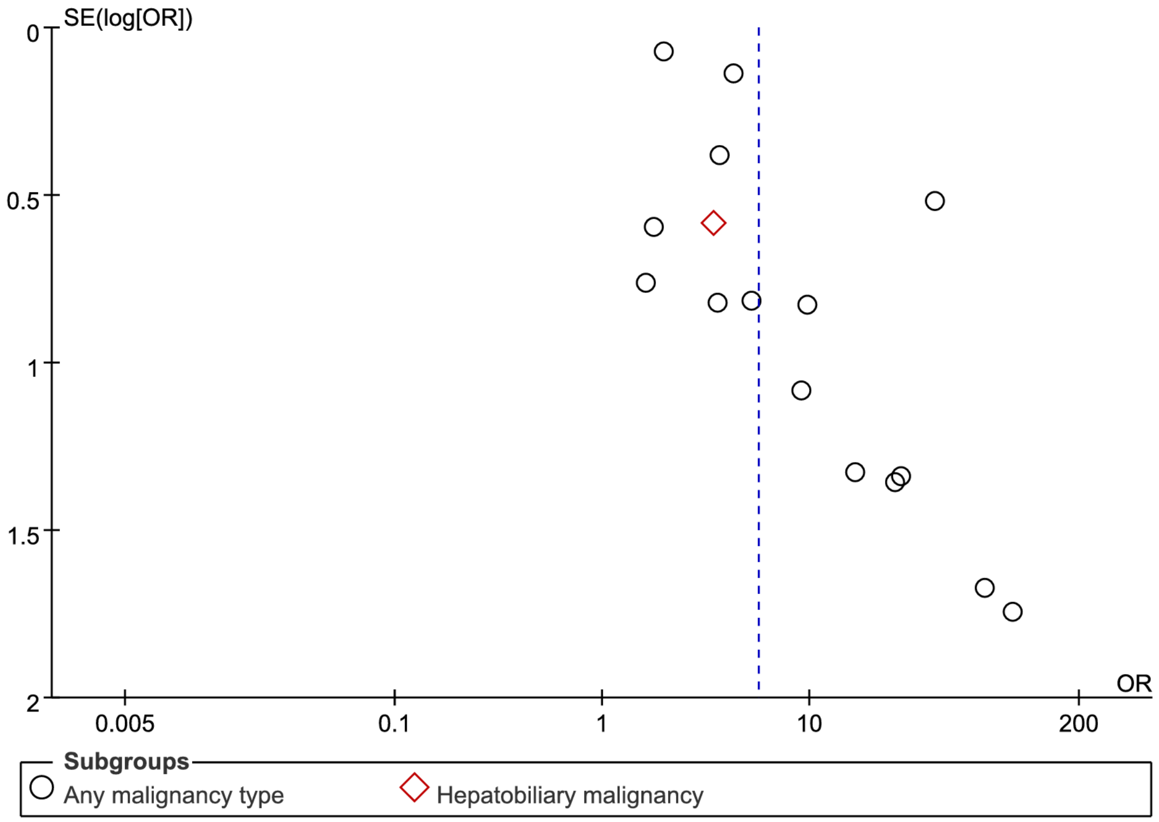

Figure S5. Funnel plot of the association between malignancy and short-term mortality in pyogenic liver abscess.

Study or Subgroup	log[Odds Ratio]	SE	Weight	Odds Ratio IV, Random, 95% CI
Lee 2008	0.3646	0.9706	1.7%	1.44 [0.21, 9.65]
Lee 2021b	-0.0834	0.6344	3.7%	0.92 [0.27, 3.19]
Myeong 2022	0.1398	0.0506	37.5%	1.15 [1.04, 1.27]
Thomsen 2007	0.2927	0.2169	18.6%	1.34 [0.88, 2.05]
Tsai 2008	-0.1625	0.0437	38.1%	0.85 [0.78, 0.93]
Wong 2002	2.8094	1.9564	0.4%	16.60 [0.36, 768.06]
**Total (95% CI)**			**100.0%**	**1.06 [0.83, 1.36]**

Heterogeneity: Tau² = 0.04; Chi² = 24.92, df = 5 (P = 0.0001); I² = 80%

Test for overall effect: Z = 0.47 (P = 0.64)

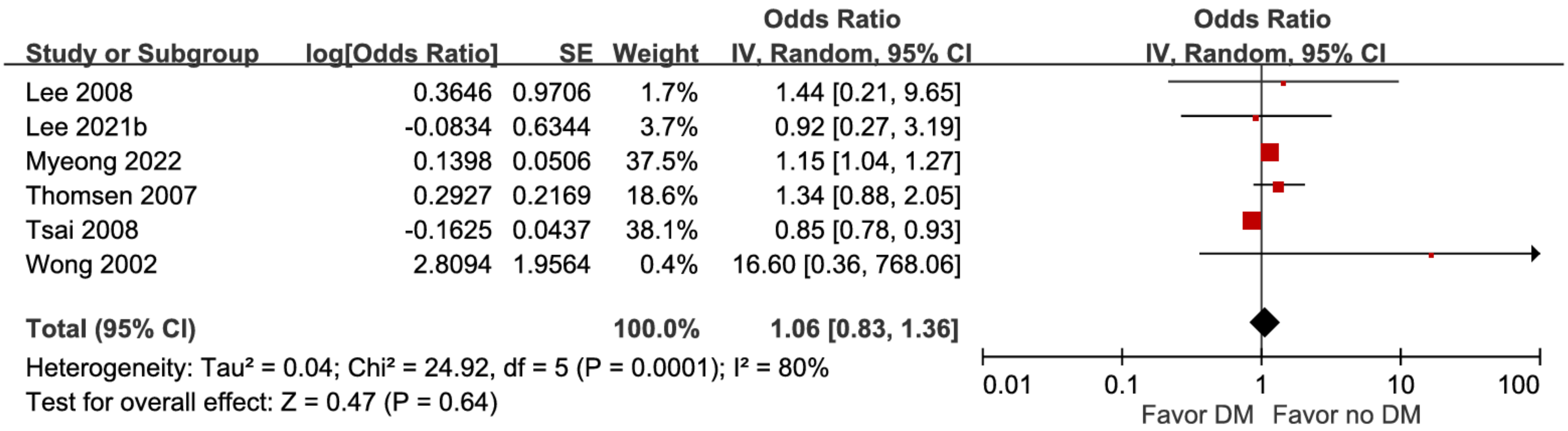

Figure S6. Forest plot of the association between diabetes mellitus and short-term mortality in pyogenic liver abscess.

Study or Subgroup	log[Odds Ratio]	SE	Weight	Odds Ratio IV, Random, 95% CI
Molle 2001	1.5019	0.4986	44.8%	4.49 [1.69, 11.93]
Myeong 2022	-0.005	0.1057	55.2%	1.00 [0.81, 1.22]
**Total (95% CI)**			**100.0%**	**1.95 [0.45, 8.49]**

Heterogeneity: $Tau^2 = 1.01$; $Chi^2 = 8.74$, df = 1 (P = 0.003); $I^2 = 89\%$
Test for overall effect: Z = 0.89 (P = 0.37)

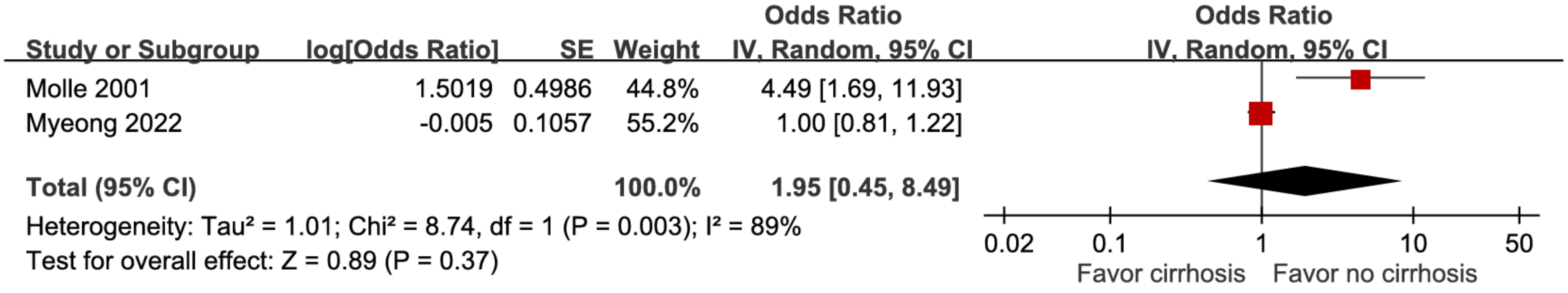

Figure S7. Forest plot of the association between liver cirrhosis and short-term mortality in pyogenic liver abscess.

Study or Subgroup	log[Odds Ratio]	SE	Weight	Odds Ratio IV, Random, 95% CI	Year
Tsai 2008	-0.8324	0.0771	56.8%	0.44 [0.37, 0.51]	2008
Lee 2021b	0.94	0.6565	43.2%	2.56 [0.71, 9.27]	2021
**Total (95% CI)**			**100.0%**	**0.94 [0.17, 5.23]**	

Heterogeneity: Tau² = 1.35; Chi² = 7.19, df = 1 (P = 0.007); I² = 86%
Test for overall effect: Z = 0.08 (P = 0.94)

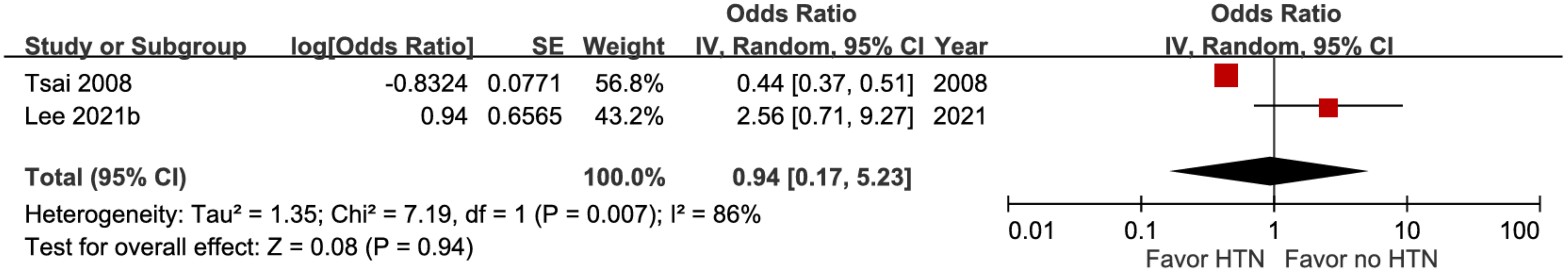

Figure S8. Forest plot of the association between hypertension and short-term mortality in pyogenic liver abscess.

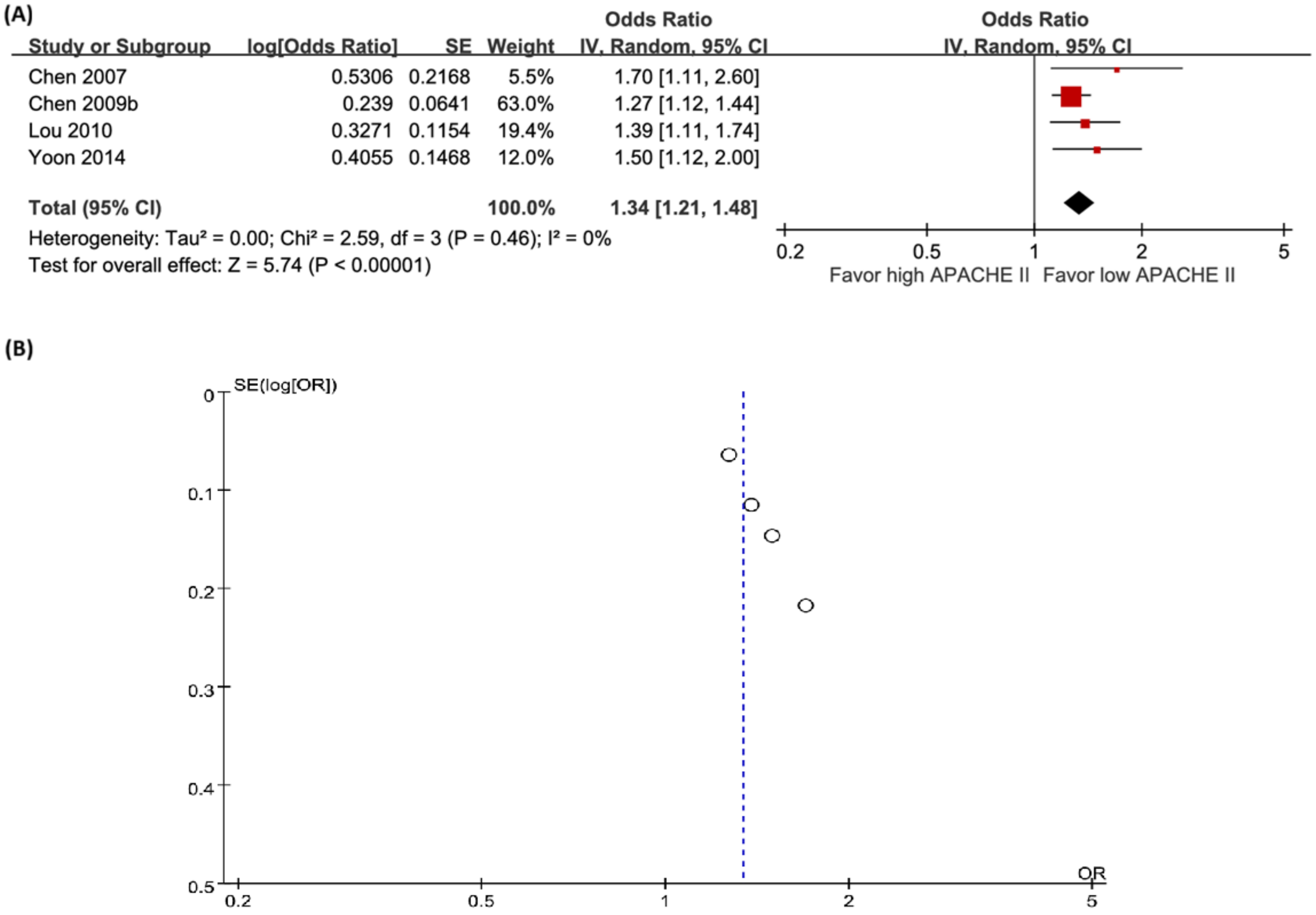

Figure S9. Forest and funnel plots of the association between the APACHE II score (per 1-point increase) and short-term mortality in pyogenic liver abscess.

Study or Subgroup	log[Odds Ratio]	SE	Weight	Odds Ratio IV, Random, 95% CI	Year
Foo 2010	-2.3026	1.9307	51.9%	0.10 [0.00, 4.40]	2010
Tian 2012	2.6462	2.1606	48.1%	14.10 [0.20, 973.50]	2012
**Total (95% CI)**			**100.0%**	**1.08 [0.01, 137.40]**	

Heterogeneity: Tau² = 8.05; Chi² = 2.92, df = 1 (P = 0.09); I² = 66%
Test for overall effect: Z = 0.03 (P = 0.98)

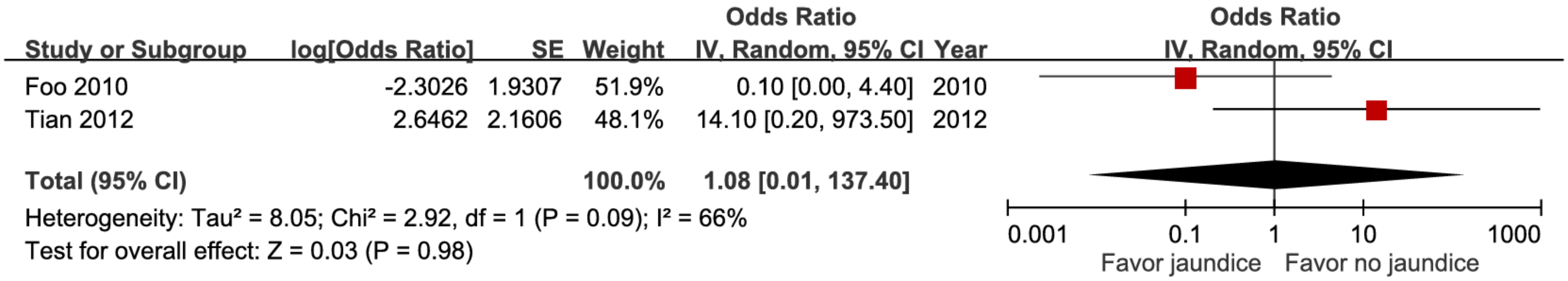

Figure S10. Forest plot of the association between jaundice and short-term mortality in pyogenic liver abscess.

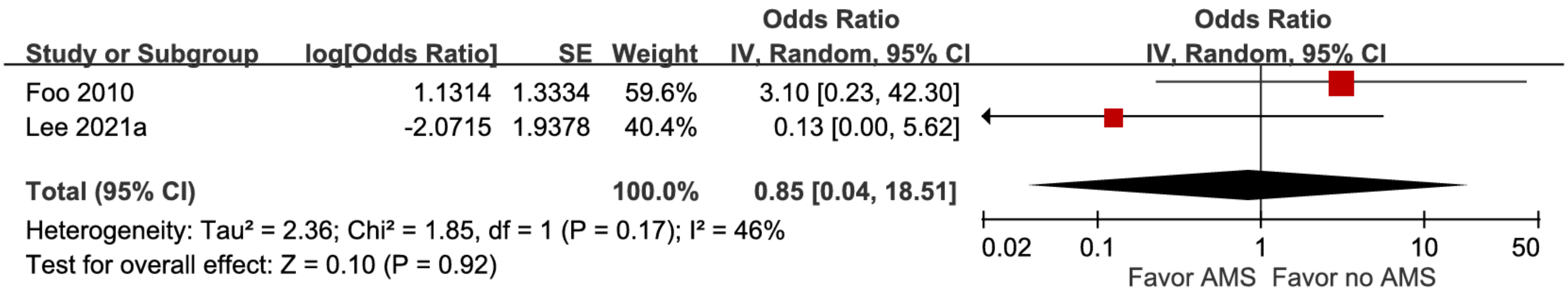

Figure S11. Forest plot of the association between altered mental status (AMS) and short-term mortality in pyogenic liver abscess.

Study or Subgroup	log[Odds Ratio]	SE	Weight	Odds Ratio IV, Random, 95% CI	Year
Pérez 2001	1.6477	0.7456	19.6%	5.20 [1.20, 22.40]	2001
Chen 2005b	0.3577	0.8942	15.8%	1.43 [0.25, 8.25]	2005
Czerwonko 2016	1.0869	0.8629	16.5%	2.97 [0.55, 16.09]	2016
Ruiz-Hernández 2020	0.3577	0.4523	30.4%	1.43 [0.59, 3.47]	2020
Lee 2021a	-1.2242	0.8142	17.7%	0.29 [0.06, 1.45]	2021
**Total (95% CI)**			**100.0%**	**1.57 [0.66, 3.73]**	

Heterogeneity: $Tau^2 = 0.44$; $Chi^2 = 7.41$, df = 4 (P = 0.12); $I^2 = 46\%$

Test for overall effect: Z = 1.02 (P = 0.31)

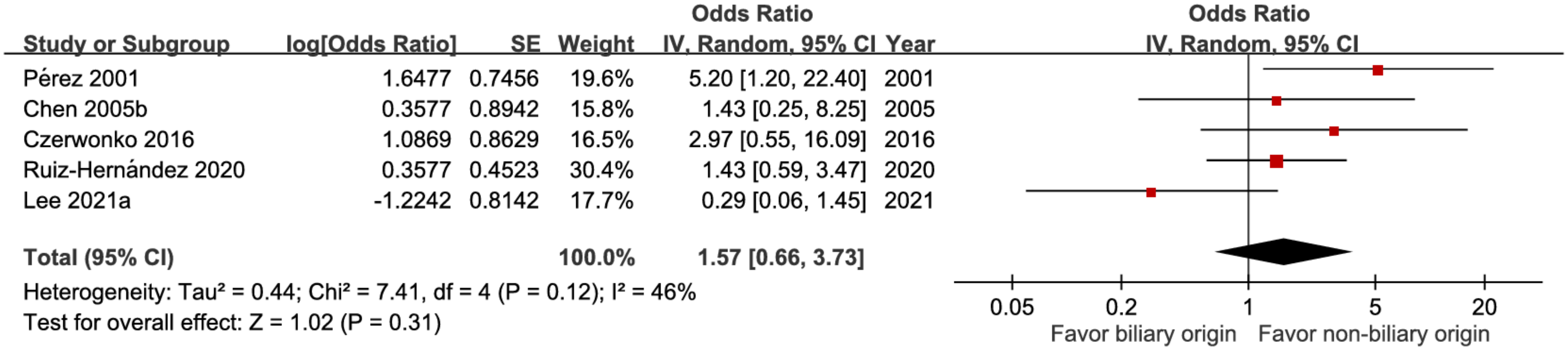

Figure S12. Forest plot of the association between biliary origin and short-term mortality in pyogenic liver abscess.

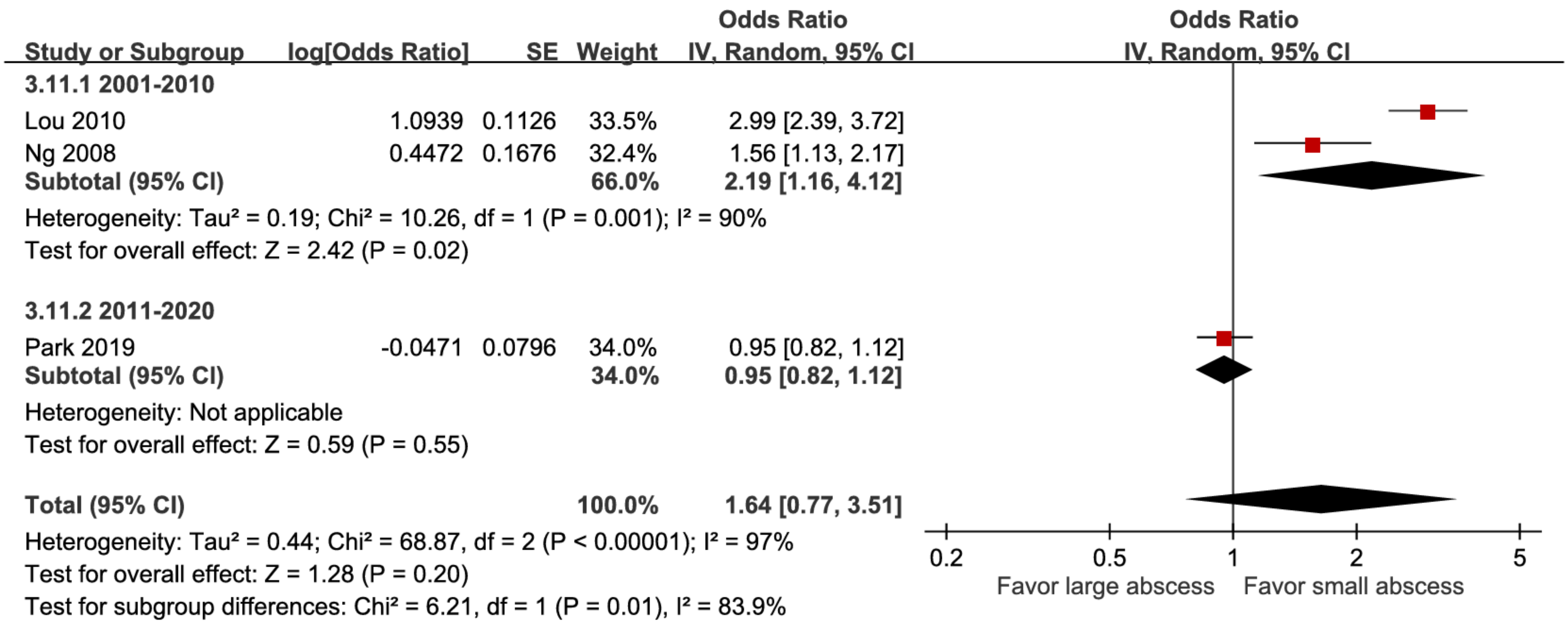

Figure S13. Forest plot of the association between abscess size (per 1-cm increase; subgroup by year of publication) and short-term mortality in pyogenic liver abscess.

Study or Subgroup	log[Odds Ratio]	SE	Weight	Odds Ratio IV, Random, 95% CI	Year
**3.12.1 2001-2010**					
Pérez 2001	2.1736	0.798	13.3%	8.79 [1.84, 42.00]	2001
Wong 2002	2.4849	1.4146	9.0%	12.00 [0.75, 191.99]	2002
Chen 2005a	4.1713	1.4293	8.9%	64.80 [3.94, 1067.07]	2005
**Subtotal (95% CI)**			**31.1%**	**13.69 [4.02, 46.60]**	
Heterogeneity: Tau² = 0.00; Chi² = 1.50, df = 2 (P = 0.47); I² = 0%					
Test for overall effect: Z = 4.19 (P < 0.0001)					
**3.12.2 2011-2020**					
Tian 2012	0.9933	0.6831	14.2%	2.70 [0.71, 10.30]	2012
Kuo 2013	1.775	0.4961	15.4%	5.90 [2.23, 15.60]	2013
Czerwonko 2016	0.9123	1.2717	9.9%	2.49 [0.21, 30.11]	2016
Bettinger 2018	-0.863	0.4203	15.9%	0.42 [0.19, 0.96]	2018
**Subtotal (95% CI)**			**55.3%**	**1.93 [0.44, 8.52]**	
Heterogeneity: Tau² = 1.78; Chi² = 17.63, df = 3 (P = 0.0005); I² = 83%					
Test for overall effect: Z = 0.87 (P = 0.39)					
**3.12.3 2021-2023**					
Lee 2021a	-1.2276	0.7649	13.5%	0.29 [0.07, 1.31]	2021
**Subtotal (95% CI)**			**13.5%**	**0.29 [0.07, 1.31]**	
Heterogeneity: Not applicable					
Test for overall effect: Z = 1.60 (P = 0.11)					
**Total (95% CI)**			**100.0%**	**2.95 [0.89, 9.78]**	
Heterogeneity: Tau² = 2.17; Chi² = 35.72, df = 7 (P < 0.00001); I² = 80%					
Test for overall effect: Z = 1.77 (P = 0.08)					
Test for subgroup differences: Chi² = 15.36, df = 2 (P = 0.0005), I² = 87.0%					

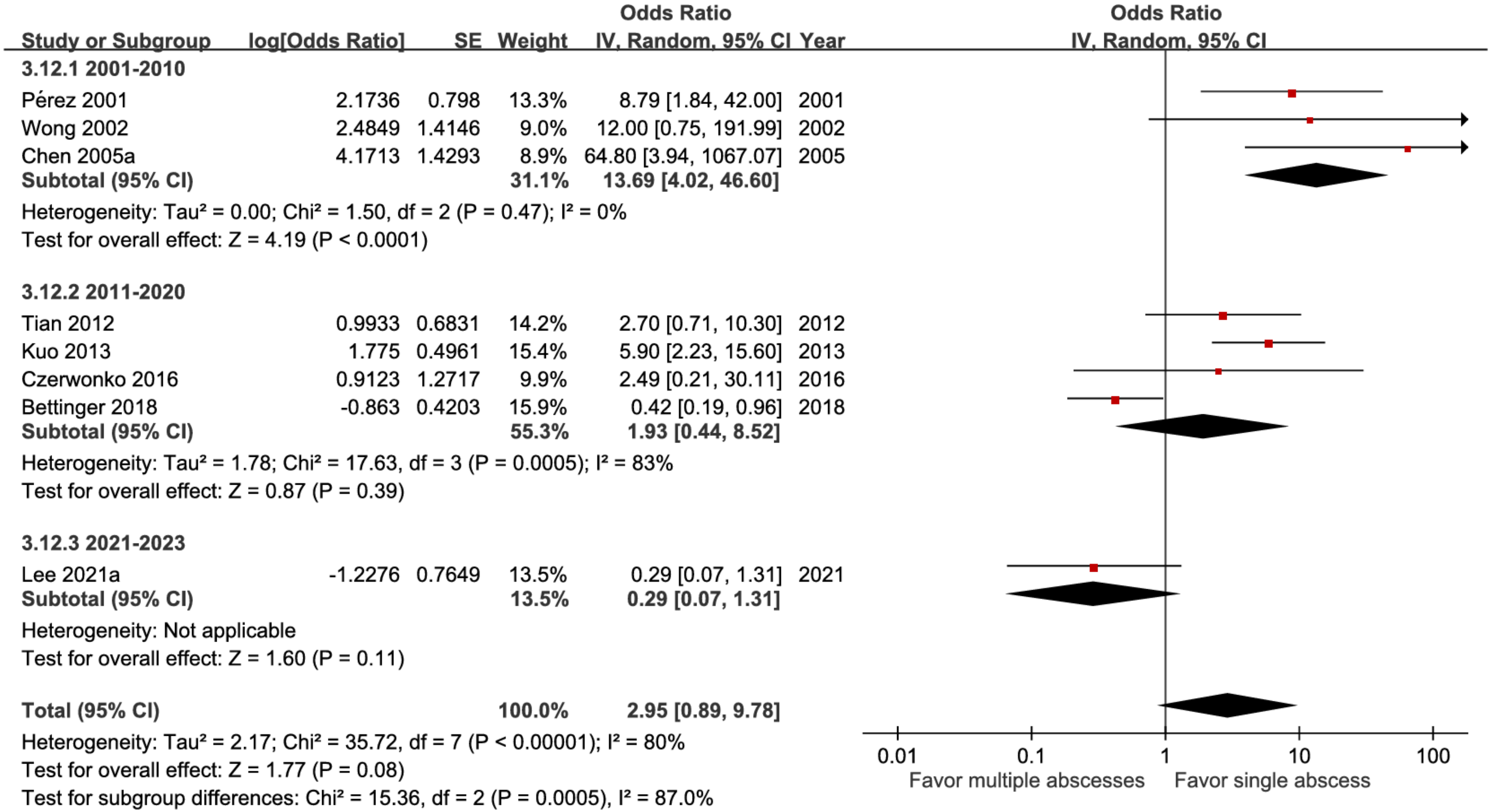

Figure S14. Forest plot of the association between multiple abscesses (subgroup by year of publication) and short-term mortality in pyogenic liver abscess.

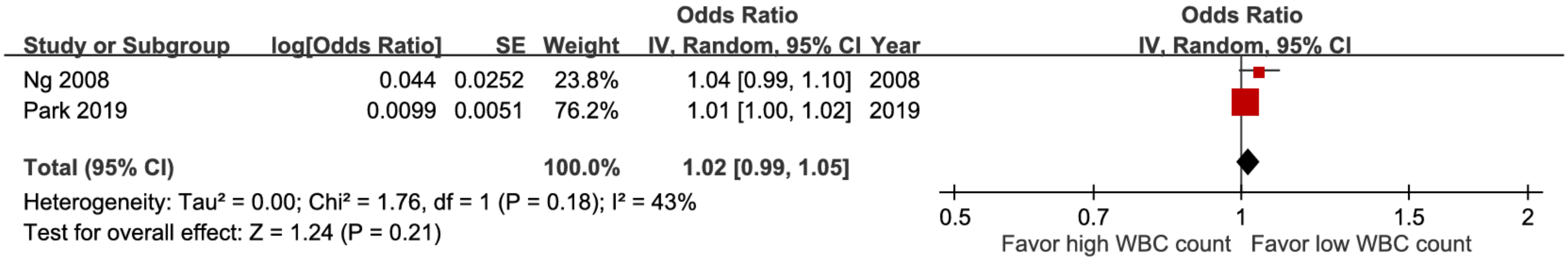

Figure S15. Forest plot of the association between serum white blood cell (WBC) count (per 10^9/L increase) and short-term mortality in pyogenic liver abscess.

Study or Subgroup	log[Odds Ratio]	SE	Weight	Odds Ratio IV, Random, 95% CI	Year
**3.15.1 before 2000**					
Lee 1991	3.9433	1.3	20.1%	51.59 [4.04, 659.35]	1991
**Subtotal (95% CI)**			**20.1%**	**51.59 [4.04, 659.35]**	
Heterogeneity: Not applicable					
Test for overall effect: Z = 3.03 (P = 0.002)					
**3.15.2 2001-2010**					
Kuo 2010	0.9002	0.7882	26.7%	2.46 [0.52, 11.53]	2010
Foo 2010	-1.204	0.7073	27.7%	0.30 [0.07, 1.20]	2010
**Subtotal (95% CI)**			**54.5%**	**0.83 [0.11, 6.56]**	
Heterogeneity: Tau² = 1.65; Chi² = 3.95, df = 1 (P = 0.05); I² = 75%					
Test for overall effect: Z = 0.17 (P = 0.86)					
**3.15.3 2021-2023**					
Lee 2021a	-0.2917	0.8896	25.4%	0.75 [0.13, 4.27]	2021
**Subtotal (95% CI)**			**25.4%**	**0.75 [0.13, 4.27]**	
Heterogeneity: Not applicable					
Test for overall effect: Z = 0.33 (P = 0.74)					
**Total (95% CI)**			**100.0%**	**1.87 [0.30, 11.68]**	
Heterogeneity: Tau² = 2.65; Chi² = 13.32, df = 3 (P = 0.004); I² = 77%					
Test for overall effect: Z = 0.67 (P = 0.50)					
Test for subgroup differences: Chi² = 8.16, df = 2 (P = 0.02), I² = 75.5%					

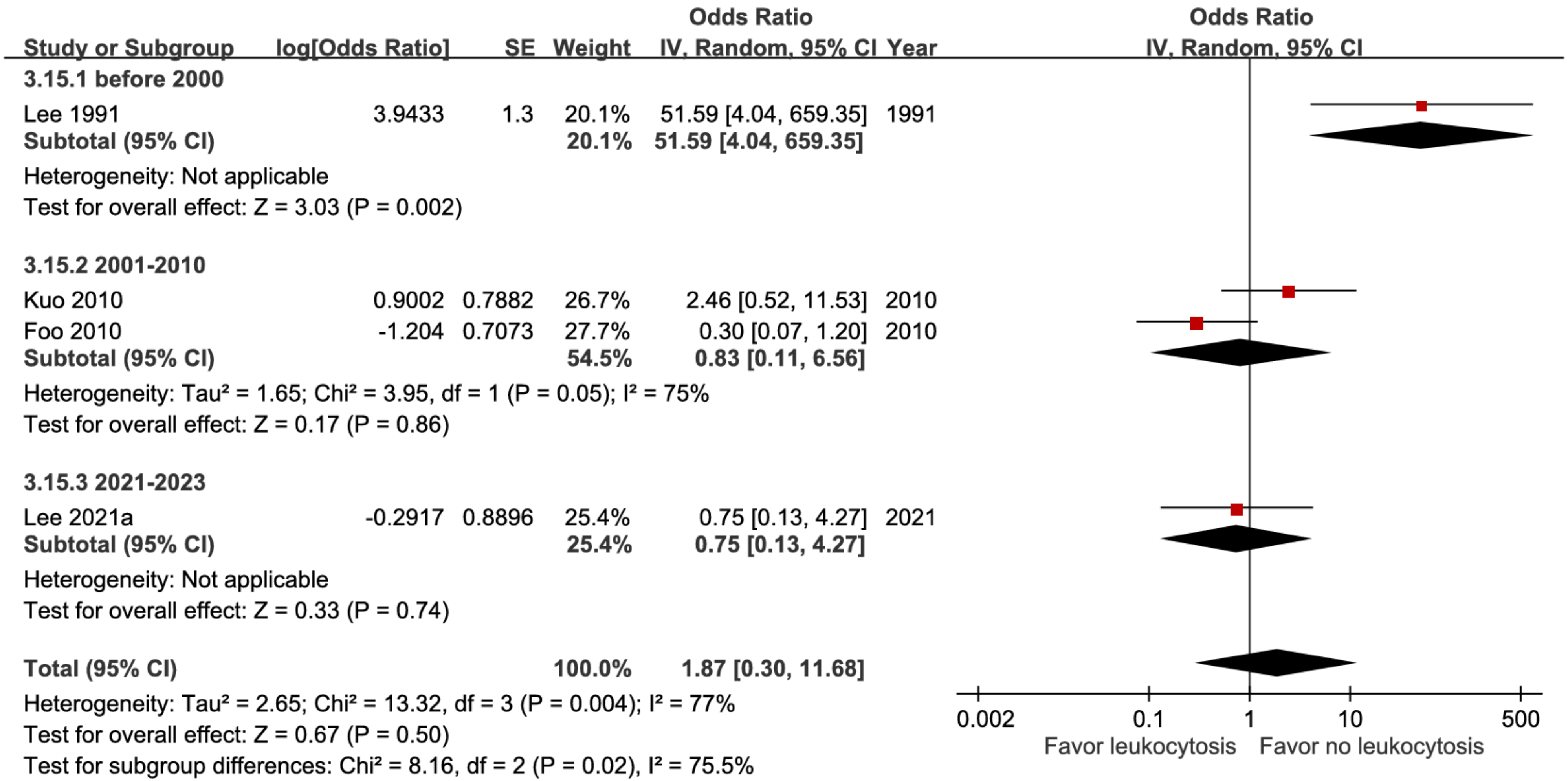

Figure S16. Forest plot of the association between leukocytosis (subgroup by year of publication) and short-term mortality in pyogenic liver abscess.

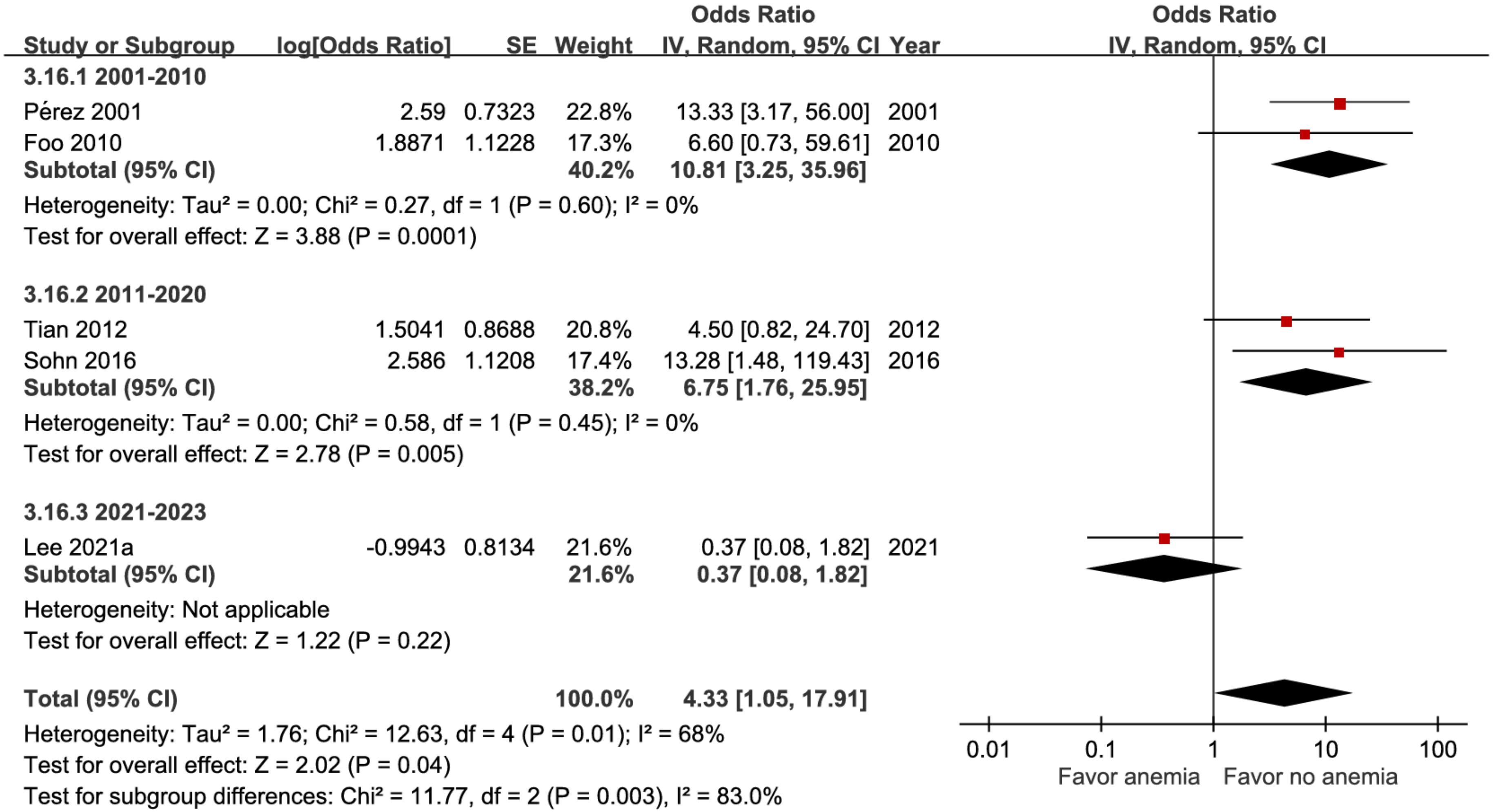

Figure S17. Forest plot of the association between anemia (subgroup by year of publication) and short-term mortality in pyogenic liver abscess.

Study or Subgroup	log[Odds Ratio]	SE	Weight	Odds Ratio IV, Random, 95% CI
**3.17.1 Platelet < 125K**				
Li 2023	2.0996	0.7313	24.6%	8.16 [1.95, 34.22]
Xu 2019b	1.0578	0.5226	48.1%	2.88 [1.03, 8.02]
**Subtotal (95% CI)**			**72.7%**	**4.28 [1.59, 11.52]**
Heterogeneity: Tau² = 0.14; Chi² = 1.34, df = 1 (P = 0.25); I² = 26%				
Test for overall effect: Z = 2.87 (P = 0.004)				
**3.17.2 Platelet < 140K**				
Sohn 2016	1.4807	0.6932	27.3%	4.40 [1.13, 17.10]
**Subtotal (95% CI)**			**27.3%**	**4.40 [1.13, 17.10]**
Heterogeneity: Not applicable				
Test for overall effect: Z = 2.14 (P = 0.03)				
**Total (95% CI)**			**100.0%**	**4.18 [2.05, 8.50]**
Heterogeneity: Tau² = 0.00; Chi² = 1.35, df = 2 (P = 0.51); I² = 0%				
Test for overall effect: Z = 3.94 (P < 0.0001)				
Test for subgroup differences: Chi² = 0.00, df = 1 (P = 0.97), I² = 0%				

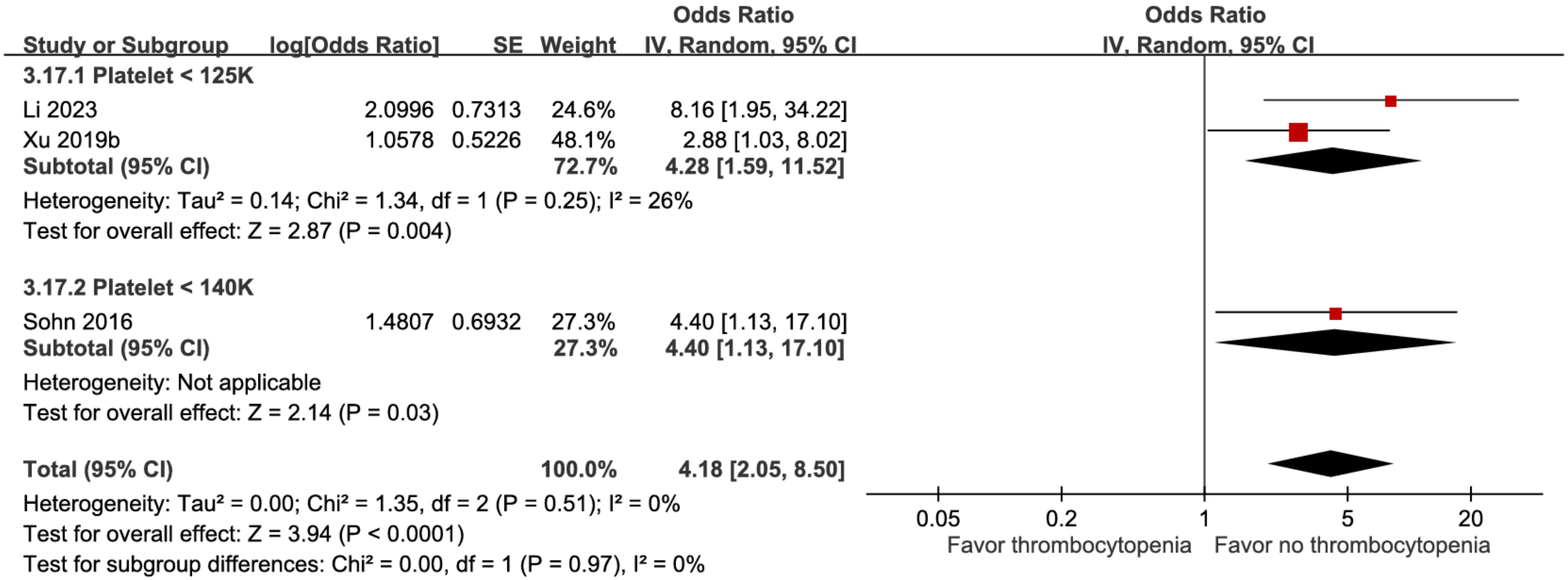

Figure S18. Forest plot of the association between thrombocytopenia (subgroup by cut-off value) and short-term mortality in pyogenic liver abscess.

(A)

Study or Subgroup	log[Odds Ratio]	SE	Weight	Odds Ratio IV, Random, 95% CI	Year
**3.18.1 albumin < 3.5**					
Tian 2012	1.8563	1.3465	3.1%	6.40 [0.46, 89.60]	2012
**Subtotal (95% CI)**			**3.1%**	**6.40 [0.46, 89.60]**	
Heterogeneity: Not applicable					
Test for overall effect: Z = 1.38 (P = 0.17)					
**3.18.2 albumin < 3.0**					
Kuo 2010	2.4519	1.0898	4.7%	11.61 [1.37, 98.28]	2010
Dai 2020	1.5151	0.6277	14.1%	4.55 [1.33, 15.57]	2020
Lee 2021a	1.4549	0.7756	9.2%	4.28 [0.94, 19.59]	2021
**Subtotal (95% CI)**			**28.0%**	**5.22 [2.18, 12.48]**	
Heterogeneity: Tau² = 0.00; Chi² = 0.65, df = 2 (P = 0.72); I² = 0%					
Test for overall effect: Z = 3.71 (P = 0.0002)					
**3.18.3 albumin < 2.8**					
Law 2013	1.2296	0.4758	24.5%	3.42 [1.35, 8.69]	2013
**Subtotal (95% CI)**			**24.5%**	**3.42 [1.35, 8.69]**	
Heterogeneity: Not applicable					
Test for overall effect: Z = 2.58 (P = 0.010)					
**3.18.4 albumin < 2.5**					
Lee 1991	2.4354	0.9695	5.9%	11.42 [1.71, 76.37]	1991
Chen 2005a	2.8154	1.3262	3.2%	16.70 [1.24, 224.69]	2005
Chou 2009	1.0253	0.3954	35.5%	2.79 [1.28, 6.05]	2009
**Subtotal (95% CI)**			**44.5%**	**5.25 [1.67, 16.47]**	
Heterogeneity: Tau² = 0.42; Chi² = 3.15, df = 2 (P = 0.21); I² = 36%					
Test for overall effect: Z = 2.84 (P = 0.004)					
**Total (95% CI)**			**100.0%**	**4.12 [2.60, 6.53]**	
Heterogeneity: Tau² = 0.00; Chi² = 4.39, df = 7 (P = 0.73); I² = 0%					
Test for overall effect: Z = 6.01 (P < 0.00001)					
Test for subgroup differences: Chi² = 0.58, df = 3 (P = 0.90), I² = 0%					

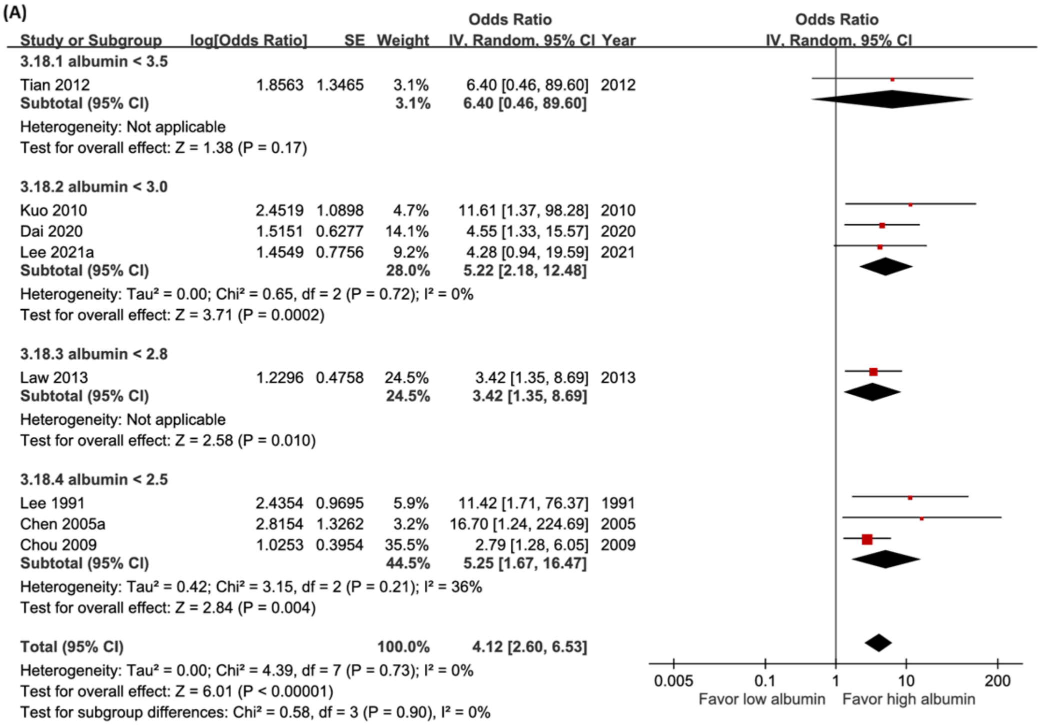

(B)

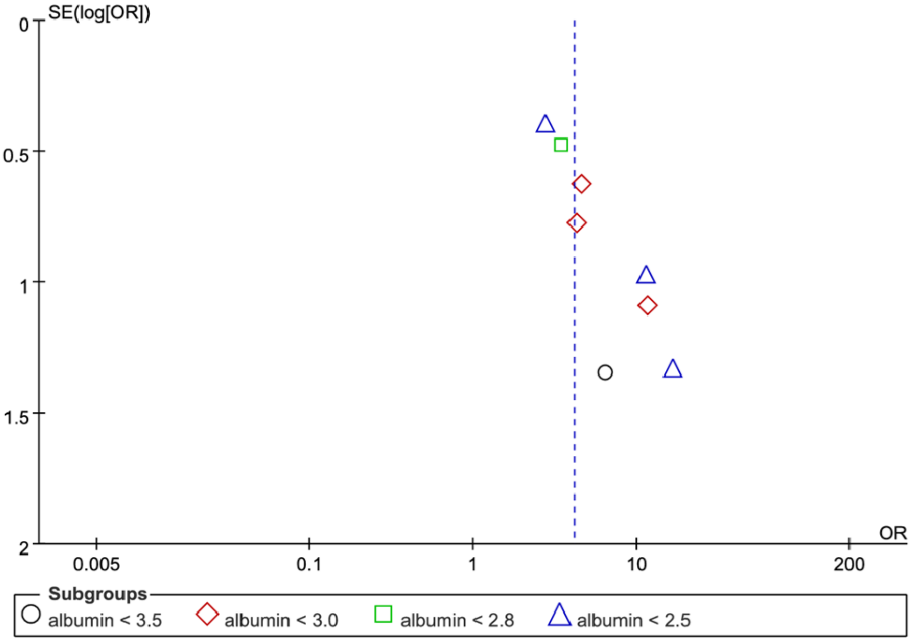

Figure S19. Forest and funnel plots of the association between hypoalbuminemia (subgroup by cut-off value) and short-term mortality in pyogenic liver abscess.

Study or Subgroup	log[Odds Ratio]	SE	Weight	Odds Ratio IV, Random, 95% CI	Year
**3.19.1 Sample size < 300**					
Pérez 2001	2.6735	0.8185	25.5%	14.49 [2.91, 72.08]	2001
Chen 2008a	3.4078	1.1809	21.0%	30.20 [2.98, 305.61]	2008
Shelat 2016	1.8924	0.5706	28.5%	6.64 [2.17, 20.30]	2016
**Subtotal (95% CI)**			**74.9%**	**10.16 [4.33, 23.85]**	
Heterogeneity: Tau² = 0.00; Chi² = 1.60, df = 2 (P = 0.45); I² = 0%					
Test for overall effect: Z = 5.33 (P < 0.00001)					
**3.19.2 Sample size > 300**					
Foo 2010	-1.204	0.8541	25.1%	0.30 [0.06, 1.60]	2010
**Subtotal (95% CI)**			**25.1%**	**0.30 [0.06, 1.60]**	
Heterogeneity: Not applicable					
Test for overall effect: Z = 1.41 (P = 0.16)					
**Total (95% CI)**			**100.0%**	**5.12 [0.84, 31.24]**	
Heterogeneity: Tau² = 2.67; Chi² = 15.10, df = 3 (P = 0.002); I² = 80%					
Test for overall effect: Z = 1.77 (P = 0.08)					
Test for subgroup differences: Chi² = 13.51, df = 1 (P = 0.0002), I² = 92.6%					

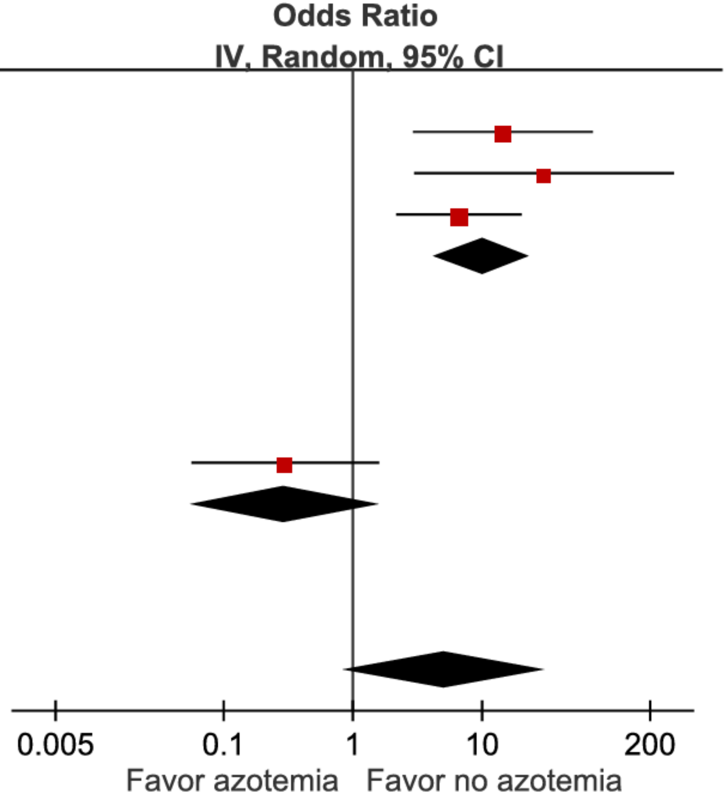

Figure S20. Forest plot of the association between azotemia (subgroup by sample size) and short-term mortality in pyogenic liver abscess.

Study or Subgroup	log[Odds Ratio]	SE	Weight	Odds Ratio IV, Random, 95% CI	Year
Chen 2007	1.4351	0.9494	17.7%	4.20 [0.65, 27.00]	2007
Foo 2010	0.9555	1.2646	10.0%	2.60 [0.22, 31.00]	2010
Ruiz-Hernández 2020	0.9594	0.4702	72.3%	2.61 [1.04, 6.56]	2020
**Total (95% CI)**			**100.0%**	**2.84 [1.30, 6.21]**	

Heterogeneity: Tau² = 0.00; Chi² = 0.21, df = 2 (P = 0.90); I² = 0%
Test for overall effect: Z = 2.61 (P = 0.009)

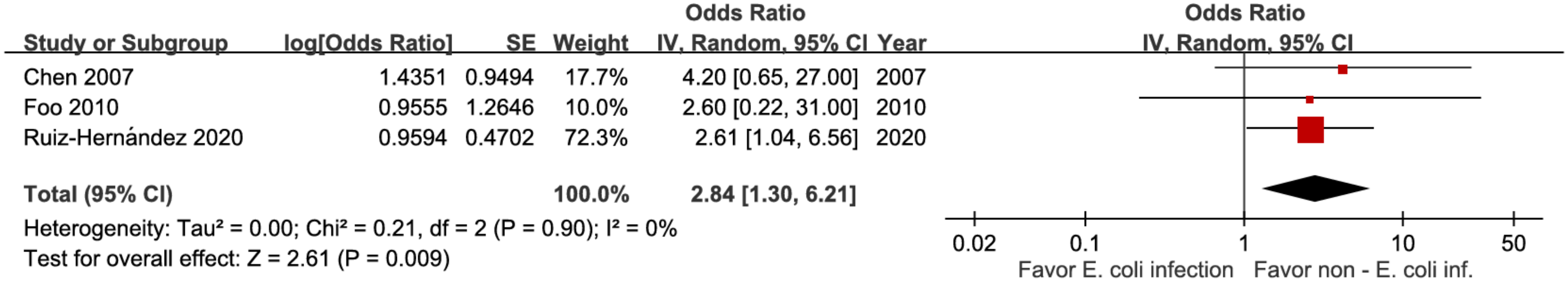

Figure S21. Forest plot of the association between *Escherichia coli* infection and short-term mortality in pyogenic liver abscess.

Study or Subgroup	log[Odds Ratio]	SE	Weight	Odds Ratio IV, Random, 95% CI	Year	Odds Ratio IV, Random, 95% CI
Chou 2009	0.0583	0.5537	35.6%	1.06 [0.36, 3.14]	2009	
Tian 2012	1.775	1.5566	9.8%	5.90 [0.28, 124.70]	2012	
Ruiz-Hernández 2020	0.2624	0.4722	39.6%	1.30 [0.52, 3.28]	2020	
Losie 2021	2.9178	1.1911	15.0%	18.50 [1.79, 191.01]	2021	
**Total (95% CI)**			**100.0%**	**2.09 [0.73, 5.97]**		

Heterogeneity: Tau² = 0.50; Chi² = 5.67, df = 3 (P = 0.13); I² = 47%
Test for overall effect: Z = 1.37 (P = 0.17)

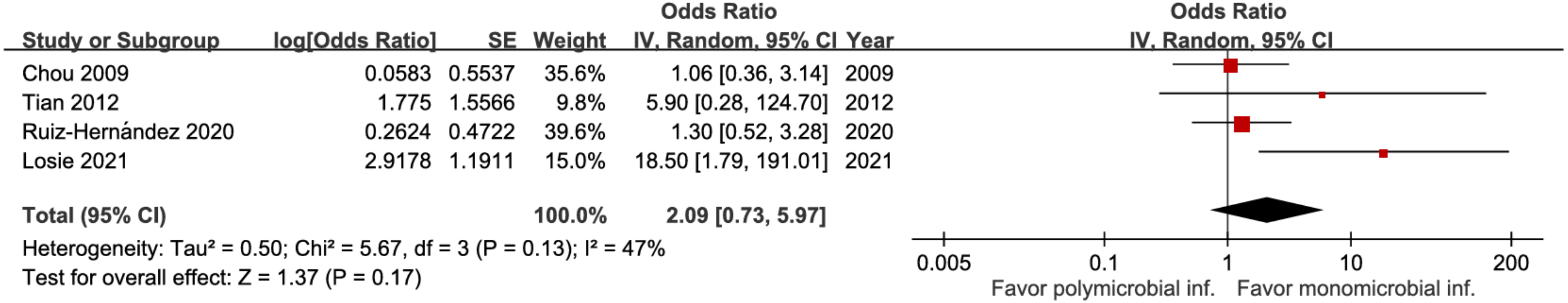

Figure S22. Forest plot of the association between polymicrobial infection and short-term mortality in pyogenic liver abscess.

Study or Subgroup	log[Odds Ratio]	SE	Weight	Odds Ratio IV, Random, 95% CI	Year
Tsai 2008	0.4161	0.0653	99.8%	1.52 [1.33, 1.72]	2008
Wu 2022	0.3386	1.4855	0.2%	1.40 [0.08, 25.79]	2022
**Total (95% CI)**			**100.0%**	**1.52 [1.33, 1.72]**	

Heterogeneity: $Tau^2 = 0.00$; $Chi^2 = 0.00$, df = 1 (P = 0.96); $I^2 = 0\%$

Test for overall effect: Z = 6.38 (P < 0.00001)

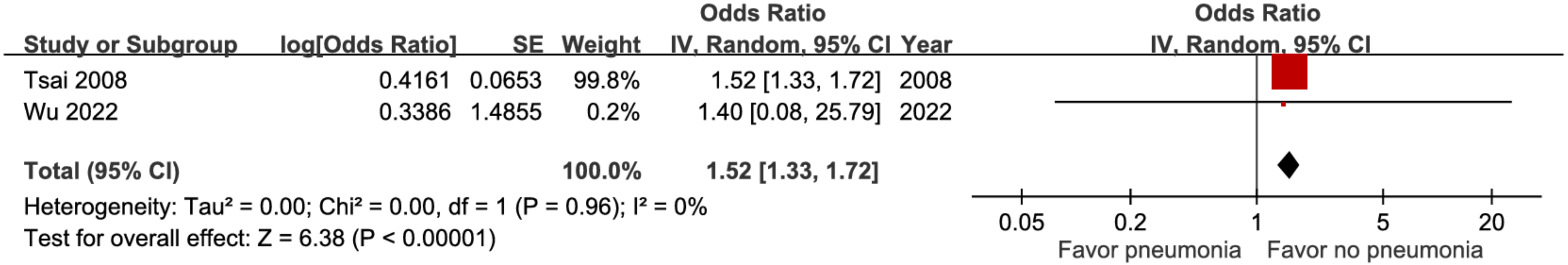

Figure S23. Forest plot of the association between pneumonia and short-term mortality in pyogenic liver abscess.

Study or Subgroup	log[Odds Ratio]	SE	Weight	Odds Ratio IV, Random, 95% CI	Year
Foo 2010	1.0296	1.1486	54.4%	2.80 [0.29, 26.60]	2010
Wu 2022	-1.6145	1.3893	45.6%	0.20 [0.01, 3.03]	2022
**Total (95% CI)**			**100.0%**	**0.84 [0.06, 11.07]**	

Heterogeneity: Tau² = 1.87; Chi² = 2.15, df = 1 (P = 0.14); I² = 54%

Test for overall effect: Z = 0.13 (P = 0.89)

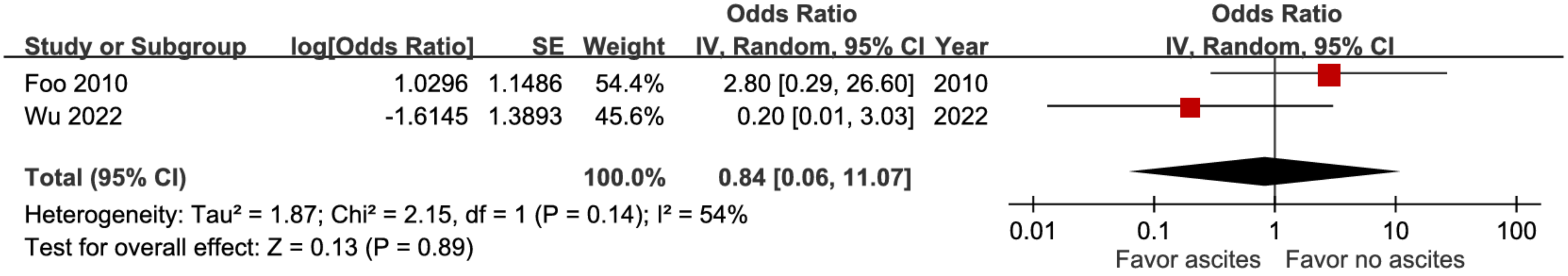

Figure S24. Forest plot of the association between ascites and short-term mortality in pyogenic liver abscess.

Study or Subgroup	log[Odds Ratio]	SE	Weight	Odds Ratio IV, Random, 95% CI	Year
Meddings 2010	-0.1393	0.1257	78.7%	0.87 [0.68, 1.11]	2010
Law 2012	0.131	0.2416	21.3%	1.14 [0.71, 1.83]	2012
**Total (95% CI)**			**100.0%**	**0.92 [0.74, 1.15]**	

Heterogeneity: $Tau^2 = 0.00$; $Chi^2 = 0.99$, df = 1 (P = 0.32); $I^2 = 0\%$
Test for overall effect: Z = 0.73 (P = 0.46)

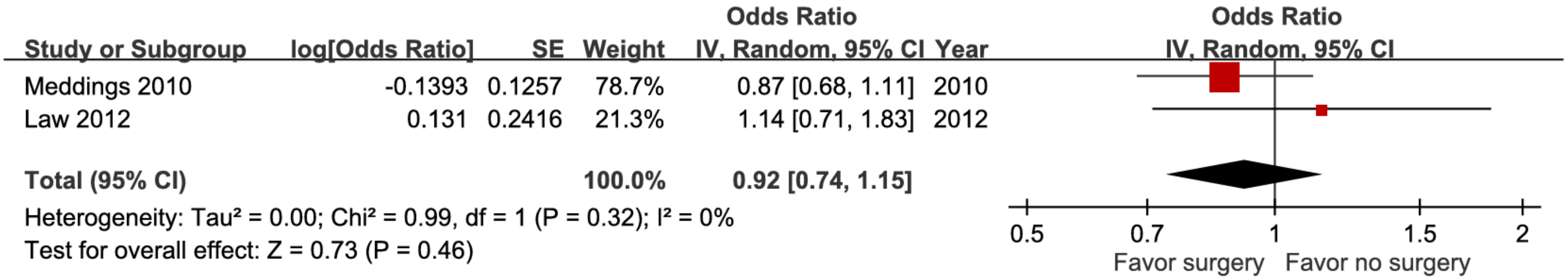

Figure S25. Forest plot of the association between surgical drainage and short-term mortality in pyogenic liver abscess.

Reference:

1. Al-Sayaghi KM, Alhujaily M, Zaky MK, et al. Percutaneous needle aspiration versus catheter drainage in the management of liver abscess: an updated systematic review and meta-analysis. *ANZ J Surg.* 2023;93:840-850.
2. Cai YL, Xiong XZ, Lu J, et al. Percutaneous needle aspiration versus catheter drainage in the management of liver abscess: a systematic review and meta-analysis. *HPB (Oxford).* 2015;17:195-201.
3. Chan KS, Chia CTW, Shelat VG. Demographics, Radiological Findings, and Clinical Outcomes of Klebsiella pneumonia vs. Non-Klebsiella pneumoniae Pyogenic Liver Abscess: A Systematic Review and Meta-Analysis with Trial Sequential Analysis. *Pathogens.* 2022;11.
4. Hussain I, Ishrat S, Ho DCW, et al. Endogenous endophthalmitis in Klebsiella pneumoniae pyogenic liver abscess: Systematic review and meta-analysis. *Int J Infect Dis.* 2020;101:259-268.
5. Lin JW, Chen CT, Hsieh MS, et al. Percutaneous catheter drainage versus percutaneous needle aspiration for liver abscess: a systematic review, meta-analysis and trial sequential analysis. *BMJ Open.* 2023;13:e072736.
6. Mahmoud A, Abuelazm M, Ahmed AAS, et al. Percutaneous catheter drainage versus needle aspiration for liver abscess management: an updated systematic review, meta-analysis, and meta-regression of randomized controlled trials. *Ann Transl Med.* 2023;11:190.
7. Riley RD, Moons KGM, Snell KIE, et al. A guide to systematic review and meta-analysis of prognostic factor studies. *Bmj.* 2019;364:k4597.
